# The intriguing molecular dynamics of Cer[EOS] in rigid skin barrier lipid layers requires improvement of the model

**DOI:** 10.1016/j.jlr.2023.100356

**Published:** 2023-03-21

**Authors:** Ferdinand Fandrei, Tomáš Havrišák, Lukáš Opálka, Oskar Engberg, Albert A. Smith, Petra Pullmannová, Norbert Kučerka, Veronika Ondrejčeková, Bruno Demé, Lucie Nováková, Miloš Steinhart, Kateřina Vávrová, Daniel Huster

**Affiliations:** 1 Institute of Medical Physics and Biophysics, University of Leipzig, Leipzig, Germany; 2 Skin Barrier Research Group, Faculty of Pharmacy, Charles University, Hradec Králové, Czech Republic; 3 Faculty of Pharmacy, Comenius University in Bratislava, Bratislava, Slovakia; 4 Institut Laue-Langevin, Grenoble, France; 5 Department of Analytical Chemistry, Faculty of Pharmacy, Charles University, Hradec Králové, Czech Republic; 6 Institute of Macromolecular Chemistry, Czech Academy of Science in Prague, Prague, Czech Republic

**Keywords:** stratum corneum models, NMR spectroscopy, neutron diffraction, lipid assembly, long periodicity phase, molecular dynamics, lipid chain order

## Abstract

Omega-*O*-acyl ceramides such as 32-linoleoyloxydotriacontanoyl sphingosine (Cer[EOS]) are essential components of the lipid skin barrier, which protects our body from excessive water loss and the penetration of unwanted substances. These ceramides drive the lipid assembly to epidermal-specific long periodicity phase (LPP), structurally much different than conventional lipid bilayers. Here, we synthesized Cer[EOS] with selectively deuterated segments of the ultralong *N*-acyl chain or deuterated or ^13^C-labeled linoleic acid and studied their molecular behavior in a skin lipid model. Solid-state ^2^H NMR data revealed surprising molecular dynamics for the ultralong *N-*acyl chain of Cer[EOS] with increased isotropic motion toward the isotropic ester-bound linoleate. The sphingosine moiety of Cer[EOS] is also highly mobile at skin temperature, in stark contrast to the other LPP components, *N-*lignoceroyl sphingosine acyl, lignoceric acid, and cholesterol, which are predominantly rigid. The dynamics of the linoleic chain is quantitatively described by distributions of correlation times and using dynamic detector analysis. These NMR results along with neutron diffraction data suggest an LPP structure with alternating fluid (sphingosine chain-rich), rigid (acyl chain-rich), isotropic (linoleate-rich), rigid (acyl-chain rich), and fluid layers (sphingosine chain-rich). Such an arrangement of the skin barrier lipids with rigid layers separated with two different dynamic “fillings” i) agrees well with ultrastructural data, ii) satisfies the need for simultaneous rigidity (to ensure low permeability) and fluidity (to ensure elasticity, accommodate enzymes, or antimicrobial peptides), and iii) offers a straightforward way to remodel the lamellar body lipids into the final lipid barrier.

## GRAPHICAL ABSTRACT

**Figure:**
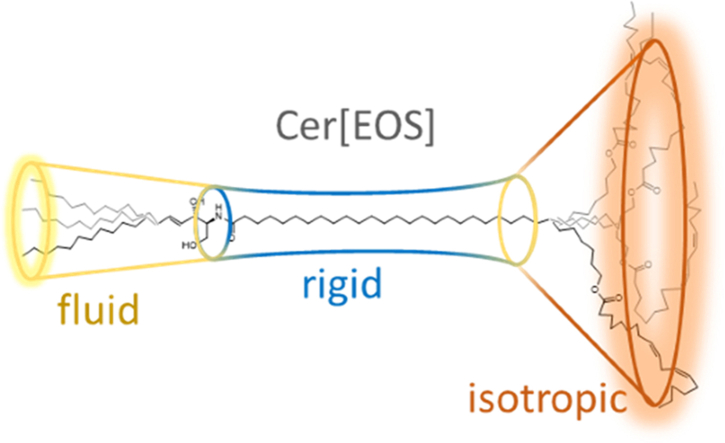

The *stratum corneum* (SC) is the outermost layer of the epidermis of the skin, a molecular compartment which is most relevant for establishing the skin barrier function (1). The major components of the SC are flattened dead corneocytes filled with keratin embedded in a layer of lipids, which is aligned approximately parallel to the cellular surface (Fig. 1). In human skin, three major classes of lipids form this layer in an approximate equal molar ratio, ceramides (Cer), FFAs, and cholesterol (Chol). The SC lipid phase contains very little water and the molecules have been described as highly rigid and densely packed, forming a crystalline layer as revealed by very extensive electron microscopy (2, 3, 4), X-ray and neutron diffraction (ND) studies (5, 6, 7, 8, 9, 10), Fourier transformed infrared (11) and solid-state NMR spectroscopy (12, 13, 14, 15) investigations and combinations of these methods (16, 17, 18, 19).

**Fig. 1:**
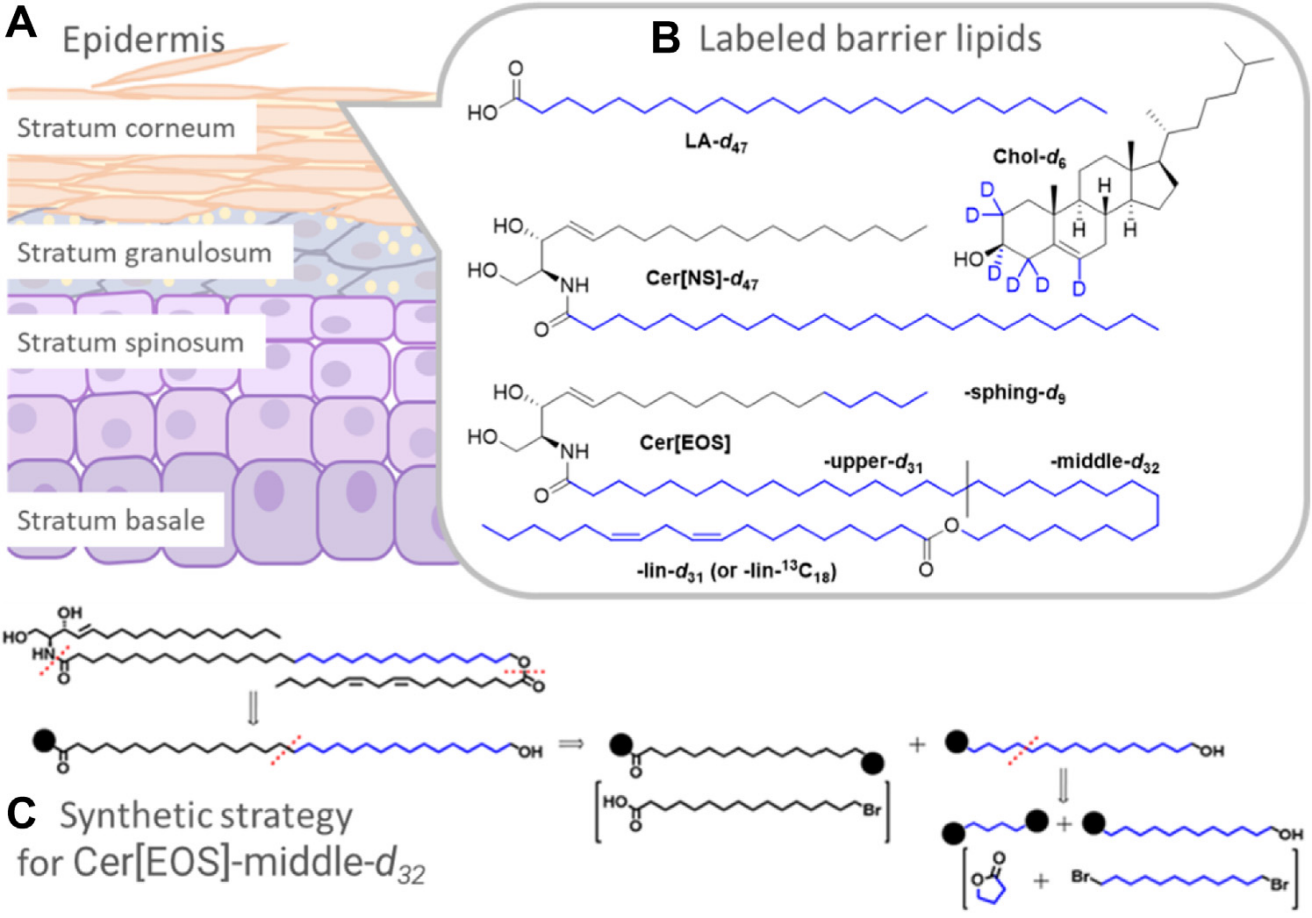
A: Cartoon representation of the human epidermis, B) chemical structures of the investigated lipid species with the deuterium-labeled segments in blue, lignoceric acid-*d*_47_ (LA-*d*_47_), cholesterol-*d*_6_ (Chol-*d*_6_), *N*-lignoceroyl-D-erythro-sphingosine-*d*_47_ (Cer[NS]-*d*_47_) and *N*-(32-linoleoyloxy)dotriacontanoyl-D-erythro-sphingosine (Cer[EOS]) labeled either at the sphingosine-*d*_9_ moiety (-sphing-*d*_9_), the upper acyl chain close to the headgroup (-upper-*d*_31_) or the middle part (-middle-*d*_32_) of the ultralong chain (black line indicating the border between upper and middle part) or at the linoleoyl-*d*_31_ moiety(-lin-*d*_31_). Cer[EOS] with ^13^C-labeled linoleate was also studied. Panel C) shows our synthesis strategy exemplified for deuterated Cer[EOS]-middle-*d*_32_.

While a lot of data has been collected from various methods to support a model of rigid molecules forming the densely packed orthorhombic lipid arrangement for establishing the proper barrier function of skin, very recently, new experimental findings also detected highly mobile lipid segments in SC model systems. This was especially true when the characteristic molecule *N*-(32-linoleoyloxy)dotriacontanoyl-D-erythro-sphingosine (Cer[EOS]) featuring an ultralong C32:0 ω-hydroxylated fatty acid with a diunsaturated C18:2 linoleic acid moiety attached to the ω-hydroxyl was present in the model mixtures. Cer[EOS] (for the structure, see Fig. 1) is an essential SC lipid and particularly relevant for forming the so-called long periodicity phase (LPP), which is characterized by a ∼13 nm X-ray repeat spacing found both in human SC specimen as well as Cer[EOS]-containing lipid model mixtures (3, 7, 20). A seminal ^2^H NMR study showed that a perdeuterated oleic acid moiety on the lower end of the ultralong ω-hydroxyl chain of Cer[EOS] was isotropically mobile even below physiological skin temperature (17). This is in agreement with the “sandwich model” proposed in 2001 (20), which describes the SC lipid layers as a crystalline phase of FFA and Cer in hairpin conformation, while a central layer is formed by a liquid phase of the highly mobile linoleic acid moiety undergoing completely isotropic motions (20). Further experimental support for a fluid core inside the densely packed SC lipid matrix came from magic-angle spinning (MAS) NMR data exploiting ^1^H and ^13^C NMR isotropic chemical shifts (21). A fluid slab was suggested by molecular dynamics (MD) simulations of Cer[EOS]-containing lipid mixtures (22).

Two other recent studies further pointed out that some SC lipid species may be subjected to significant MD, which appears to be an essential feature of the SC lipid phase. First, in a ^2^H NMR study, Engberg *et al.* (23) investigated the dynamics of the sphingosine moiety of *N*-lignoceroyl-D-erythro-sphingosine (Cer[NS]), the canonical ceramide, in a mixture in the absence of Cer[EOS] forming the short periodicity phase (SPP). Surprisingly, the sphingosine moiety also showed ^2^H NMR spectra that could only be explained by Cer[NS] undergoing axially symmetric reorientations about the long axis and undergoing large amplitude motions and/or 2-site exchange. Second, also in an SPP model, Fandrei *et al.* (24) showed that the presence of Chol sulfate, a minor sterol component of the SC lipid layers, induced a partial fluidization of the Chol molecules at physiological Chol sulfate concentration of 5 mol%.

Clearly, more experimental work is required to better understand the interplay of highly rigid and substantially mobile lipid molecules in the SC. Model mixtures can help addressing these important questions. Here, we continue studying the remarkable properties of Cer[EOS] in lipid models of the LPP phase. To this end, we synthesized Cer[EOS] species with specific ^2^H-labeling in the ultralong ω-hydroxyl acyl chain, providing site resolution for the upper (Cer[EOS]-upper-*d*_32_) or middle (Cer[EOS]-middle-*d*_32_) carbon segments in the C32:0 chain (see Fig. 1). Furthermore, Cer[EOS] with the physiologically relevant perdeuterated linoleic acid (Cer[EOS]-lin-*d*_31_) and ^13^C-labeled linoleic acid were synthesized and dynamically characterized. Finally, we also looked at the sphingosine moiety of Cer[EOS] with a partial deuteration of the last 4 carbons (Cer[EOS]-sphingo-*d*_9_) to provide dynamics information for this sphingosine chain segment as well. Taken together, our results provide a comprehensive dynamical characterization of Cer[EOS] in the physiologically relevant LPP allowing improvement of our model of the SC lipid phase that would be consistent with our biological understanding of the formation of the SC lipid phase from the lamellar bodies of the skin.

## MATERIALS AND METHODS

### Materials

Chol and Cer[NS] were purchased from Avanti Polar Lipids (Alabaster, AL). Chol(2,2,3,4,4,6)-*d*_6_ was purchased from Cambridge Isotope Laboratories, Inc. (Tewksbury, MA) and lignoceric acid-*d*_47_ (LA-*d*_47_), 1,12-dibromododecane-*d*_24_ and γ-butyrolactone-*d*_6_ were purchased from C/D/N Isotopes, Inc. (Pointe-Claire, Canada). *N*-(32-linoleoyloxy)dotriacontanoyl-D-erythro-sphingosine (Cer[EOS]) (Fig. 1) was synthesized as described in the literature (25). Cer[EOS]-sphing-*d*_9_ was purchased from Matreya LLC (State College, PA). The synthesis of Cer[NS]-*d*_47_ was a one-step reaction of the sphingosine with LA as similarly performed before (26). Palmitic acid, stearic acid, arachidic acid, behenic acid and LA (all analytical or HPLC grade) were purchased from Sigma-Aldrich Chemie GmbH (Schnelldorf, Germany). Chemicals and solvents for synthesis were purchased from Merck (Darmstadt, Germany), VWR International (Stribrna Skalice, Czech Republic), and Penta Chemicals (Prague, Czech Republic) and were used without further purification. Linoleic acid-*d*_32_ and ^13^C_18_ were purchased from Merck (Darmstadt, Germany).

### Synthesis

The synthetic pathways and characterization of the intermediates and final compounds can be found in the Supporting Information. Reactions were monitored using TLC aluminium plates with silica 60 F254 (Merck, Darmstadt, Germany) with detection using a solution of Ce(SO_4_)_2_ and H_3_[P(Mo_3_O_10_)_4_] in sulfuric acid. Silica gel 60 (0.04–0.063 mm, Carl Roth, Karlsruhe, Germany) was used for column chromatography. ^1^H and ^13^C NMR spectra were recorded on Varian VNMR S500 (Palo Alto, CA) and Jeol JNM-ECZ600R (Tokyo, Japan) spectrometers. Chemical shifts were reported as δ values in parts per million (ppm) and were indirectly referenced to tetramethylsilane via the solvent signal. Melting points were measured on a Kofler apparatus and are uncorrected. Infrared spectra were measured on a Nicolet 6700 spectrometer in the attenuated total reflection mode (Thermo Fisher Scientific, Waltham, MA). A UHPLC system Acquity UPLC I-class (Waters, Milford) coupled to a high-resolution mass spectrometer Synapt G2Si (Waters, Manchester, UK) based on Q-TOF was used for high-resolution mass spectrometer spectra measurement. Chromatography of less lipophilic species was carried out using a Acquity UPLC ethylene bridged hybrid C18 (2.1 x 50 mm, 1.7 mm) column and gradient elution with acetonitrile and 0.1% formic acid at a flow-rate of 0.4 ml/min. Chromatography of more lipophilic species was carried out using the same column but different gradient elution with mobile phase A (acetonitrile:methanol:isopropanol:formic acid 1%) and mobile phase B (isopropanol) at a flow-rate of 0.35 ml/min. ESI was operated in positive mode. The ESI spectra were recorded in the range 50–1200 m/z using leucine-enkefalin as a lock mass reference and sodium formate for mass calibration.

### Sample preparation

Lipid mixtures consisting of Cer, FFAs, and Chol were prepared in a 1:1:0.45 M ratio. The ceramide fraction was formed by a mixture of 30 mol% Cer[EOS] and 70 mol% Cer[NS24]. The fatty acid component of the mixture consisted of a mix of saturated FFAs with ratios of C16:0 (1.8%), C18:0 (3.9%), C20:0 (7.5%), C22:0 (47.8%), and C24:0 (39.0%) (27). Each sample contained one of the deuterated lipids. Thus, structural and dynamic parameters for each deuterated component of the mixture could be studied individually. For sample preparation, the lipids were dissolved in chloroform/methanol (2:1) and mixed at the appropriate molar ratio. Subsequently, the solvent was evaporated using a rotary evaporator and redissolved in cyclohexane before lyophilization at approx. 0.1 mbar resulting in a fluffy powder. The lipid powder was hydrated with 50 wt% aqueous buffer prepared with deuterium depleted water at pH = 5.4 (100 mM 2-(*N*-morpholino)ethanesulfonic acid, 100 mM NaCl, 5 mM EDTA). After 10 freeze-thaw cycles (freezing of the sample in liquid nitrogen, heating to 80°C in a water bath) the samples were filled into 4 mm MAS rotors and incubated at room temperature for a minimum of 24 h to allow for proper sample equilibration. The NMR rotors were sealed with an airtight cap to prevent dehydration of the ^2^H NMR samples.

### X-ray scattering measurements

Small- and near wide-angle X-ray scattering (SAXS/NWAXS) measurements were performed using a point-focusing SAXS instrument (originally Molecular Metrology, recently considerably upgraded by SAXSLAB, now Xenocs) at room temperature. Rigaku Micromax-003, a low-power micro source equipped with X-ray optics working at *U* = 50 kV and *I* = 0.6 mA generated a CuKα beam with a wavelength of λ = 0.154 nm. Scattering was detected by the 2D detector Pilatus3 300K (Dectris) at 0.449 m and 0.0573 m from the sample. Merging data from these two distances allowed reaching reliable q interval from 0.13 to 35 nm^-1^, where q is the magnitude of the scattering vector q = 4π sin(θ)/λ and θ is half of the scattering angle. Samples were sealed in 1.5 mm-diameter quartz capillaries (the scattering of an empty capillary was not subtracted from the scattering curves of samples). Sample to detector distance was calibrated using silver behenate or Si standard. Data were azimuthally averaged and adjusted to an absolute scale using a glassy carbon standard.

### Solid-state ^2^H NMR spectroscopy

Stationary ^2^H NMR measurements were carried out using a Bruker Avance 750 WB NMR spectrometer (Bruker BioSpin, Rheinstetten, Germany). The ^2^H resonance frequency was 115.15 MHz for ^2^H using and a solid probe with a 5 mm solenoid coil was used. We applied a spectral width of ±250 kHz using quadrature phase detection using the standard phase cycled quadrupolar echo pulse sequence (28) (the length of the 90° pulses was 2.5–3.5 μs 90°, pulses were separated by a 30 μs delay). The recycle delay was 50 s for samples that showed very broad components from orthorhombic phases to ensure full relaxation to thermal equilibrium to enable quantification of the proportion of the individual phases (15). Samples were investigated at temperatures of 25, 32, 50, and 65°C and processed using a program written in Mathcad (MathSoft, Cambridge, MA) (29). Selected samples were remeasured to confirm reproducibility.

### ^2^H NMR line shape simulations

The time domain data of the ^2^H NMR spectra were simulated using a superposition of a number of n*|*N ^2^H NMR Pake doublets with a quadrupolar coupling constant of 167 kHz, scaled by an order parameter (*S*_CD_) as described previously (13). For the terminal methyl group, a scaling by one-third due to the three-site hopping was assumed. Usually, one isotropic line also needed to be used in the simulations. The relative proportions of each individual phase observed in the ^2^H NMR spectra were directly determined from the simulations. Full quantification of the ^2^H NMR spectra was possible because of the long delay times of 50 s between successive scans (approximately 5 × *T*_1_, *T*_1_ ∼ 10 s) (13, 14, 15). The relative contributions of the different phases were determined from the area under the curve for each individual ^2^H NMR spectrum adjusted manually.

We have also performed a Monte Carlo analysis for the ^2^H NMR spectra having multiple phases. We took the original experimental spectrum and add randomly generated noise to it, and subsequently fitted the result. This process was repeated 600 times for each spectrum, after which we evaluated the standard deviation of the weight of each phase. Details of the procedure is given in the Supporting Information.

### ^1^H and ^13^C MAS NMR spectroscopy

Solid-state ^1^H and ^13^C MAS NMR spectra were acquired at a Bruker Avance NEO 700 MHz NMR spectrometer (Bruker BioSpin, Rheinstetten, Germany) operating at a resonance frequency of 176.0 MHz for ^13^C and 700.1 MHz for ^1^H and equipped with an E-free H/C/N probe with a 3.2 mm spinning module. All measurements were recorded at a MAS of 7000 Hz, temperature of 32°C and a 4 μs π/2 pulse.

For the ^1^H NMR spectra, we applied a Hahn echo sequence with a relaxation delay of 5 s. For the ^13^C NMR directly excited NMR spectra, a Hahn pulse echo sequence with a relaxation delay of 5 s and ^1^H decoupling was used (ω_H_/2π = 62.5 kHz). For the cross polarization (CP) spectra we used an ^1^H excitation pulse of 4 μs with a CP contact time of 700 μs and a ^1^H CP spin lock field of ∼40 kHz. During acquisition, a ∼62 kHz SPINAL64 decoupling was applied and spectra were recorded with a recycle delay of 2.5 s. All NMR spectra were calibrated relative to tetramethylsilane at 0 ppm.

### ^13^C MAS NMR relaxation measurements

All NMR relaxation measurements were acquired at 32°C. Solid-state ^13^C NMR spectra were acquired on a Bruker Avance III 600 MHz NMR spectrometer operating at a ^13^C resonance frequency of 150.9 MHz for ^13^C and at a Bruker Avance I 400 MHz spectrometer (^13^C resonance frequency of 100.6 MHz for ^13^C). Either a 3.2 mm or a 4 mm MAS probe head was used. *Τ*_1_, nuclear Overhauser effect (NOE), and *Τ*_1*ρ*_ experiments were carried out at the 600 MHz, and *T*_1_ and NOE experiments at the 400 MHz NMR spectrometer. Relaxation experiments at both 400 MHz and 600 MHz were acquired using 6 kHz MAS rate, 4 μs ^13^C π/2-pulses (62.5 kHz) for direct excitation, and acquisition was performed using Waltz64 for ^1^H decoupling at 17 kHz (30). For details on the relaxation and NOE measurements, please refer to the Supporting Information.

### Relaxation measurement data analysis

^13^C NMR relaxation data were processed in Bruker Topspin and relaxation rate constants were extracted from the resulting spectra using INFOS (31). The resulting relaxation rate constants (2 *T*_1_, 2 NOE, 2 *T*_1*ρ*_) were processed with the detector analysis (32) using the pyDIFRATE software (https://github.com/alsinmr/pyDR), to yield 6 detector windows (analysis archived at Github (https://doi.org/10.5281/zenodo.7347151). Additional details on processing and the relaxation rate constants and resulting fits found in the Supporting Information.

### Neutron diffraction

The samples for the ND experiment were prepared from ≈ 17 mg or 10 mg of the protonated or deuterated lipid mixture, respectively. The dried lipid mixtures were redissolved in hexane/ethanol (96%) = 2:1 (v/v) at the saturated concentration at 50°C. The lipid solution was spread in few layers on a silicon wafer (Crystal GmbH, Berlin, Germany) preheated to 50°C. The traces of the solvents were removed in a vacuum overnight and the sample was annealed at 70 °C in the presence of water vapor for 30 min with subsequent slow cooling to the room temperature. ND data (10.5291/ Institut Laue-Langevin (ILL)-DATA.9-13-825) were collected at the small-angle diffraction instrument D16 located at the ILL (Grenoble, France). The neutron radiation (λ = 0.449 nm, Δλ/λ = 0.01) was selected by a graphite monochromator consisting of nine Highly Oriented Pyrolytic Graphite crystals (mosaic spread 0.4°) set in vertical sample-focusing geometry and collimated by four pairs of motorized slits, two at the monochromator and the other two at the sample, yielding a beam dimension of 25.2 mm vertically × 6.2 mm horizontally.

Diffraction patterns were collected using on D16 the ILL-built Millimeter-resolution Large Area Neutron Detector, a flat, high-pressure ^3^He neutron detector with an area of 320 mm x 320 mm and a “pixel” resolution of 1 mm x 1 mm. The experiments were conducted using the high-precision BerILL humidity chambers developed at ILL (33) to control in situ the sample hydration and temperature. The samples were placed in the humidity chamber and prealigned offline using a laser-based setup and then equilibrated at 32°C and constant humidity for 6 h before the measurement. The contrast conditions used were 100, 70, 40, and 8% ^2^H_2_O (ILL, Grenoble, France) in H_2_O (v/v) at 100% relative humidity. The rocking curves (Ω-scans) of the samples were recorded by rotating the sample position by steps of 0.05° in an Ω-range of −1°–12° and by steps of 0.1° in an Ω-range of 10°–16° at fixed detector γ-positions of 10.8° and 26°, respectively, covering up to the 9th order Bragg reflection. The corresponding acquisition time for a single sample at one contrast condition was ≈ 3 h. The detected 2D images were converted to one-dimensional diffraction patterns as a function of the scattering angle 2θ and normalized to the incoming flux and acquisition time. The scattering form factors *F(h)* (34) and their uncertainties were calculated (35). For further details, see Supporting Information and (36).

## RESULTS

### Synthesis of labeled Cer[EOS]

**Scheme 1:**
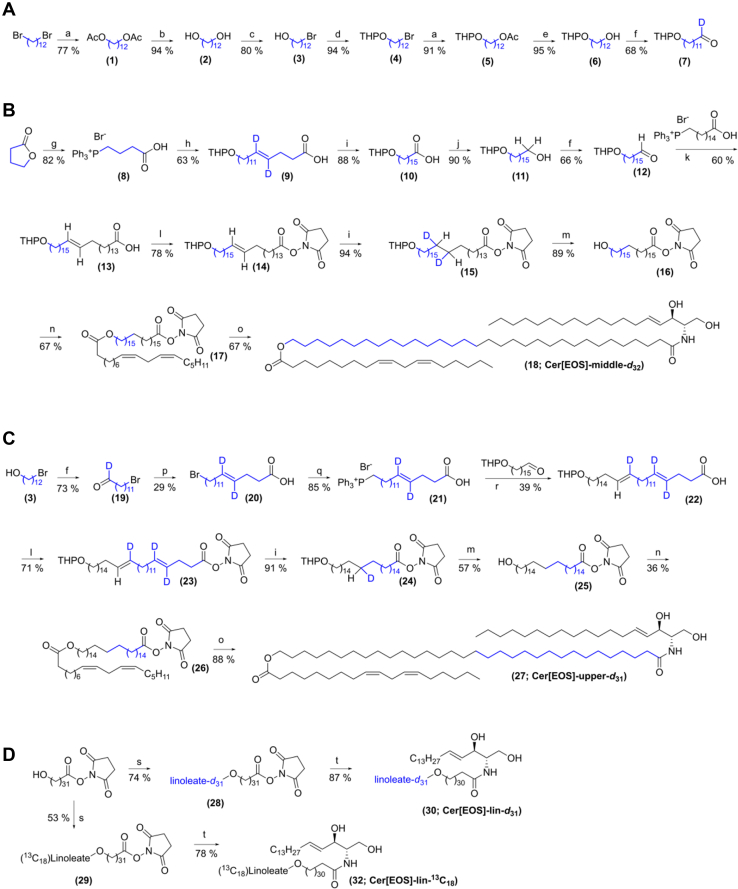
Synthesis of labeled Cer[EOS]. Preparation of aldehyde precursor (7) (A), Cer[EOS]-middle-*d*_32_ (B), Cer[EOS]-upper-*d*_31_ Cer[EOS] (C), and linoleate-labeled Cer[EOS] (D). Reaction conditions: **a** NaOAc, DMF, 100°C, 3 h; **b** DOWEX 50, MeOH, reflux, overnight (ON); **c** 48% HBr, toluene, reflux, 90 min; **d** PPTS, 3,4-dihydro-2*H*-pyran, CHCl_3_, reflux, 3 h; **e** LiAlH_4_, THF, 0°C–RT, ON; **f** (COCl)_2_, DMSO, TEA, DCM, −60°C–RT, ON; **g** PPh_3_·HBr, 190°C, 3 h (37); **h (7)**, NaHMDS, THF, −30°C–RT, ON; **i** D_2_, Pd/C (10%), EtOAc, RT, ON; **j** BH_3_·THF, THF, 0°C–RT, ON; **k** (15-carboxypentadecyl)triphenylphosphonium bromide (25), NaHMDS, THF, −20°C–RT, ON; **l** disuccinimidyl carbonate, DIPEA, DCM, RT, ON; **m***p*-toluenesulfonic acid, MeOH, RT, ON; **n** linoleic acid, 2,4,6-trichlorobenzoyl chloride, TEA, DMAP, THF, RT, ON; **o** sphingosine, DIPEA, THF, DCM, RT, ON; p (8), NaHMDS, THF, −30°C–RT, ON; **q** PPh_3_, 140°C, ON; **r** 16-[(tetrahydro-2*H*-pyran-2-yl)oxy]hexadecanal (25), NaHMDS, THF, −20°C–RT, ON; **s** 2,5-dioxopyrrolidin-1-yl 32-hydroxydotriacontanoate (25), linoleic acid-*d*_32_ (or linoleic acid-^13^C_18_), 2,4,6-trichlorobenzoyl chloride, TEA, DMAP, THF, RT, ON; **t** sphingosine, DIPEA, THF, DCM, RT, ON. For abbreviations and reaction details, see Supporting Information.

To investigate the properties of Cer[EOS] ultralong *N-*acyl chain, we first synthesized two Cer[EOS] analogs with deuteration of the 15 protonated carbons proximal to the polar head (Cer[EOS]-upper-*d*_31_) and the 16 carbons proximal to linoleate (Cer[EOS]-middle-*d*_32_), respectively (Fig. 1). Our first goal was to construct the specifically labeled 32C chain from suitable 1,16-disubstituted 16C fragments; however, such deuterated compounds are not available. Thus, retrosynthetic analysis (see example in Fig. 1) led us to 4C and 12C fragments and γ-butyrolactone-*d*_6_ and 1,12-dibromododecane-*d*_24_ were identified as suitable starting materials. The final synthetic pathways are shown in Scheme 1. All reaction steps were first optimized using nondeuterated compounds.

The first synthetic steps were focused on breaking the symmetry of 1,12-dibromododecane-*d*_24_ as a direct substitution of a single bromine was unsatisfactory. Thus, 1,12-dibromododecane was converted to diol (2) in two steps and the key asymmetric precursor (3) for both target lipids was obtained using concentrated HBr in toluene. This seemingly inefficient pathway gave better yields of (3) than a direct conversion. The alcohol (3) was converted in four steps to a protected aldehyde (7) (Scheme 1A), which underwent a Wittig reaction with a phosphonium salt (8) prepared by a direct opening of γ-butyrolactone with triphenylphosphonium bromide (37). The resulting unsaturated acid (9) was deuterated and converted in two steps to aldehyde (12) for Wittig reaction with previously prepared phosphonium salt (25) to construct the ultralong chain acid (13). At this step, it was advantageous to convert the acid to succinimidyl ester (14) for three reasons: maintaining good solubility of the ultralong compound, protecting the acid during linoleate attachment, and activating carboxyl to react with sphingosine. Next, the double bond was reduced, the ω-hydroxyl was deprotected, and linoleate was attached using Yamaguchi esterification (38). Cer[EOS]-middle-*d*_32_ was obtained by a direct acylation of sphingosine with the activated ester (17). The overall yield of this 17-step procedure was approximately 2% (Scheme 1B).

The synthesis of Cer[EOS]-upper-*d*_31_ followed a similar logic. The precursor (3) was oxidized to aldehyde (19) and, using phosphonium salt (8), converted to unsaturated bromo acid (20). The acid (20) immediately reacted with PPh_3_ to provide phosphonium salt (21) for the Wittig reaction with a 16C nondeuterated aldehyde (25). The ultralong compound (22) was activated, deuterated, deprotected, esterified with linoleic acid and attached to sphingosine to provide Cer[EOS]-upper-*d*_31_ in 12 steps with 0.5% overall yield (Scheme 1C).

### Small- and near wide-angle X-ray scattering

**Fig. 2:**
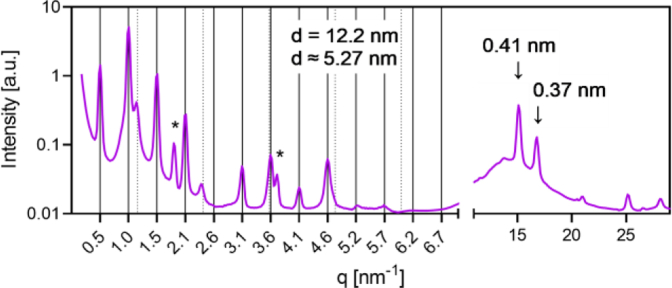
The SAXS/NWAXS scattering curves of the representative Cer[EOS]/Cer[NS]/FFA/Chol mixture at the molar ratio of 0.3/0.7/1/0.45 in the small- (left) and near wide- (right) scattering angle region. The full grid lines predict the peak positions of the phase with *d* = 12.2 nm, dotted grid lines predict the peak positions of the phase with *d* = 5.27 nm, and asterisks indicate the peaks of separated Chol. The peaks typical for the orthorhombic polymethylene chain packing are marked by arrows. NWAXS, near wide-angle X-ray scattering; SAXS, small-angle X-ray scattering.

The simplified lipid model of the skin barrier was composed of the Cer[EOS]/Cer[NS]/FFA/Chol mixture at the molar ratio of 0.3/0.7/1/0.45. This lipid composition has been previously proven to form predominantly the LPP with the repeat distance *d* of 12.2 nm (39). The reduced amount of Chol relative to its natural abundance (1) prevented the excessive formation of Chol crystals in the sample. The nanostructure of the lipid models was investigated after the ^2^H NMR spectroscopy measurements using SAXS/NWAXS at room temperature. A representative scattering curve (Fig. 2) of the Cer[EOS]/Cer[NS] /FFA/Chol model showed a sequence of regularly spaced peaks of LPP (full grid lines, *d* = 12.2 nm), a minor fraction of a SPP (dotted grid lines, *d* ≈ 5.3 nm) and peaks of the separated crystalline Chol (asterisks). The near WAXS region contained peaks corresponding to the repeat distance of adjacent scattering planes of 0.41 and 0.37 nm, which are characteristic of the orthorhombic packing of polymethylene lipid chains (40). This tight arrangement is possible for the crystalline domains of polymethylene chains, which are not perturbed by incorporated Chol molecules. We have identified the LPP with *d* = 12.00–12.91 nm in the models with or without deuterated components.

### Investigation of the dynamics and phase state of the main lipids in the Cer[EOS]/Cer[NS]/FFA/Chol mixture by ^2^H NMR at varying temperatures

**Fig. 3:**
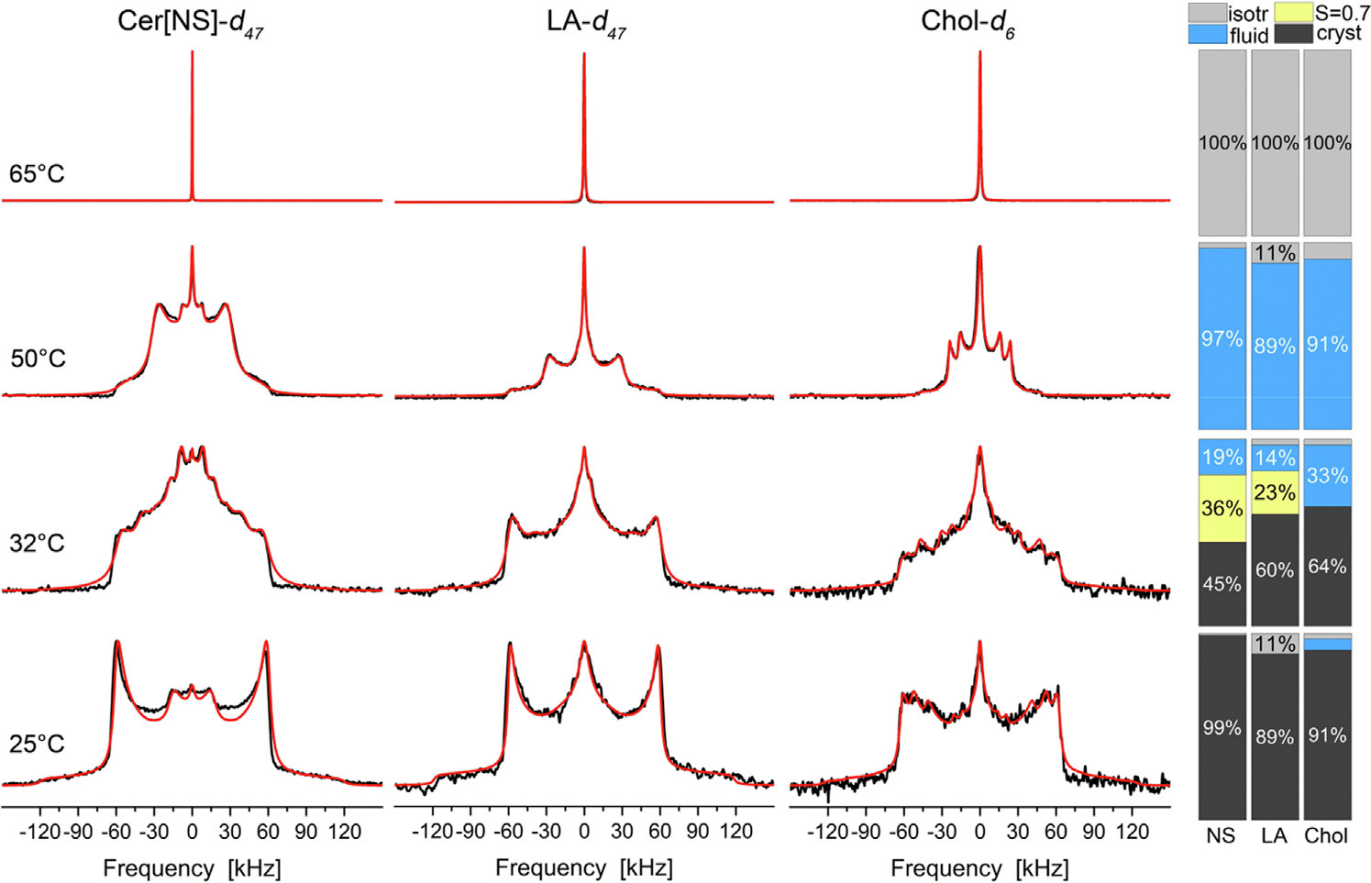
^2^H NMR spectra of the individually ^2^H-labeled molecules in the Cer[EOS]/Cer[NS24]/FFA-mix/Chol mixture (0.3/0.7/1/0.45 M ratio) at varying temperatures hydrated with 50 wt% buffer. From left to right, the figure shows the ^2^H NMR spectra of the deuterated acyl chain of Cer[NS]-*d*_47_, the deuterated chain of LA-*d*_47_ of the FFA mix, and the partially deuterated Chol-*d*_6_ in the right column. The bar plots on the far right report the phase proportions derived from the numerical simulations of the ^2^H NMR line shapes (red lines). The ^2^H NMR spectra were acquired with a repetition time of 50 s to achieve full relaxation of all components, allowing quantification. LA, lignoceric acid.

In the next step, we analyzed the thermotropic phase behavior of the three most abundant lipids in the studied mixture, i.e., Cer[NS], LA, and Chol. The static ^2^H NMR spectra of these molecules at four different temperatures are shown in Fig. 3. Each column represents the ^2^H NMR spectra of one specifically deuterated lipid (either Cer[NS]-*d*_47_, LA-*d*_47_, or Chol-*d*_6_) and each row shows a measurement at a given temperature between 25°C (bottom row) and 65°C (top row). Thus, the figure provides an overview of the thermotropic phase behavior and the dynamics for the ^2^H-labeled lipids in the mixture.

At 25°C, Cer[NS], LA, and Chol show ^2^H NMR spectra characteristic for the completely rigid orthorhombic phase of SC mixtures (13, 15, 17). Only the terminal –CH_3_ groups of the lipid acyl chains undergo three-site rotations and some small isotropic contributions are also detected. A small fraction of the Chol is also fluid and isotropically mobile, respectively, revealed from rather narrow quadrupolar splittings in the ^2^H NMR spectra as discussed in detail before (41, 42). The respective phase proportions can be quantified by numerical line shape simulations, which have been carried out for each ^2^H NMR spectrum and are shown as red lines in the ^2^H NMR spectra shown in Fig. 3. We used manual adjustment of the various phase contributions to simulate the ^2^H NMR spectra as well as an automated Monte Carlo-based fitting procedure. Both approaches yielded similar results and are presented in supplemental Table S1. The bar plots on the far right report the relative proportions as derived from the manual simulations. Below, we discuss the results from the manual fitting procedure.

At the physiological skin temperature of 32°C, we observe some alterations in the ^2^H NMR spectra of the mixture. However, the majority of the lipids yield a lineshape characteristic of the solid state, as required for maintaining the barrier function of the skin. This state is described predominantly by a crystalline phase and for LA and Cer[NS] and a somewhat mobile phase indicated by a chain order parameter of *S* = 0.7, indicating that the molecules are not undergoing axially symmetric reorientations. The crystalline phase accounts for 45, 60, and 64% of Cer[NS], LA, and Chol, respectively, while the mobile phase accounts for 36% of the Cer[NS] and 23% of the LA. Additionally, all lipids show a partial fluid behavior characterized by axially symmetric motions about the long axis of the molecules accounting for 19% and 14% for Cer[NS] and LA, respectively. For Chol, this fluid phase is even more pronounced, accounting for about 33% of the whole Chol fraction.

Increasing the temperature to 50°C leads to a significant restructuring of the lipid mixture. All deuterated components of the mixture form a relatively mobile phase where the lipids undergo axially symmetric rotational diffusion about their long axis. This state accounts for 97% of the Cer[NS], 89% of the LA, and 91% of the Chol. The molecular properties of the lipids in this mobile phase can be compared to lipids in a liquid ordered phase state as known from lipid raft mixtures (43, 44, 45, 46). While the molecules undergo axially symmetric reorientations, the lipid chains largely remain in an all-*trans* state (43). This gives rise to ^2^H NMR spectra that are characterized by a single quadrupolar splitting for the methylene segments with a width of 62.6 kHz (corresponding to a C-H order parameter of *S* = 0.5). The methyl groups are of course subject to three-site rotations which scales their quadrupolar splitting by an additional one-third. This is exactly the situation encountered for the ^2^H NMR spectra of Cer[NS] and LA. Previously, characteristic ^2^H NMR spectra of a liquid-crystalline phase have been reported for similar SC lipid mixtures at temperatures between 50 and 65°C (13, 17, 47, 48). We extended our NMR measurements to these temperatures but did not observe any ^2^H NMR spectra indicative of such a phase state. The Chol molecules are showing ^2^H NMR spectra that can be explained by an upright orientation and axially symmetric reorientations in the membrane as explained in detail before (41, 42). Further increase of the temperature to 65°C converts all lipid components into an isotropic phase.

### Investigation of the dynamic properties of Cer[EOS] in the mixture by ^2^H NMR spectroscopy

**Fig. 4:**
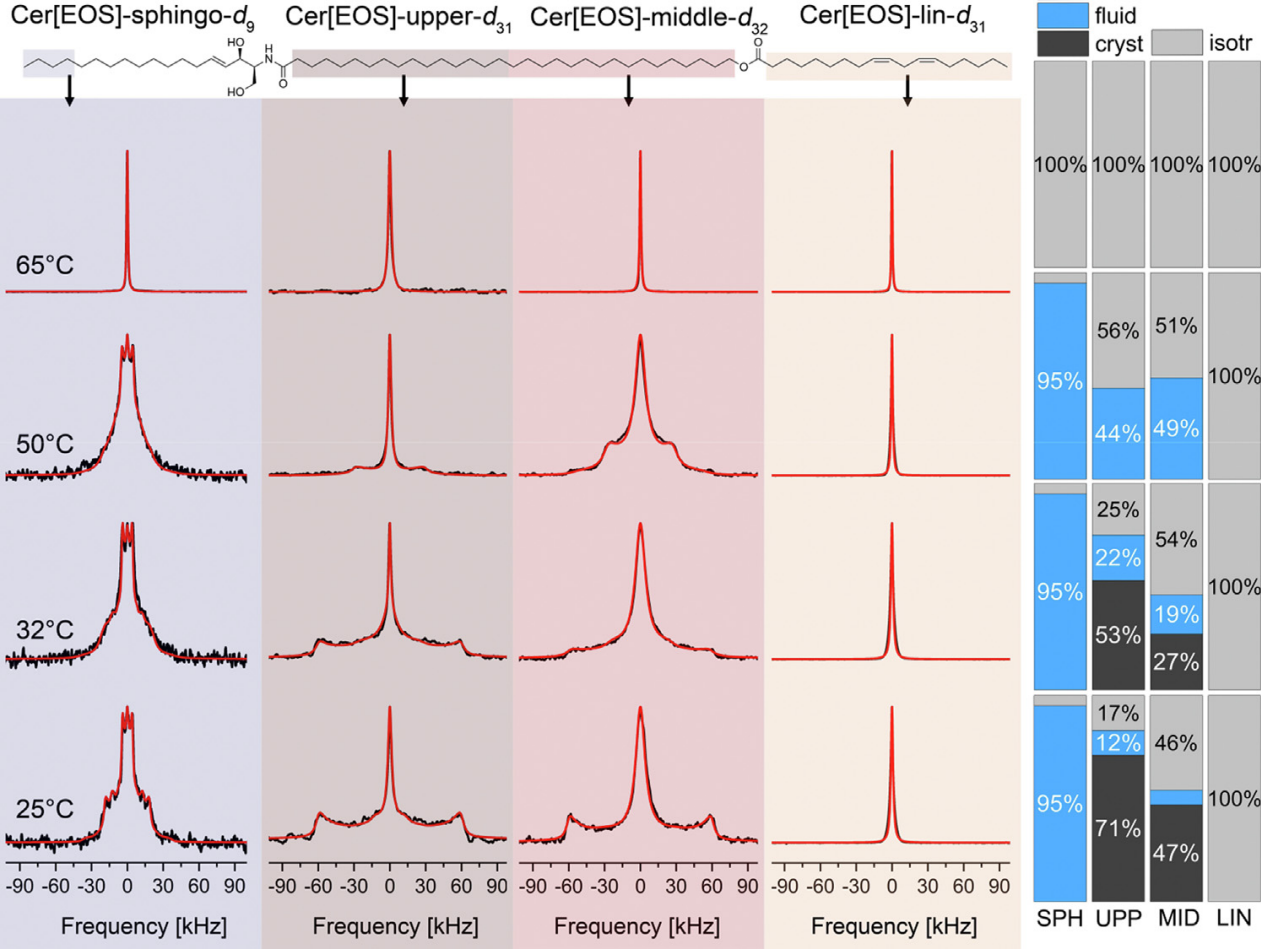
^2^H NMR spectra of the individually ^2^H-labeled Cer[EOS] segments (see Fig. 1) in a Cer[EOS]/Cer[NS24]/FFA-mix/Chol mixture (0.3/0.7/1/0.45 M ratio) at varying temperatures hydrated with 50 wt% buffer. From left to right column, the figure shows the ^2^H NMR spectra of the partially deuterated sphingosine chain of Cer[EOS] (-sphingo-*d*_9_) in light purple background, the upper (brown) and the middle (red) half of the deuterated ω-hydroxyacyl chain of Cer[EOS] (-upper-*d*_32_ or -middle-*d*_32_) and the deuterated linoleoyl moiety (orange) of Cer[EOS]-lin-*d*_31_ in the right column. The spectra were acquired with a repetition time of 50 s, to achieve full relaxation of all components, allowing quantification. The red lines illustrate the best-fit numerical simulations. Phase compositions are extracted from these simulations and visualized in bar plots on the far right of the figure.

In contrast to the relatively homogeneous crystalline behavior of Chol, Cer[NS], and LA at 25°C and 32°C, the lipid chains of Cer[EOS] show a strikingly heterogeneous and more dynamic behavior (Fig. 4). Astonishingly, the ^2^H NMR spectrum of the linoleic acid in Cer[EOS]-lin-*d*_31_ (right column) only exhibits an isotropic line at all observed temperatures indicating that the linoleoyl part of Cer[EOS] undergoes completely isotropic reorientation within the crystalline SC lipid matrix. Such a remarkable behavior has also been reported before for Cer[EOS] species featuring the less physiologically relevant oleic acid modification of the ultralong ω-hydroxyl fatty acid (17).

Next, we drew our attention to the fully saturated C32:0 part of the ultralong chain of Cer[EOS]. To this end, we synthesized Cer[EOS] molecules with separately deuterated upper and middle parts of the acyl chain. The first molecule features a deuteration in the upper chain segment close to the headgroup referred to as Cer[EOS]-upper-*d*_31_. The second molecule has the deuteration in the middle part of the chain consisting of the 16 deuterated carbons close to the linoleoyl moiety and is referred to as Cer[EOS]-middle-*d*_32_. The ^2^H NMR spectra of both halves of the C32:0 chain are shown in Fig. 4. These spectra are more reminiscent of the NMR spectra of the other lipids of the mixture (Cer[NS] and LA). At 25°C, both parts of the ultralong chain are in a partially crystalline state but also fluid spectral components are identified. The relative proportions of these contributions vary between the upper and the middle acyl chain segments. While 71% of the upper chain are crystalline, only 47% of the middle part are in this rigid state. It is remarkable that significant isotropic contributions are detected accounting for 17% for the upper and 46% for the middle sections, complemented by smaller fluid contributions (12% for the upper and 7% for the middle sections).

A temperature increase to physiological conditions (32°C) reduces the crystalline proportions of the NMR spectra to 53% and 27% while increasing the fluid proportions to 22% and 19% for the upper and middle segments of the C32:0 chain, respectively. The isotropic contributions also increase slightly to 25% and 54%, respectively, for the two segments. Further, temperature increase to 50°C completely abolishes all crystalline phases. The fluid contributions increase to 44% for the upper and to 49% for the middle segments of the ultralong chain. The rest is in an isotropic phase for both parts of the chain. A temperature increase to 65°C converts all chain segments to a fully isotropic state.

Finally, we investigated the dynamic behavior of the sphingosine moiety of Cer[EOS] using a variant with the last four carbons deuterated (Cer[EOS]-sphing-*d*_9_). The ^2^H NMR spectrum of Cer[EOS]-sphing-*d*_9_ (left column in Fig. 4) is dominated by sharp Pake doublets with ∼10 kHz quadrupolar splitting for temperatures between 25°C and 50°C representing the terminal methyl peak of the sphingosine chain and indicating a dominating fluid phase. Furthermore, resolved peaks with larger quadrupolar splitting are observed especially at 25°C from the deuterated methylene chain segments. These spectral features are very characteristic for a liquid-ordered state as well known from phospholipid bilayers (44, 45, 46). Ceramides have long been studied in relation to their properties to induce phase separation in complex lipid mixtures (49, 50, 51, 52). The observed quadrupolar splittings can be converted into order parameters ranging between 0.20 and 0.35. These findings confirm that the sphingosine moiety of Cer[EOS] undergoes large amplitude motions with decreasing order for the methylene groups further down the lipid chain also suggesting a mainly fluid phase for the sphingosine part. These findings are in very good agreement with previous results for Cer[NS] in lipid mixtures forming the SPP not containing Cer[EOS] (23). It should be noted that about 5% of the sphingosine is isotropically mobile and no crystalline phase is present in the terminal sphingosine chain. At higher temperatures (≥65°C) also the sphingosine part of Cer[EOS] shows exclusively an isotropic spectral pattern.

### Investigation of the molecular properties of Cer[EOS] in the mixture by ^1^H and ^13^C MAS NMR spectroscopy

**Fig. 5:**
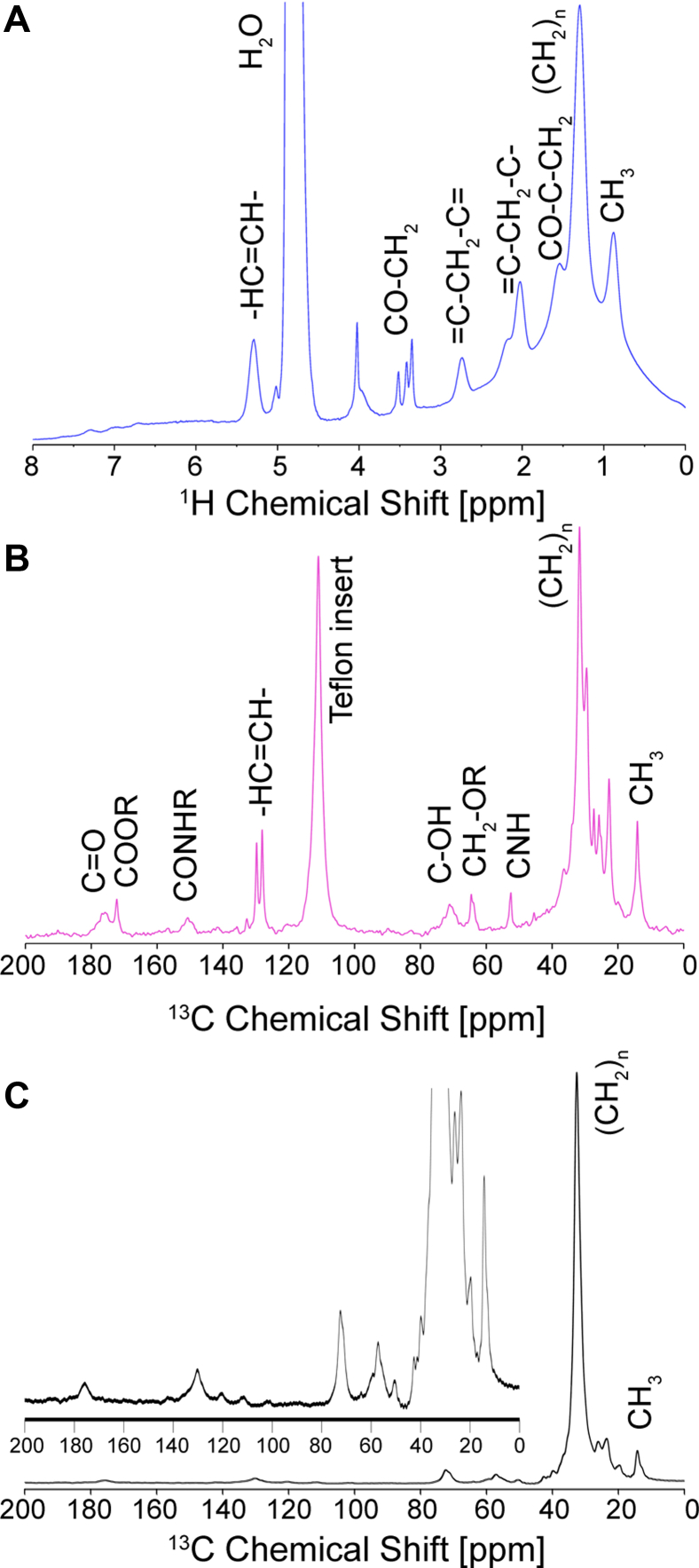
^1^H and ^13^C MAS NMR spectra of a mixture of Cer[EOS]/Cer[NS24]-d_47_/FFA-mix/Chol (0.3/0.7/1/0.45 M ratio) under MAS (7 kHz) conditions at 32°C hydrated with 50 wt% buffer. A: shows the mixtures ^1^H NMR spectrum. B: shows the mixtures direct excited ^13^C NMR spectrum. C: shows the ^13^C CP NMR spectrum. CP, cross-polarization; MAS, magic-angle spinning.

In addition to ^2^H NMR, MAS NMR has recently become a useful tool in the investigation of the SC (12, 53, 54). Figure 5A shows the ^1^H NMR spectrum of the Cer[EOS]/Cer[NS]/FFA/Chol lipid mixture. Intriguingly, the spectrum features sharp signals indicative of highly mobile lipids typically not observed in rigid lipid structures (43). ^1^H MAS NMR spectra of the mixture feature a superposition of well-resolved isotropic peaks from highly mobile lipids (55) along with a very broad background stemming from the crystalline lipid segments. Moderate MAS frequencies are not sufficient to average out all anisotropic contributions to ^1^H MAS NMR spectra and predominantly detect highly mobile lipids (56, 57).

All of the sharp NMR peaks can be explained by mobile hydrocarbon chain signals from the unsaturated linoleic chain, namely by the protons of the double bonds at 5.5 ppm, the methylene peaks next to the double bonds (at 2.0 ppm), and the peak at 2.75 ppm, which represents the protons of the methylene group in between the double bonds of the linoleoyl moiety of Cer[EOS] (29). Other relatively well-resolved signals can be assigned to methylene groups of the linoleic acid chains (at 1.3 and 2.5 ppm) and the terminal methyl groups at 0.9 ppm.

Better resolution is achieved in the ^13^C MAS NMR spectra, which can be acquired in direct excitation mode (Fig. 5B) to highlight the mobile lipids and by CP in combination with high power decoupling to predominantly detect the rigid lipids (58). For the directly excited ^13^C NMR spectrum, typical features of highly mobile hydrocarbon chains are visible, which agree with linoleic acid chain segments (signals at ∼130 ppm). Also, some broader peaks are assigned to the headgroups of the lipids, i.e., amide groups (∼150 ppm), CNH groups (∼50 ppm), and the alcohol groups (∼70 ppm) as well as the carbonyls (∼175 ppm). It is uncertain if the broader peaks are a result of the superposition of multiple functional groups or the higher ordered state of the respective headgroups. Another sharper peak could be assigned to the ω-carbon of the ultralong chain of Cer[EOS] (∼65 ppm). In contrast, the ^13^C CP MAS NMR spectrum of the mixture (Fig. 5C) that predominantly detects the rigid lipid segments is dominated by the signals of the chain methylenes indicating the crystalline state of most lipids in the mixture.

### Detector analysis of the ^13^C NMR relaxation rates

**Fig. 6:**
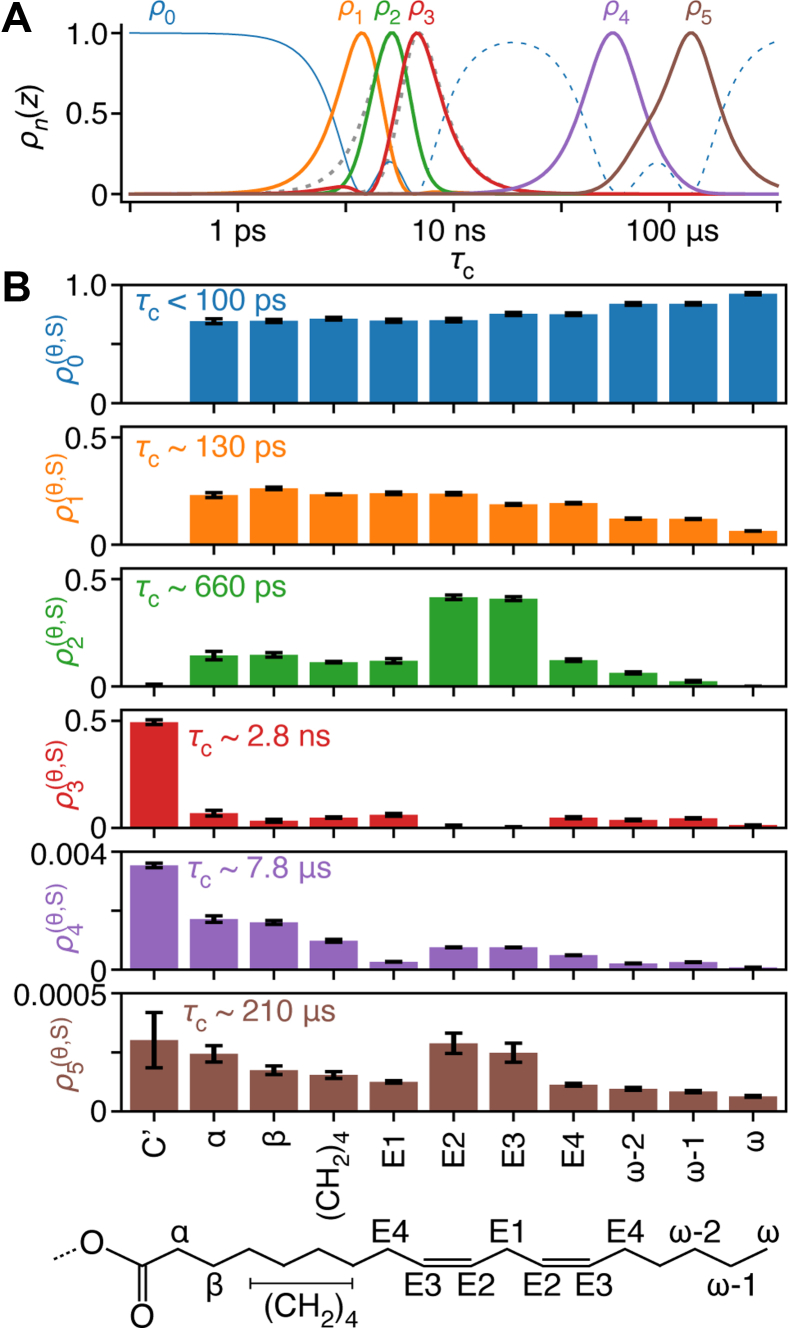
Detector analysis of the linoleoyl chain of Cer[EOS]. A: plots the sensitivity windows for six detectors (solid, color). For the C′ position, we do not have H–C NOE data and cannot verify if C′ is isotropic since it does not appear in deuterium spectra. Then, only *ρ*_2_–*ρ*_5_ are defined for C′ and *ρ*_2_, *ρ*_3_ have slightly different sensitivities, as indicated by the gray dashed lines. *ρ*_0_ is indicated with a solid line for short correlation times and dashed for longer correlation times, since it is unlikely that this response results from slow motion. B: shows the six detector responses as a function of position in the chain (*x*-axis), where each bar indicates the amplitude of reorientational motion with the corresponding sensitivity window. The approximate timescale is shown in each plot and the linoleoyl chain is shown below, with each *x*-axis label indicated on the chain.

^2^H NMR spectra of the linoleoyl chain indicate that the motion of all carbons (except the carbonyl (C′)) is isotropic (Fig. 6). However, there is still variation in the dynamic behavior as we move down the chain. Measurement of ^13^C NMR relaxation (heteronuclear NOE, *T*_1_, *T*_1*ρ*_, see supplemental Fig. S1) allows the timescale-specific characterization of reorientational motion with *detectors* (59). Detectors provide several timescale-specific windows (Fig. 6A), where we obtain detector responses ($\rho_{n}^{\left(\theta ,S\right)}$) for each ^13^C resonance and each window (Fig. 6B). Each detector provides dynamics information on a specific range of correlation times. The responses quantify the amplitude of reorientational motion within each timescale window (i.e., the contribution of motion on each timescale to 1–*S*^2^). Fit of experimental data is shown in supplemental Fig. S2.

From $\rho_{0}^{\left(\theta ,S\right)}$, we see that all positions except C′ are dominated by fast motion (<100 ps), where $\rho_{0}^{\left(\theta ,S\right)}$ increases toward the end of the chain (supplemental Figs. S3 and 6). The increase in $\rho_{0}^{\left(\theta ,S\right)}$ is accompanied by decreases in motion around 130 ps ($\rho_{1}^{\left(\theta ,S\right)})$, which may be interpreted as an increase in mobility (i.e., fast motion as we move down the chain). Motion at C′ is markedly slower than all other positions: $\rho_{3}^{\left(\theta ,S\right)}$ (∼2.8 ns) for C′ is ∼7 times larger than all other positions. Note that we do not have NOE or deuterium data for C′, which prevents us from calculating $\rho_{0}^{\left(\theta ,S\right)}$ and $\rho_{1}^{\left(\theta ,S\right)}$ and it may also be that C′ is not fully isotropic. However, we expect that $\rho_{1}^{\left(\theta ,S\right)}$ would be small, given that $\rho_{2}^{\left(\theta ,S\right)}$ is nearly zero whereas $\rho_{3}^{\left(\theta ,S\right)}$ is relatively large, suggesting dynamics around 3 ns or slower for the carbonyl. Slower motion for C′ is not surprising, given that deuterium spectra of the preceding positions in the acyl chain are not completely isotropic and $\rho_{3}^{\left(\theta ,S\right)}$ highlights the sharpness in the change in mobility as a function of position. $\rho_{4}^{\left(\theta ,S\right)}$ and $\rho_{5}^{\left(\theta ,S\right)}$ capture dynamics around 10 and 200 μs, respectively, which are expected to be highly collective motions. Note that these responses result from motion of the residual H–C dipole tensor, so that their amplitudes indicate a very small residual H–C dipole tensor, which decreases in size toward the end of the chain. Outliers at the double bonds (E2, E3) likely indicate larger collective motions; these responses are consistent with our recent study of POPC membranes, (60) although the significance of these larger responses has not been fully understood.

### Neutron diffraction

The Cer[EOS]/Cer[NS]/FFA/Chol and Cer[EOS]/Cer[NS]-d_47_/FFA/Chol mixtures at the molar ratio of 0.3/0.7/1/0.45 were measured by ND at 32°C and 100% relative humidity. The model containing Cer[NS]-d_47_ was measured at four different contrast conditions (8, 40, 70, and 100% of ^2^H_2_O). The protonated model with Cer[NS] was measured at 40% ^2^H_2_O because its structure was less well resolved but even repeatedly prepared samples did not provide better resolution. The ND curves of both samples are in the Supporting Information (supplemental Figs. S4 and S5). The ND results can be used to reconstruct the relative neutron scattering length density (NSLD) profile of the structure in real space with the possibility of distinguishing the position of deuterated moiety across the repeating unit.

The form factors *F*(*h*) for individual orders *h* were used to reconstruct the relative NSLD profiles. For a centrosymmetric structure (corresponds more to mirror symmetry in canonical crystallography), the phase angles for individual *F*(*h*) attain the values of either 1 or -1 (henceforward denoted as + or -). For the deuterated sample, the functions of |*F*(*h*)| versus ^2^H_2_O content were treated with a linear regression function (supplemental Fig. S6), and the phase angles were determined according to the method of Franks and Lieb (61). The phase angles of the protonated Cer[EOS]/Cer[NS]/FFA/Chol sample, measured only at one contrast condition, were determined according to the previously published data: ND of a similar LPP model with *d* = 12.9 nm, albeit having more complex Cer composition, was measured at 50% ^2^H_2_O (62). The absolute values of form factors *F* (1), *F* (2), *F* (3), *F* (4), and *F* (6) had a similar distribution of amplitudes relative to those determined for our sample Cer[EOS]/Cer[NS]/FFA/Chol. The reconstructed relative NSLD profiles of the protonated and the deuterated sample (supplemental Fig. S7) agreed with the previously published data (62). The protonated sample showed NSLD maxima at *d*/2 and at the distance *z* = 2.1 nm from the center of the repeat unit. The low values of NSLD formed pits in the middle of the unit and on both sides of the unit. The low values of NSLD are related to regions of hydrophobic protonated polymethylene chains without the presence of ^2^H_2_O, whereas the ^2^H_2_O and deuterated moieties increase the NSLD. The sample with Cer[NS]-*d*_47_ (in all three variants, supplemental Fig. S7) showed the maximal NSLD in the middle of the repeating unit. Comparing the lineshapes of these NSLD profiles, a considerable fraction of the deuterated Cer[NS]-*d*_47_ acyl was localized in the center of the unit. The ^2^H NMR spectra indicated that the Cer[NS]-*d*_47_ acyl chains are in the crystalline state. Therefore, the central pit in the NSLD profile of Cer[EOS]/Cer[NS]/FFA/Chol should be formed by tightly packed rigid polymethylene chains with predominant hydrophobic van der Waals interactions. The ^2^H NMR spectra proved that the individually ^2^H-labeled Cer[EOS] segments are embedded in three domains with different degrees of rigidity, fluid, crystalline, and isotropic. An effort to accommodate the crystalline, fluid, and isotropic domains in the structural model in an uncomplicated way led us to abandon the Franks and Lieb model for the reconstruction of the NSLD profiles.

The more model-independent approach assumes that the structure is centrosymmetric but does not presume the water distribution across the NSLD profile to be the error function that would lead to the alternation of slope signs of the contrast varied *F(h)* (34). We calculated relative NSLD profiles of the protonated sample from the first five nonzero *F*(*h*) factors using all possible combinations of the phase angles (supplemental Fig. S8A–D). The most likely profile of the protonated sample (phase angles - - + - 0 -), measured at 40% ^2^H_2_O, provided narrow peaks of high NSLD at ≈ 4.0 nm from the center of the unit (supplemental Fig. S8A). Another smaller local maximum in the NSLD was found in the center of the unit at *z* = 0 nm. Thus, the relative NSLD distribution formed narrow pits at the positions *z* = *d*/2 nm and a broad pit with a central slab at *z* = 0 nm. The narrow and broad pits and the central slab provide possibilities for the coexistence of the crystalline, fluid, and isotropic states of polymethylene chains, as will be discussed later.

**Fig. 7:**
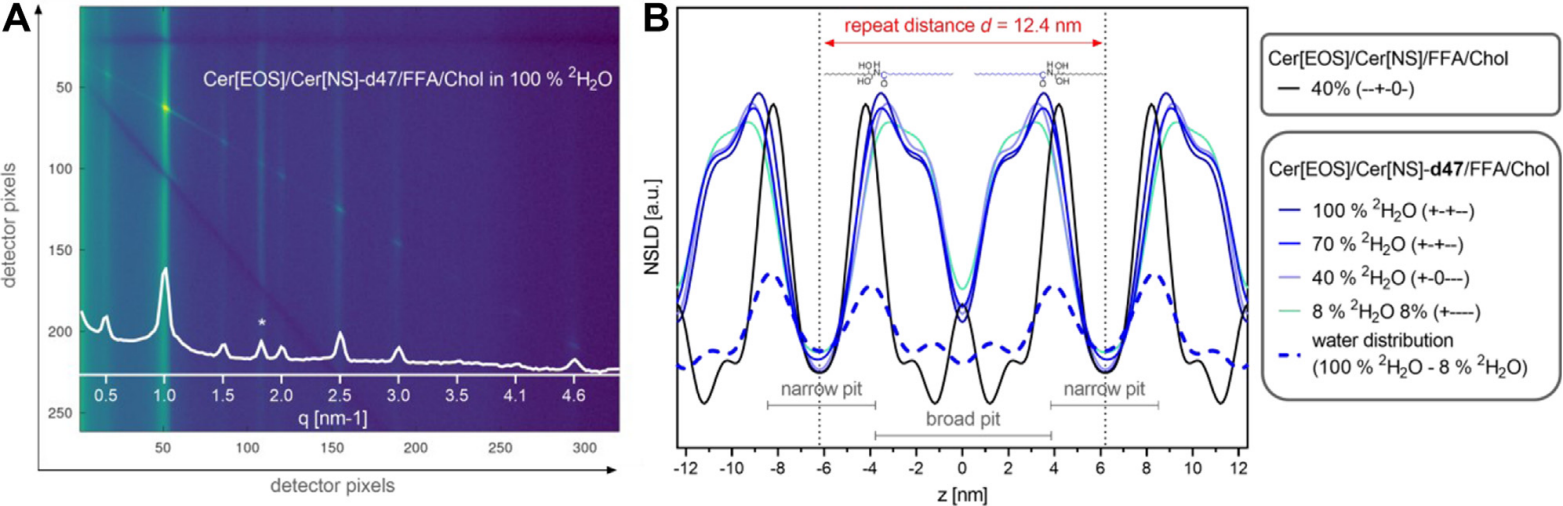
A: The ND 2D image and 1D pattern (white) of the Cer[EOS]/Cer[NS]-d_47_/FFA/Chol sample. An asterisk marks a peak of separated Chol. B: The relative NSLD profiles of the protonated Cer[EOS]/Cer[NS]/FFA/Chol and the deuterated Cer[EOS]/Cer[NS]-d_47_/FFA/Chol sample reconstructed using the indicated phase angles. The water distribution profile of the Cer[EOS]/Cer[NS]-d_47_/FFA/Chol sample was calculated as a difference between the NSLD at 100% and 8% ^2^H_2_O. The profile of the protonated sample is scaled and shifted for better clarity. The chemical structures represent the suggested arrangement of Cer[NS]-*d*_47_ molecules across the repeating unit (the polar groups are disproportionally enlarged for clarity). NSLD, neutron scattering length density.

The relative NSLD profiles were reconstructed for the Cer[EOS]/Cer[NS]-*d*_47_/FFA/Chol sample at 8, 40, and 100% ^2^H_2_O using all possible phase angle combinations of the first 5 nonzero *F*(*h*) (supplemental Figs. S9A–S11D). The most likely profile at 100% ^2^H_2_O (phase angles + - + - -) was selected because it was consistent with the profile of the protonated sample and provided a plausible water distribution obtained by subtracting the NSLD curve at 8% ^2^H_2_O (Fig. 7). It showed high NSLD peaks with maxima at a distance of 3.6 nm from the center of the unit, which had a different lineshape relative to the protonated sample. They carried additional shoulders in the direction toward the center of the unit. The shoulders are attributed to the *d*_47_-acyl chains of Cer[NS] molecules since the ^2^H isotope has the positive NSLD.

The narrow pit of the protonated sample seemed to be slightly narrower relative to the deuterated sample. This can be caused by multiple reasons: first of all, the refinement could be achieved involving *F*(*h*) of higher orders *h*. Furthermore, there can be small differences between individual samples due to their multiphase nature. The samples contained traces of phase-separated Chol. The fraction of phase-separated Chol and Chol embedded in the lamella can vary and therefore the nanostructure of the samples can be slightly different, however, the basic pattern of the NSLD distribution remains the same.

The arrangement of the *d*_47_-acyl chains in the relative NSLD profiles indicated that the broad pit is the acyl chain-enriched domain, while the narrow pit is the acyl chain-depleted domain. This nano-segregation ensures the coexistence of the crystalline state of acyl chains and the fluid state of the sphingosine chains. The slab at *z* = 0 nm with increased NSLD most likely represents the third state of the lipid moieties, the isotropic phase composed mainly of the linoleic acid-esterified ω-hydroxy terminal parts of the Cer[EOS] acyl chains. The analyzed ND data showed that the diffractometric results can be consistent with the molecular model of the skin lipid barrier based on the nano-segregated splayed chains of the Cer molecules.

## DISCUSSION

The current models of the SC lipid phase are characterized by a high degree of rigidity of the molecules forming ordered layers with high, long-range order (5, 6, 63). These insights were achieved by the great success of X-ray and neutron scattering techniques, which allowed determination of detailed information on layer thickness and the localization and distribution of specific molecular groups along the axis of the one-dimensional crystal. However, scattering techniques require high order of the molecular layers with long range order within the one-dimensional crystal and, thus, are less sensitive to dynamic aspects of the molecules under study. The coexistence of a liquid phase in SC models was deduced from small- and wide-angle X-ray diffraction studies (20, 64) and later confirmed by Fourier transform infrared and Raman spectroscopy as well as solid-state NMR results (17, 21, 65). These all contributed to the current sandwich model, which describes the LPP to be arranged in two broad crystalline layers that sandwich a narrow central fluid lipid domain (5, 20) also described elsewhere as isolated nanodroplets (17).

The current results are in agreement with the sandwich model but add important molecular details especially for the essential Cer[EOS] molecule. First, the linoleic acid at the tail end of the ultralong ω-hydroxyl chain of Cer[EOS] is isotropically mobile and completely disordered. This agrees with previous studies on Cer[EOS] with a physiologically less relevant oleic acid modification (17). Such isotropic mobility is not compatible with any highly ordered structure and requires a separate phase state which could be either quite extended because of the large cross-sectional area required for such a disordered chain or also lead to the formation of nanodroplets.

Second, the molecular disorder and high dynamics are also partially passed on to the C32:0 *N-*acyl chain of Cer[EOS]. Our ^2^H NMR results reveal that an astonishing 54% of the middle and 25% of the upper part of the acyl chain are also highly mobile, while only 27% of the middle and 53% of the upper part are rigid. This confirms that the C32:0 moiety has both dynamic and rigid character, which is very unique among all lipids of the SC. We suggest that the highly mobile parts of the C32:0 chain are particularly those at the chain end and the link to the linoleic moiety while a more rigid middle core exists. For the upper chain, we suggest that the more dynamic segments are comprised at the link to the sphingosine backbone, which is also shown to be quite dynamic (vide infra) and has also been shown for Cer[NS] in an SPP SC lipid model before (23).

Third, our data show that the terminal 4 carbons of the sphingosine chain are also mainly fluid as shown for the entire sphingosine chain of Cer[NS] before (23). The terminal part of the sphingosine chain is 95% fluid and 5% isotropic. No rigid phases have been observed in the ^2^H NMR spectra of this moiety.

**Fig. 8:**
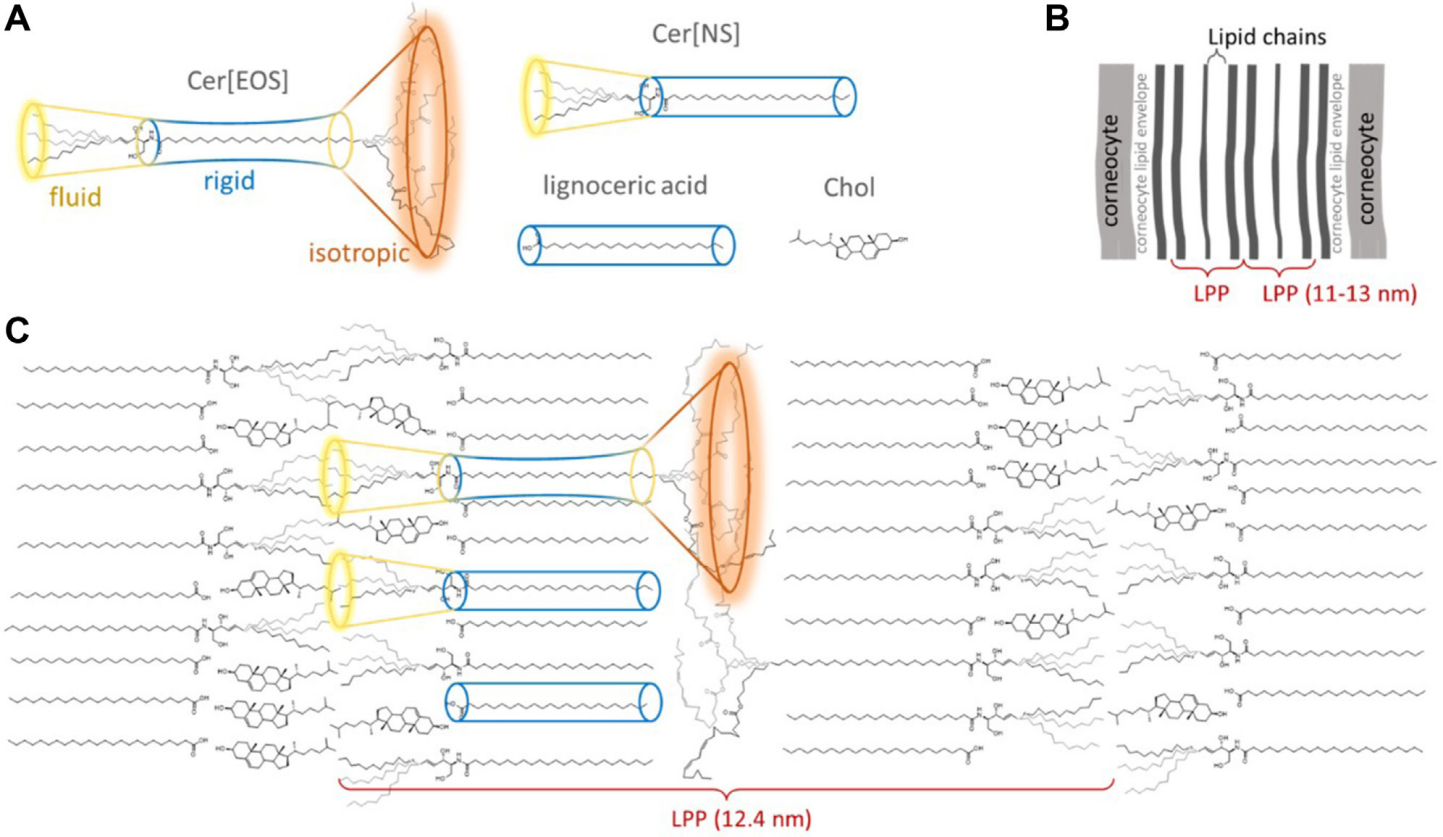
Cartoon representation of the basic unit of the LPP phase of Cer[EOS] containing SC model mixtures. The shape of the Cer[EOS] molecule is depicted as an unsymmetric double-sided cone with a relatively rigid center and increasing motional amplitudes toward either end of the molecule. In contrast, Cer[NS] acyl and FFA are essentially rigid (A). Panel B shows a scheme of the SC extracellular lipid ultrastructure (3,4). Panel C shows a cartoon of the proposed LPP unit structure. Here, Cer[NS] acyl and FFA are arranged with the central rigid part of Cer[EOS], whereas isotropic linoleates and fluid sphingoid base chains form two different slabs separating the rigid acyl layers here. Chol can associate with the fluid sphingosine or is segregated into crystalline domains. LPP, long periodicity phase; SC, *stratum corneum*.

How do these results extend the sandwich model of the LPP in Cer[EOS]-containing models of the SC? All models of the SC arrange the lipid molecules in the densest fashion such that there are no voids/defects within the tightly packed lipid arrangement. Any void volume that occurs due to packing defects has to be filled by molecular motions of increased amplitude. Lower molecular order increases the cross-sectional area of the molecule, leading to a shape that is characterized by the smallest cross-sectional area in the middle and a much larger area toward either end of the molecule as depicted in a cartoon in Figure 8. As Cer[EOS] most likely assumes an extended conformation (17) the molecular shape of Cer[EOS] may resemble an unsymmetric double sided cone rather than a cylinder of constant cross-sectional area, which characterizes the shape of FFA molecules in the SC. The particular shape of Cer[EOS] allows for the large amplitude motions on either end of the Cer[EOS] molecule. As also shown in this work, the other components of the LPP mixture (Cer[NS], FFA, and Chol) are essentially rigid and do not show a similarly dynamic behavior. This suggests that these molecules are predominantly arranged with the rigid core of the central C32:0 chain of Cer[EOS] as depicted in Figure 8. As some of the Chol was also found to be fluid, it is conceivable that it associates with the fluid sphingosine as also suggested before (23, 24, 39, 66). Likely, the rigid Chol is phase separated from the fluid lipids (24). Also, our SAXS data showed some crystalline Chol.

MD simulations have also been conducted on different models of the LPP-containing Cer[EOS] species (22). In the work by Wang and Klauda, model 2 featured a slab in the center of the structure composed of highly mobile lipids (Cer[NS], Cer[EOS], and FFA). Only order parameters for the C24:0 chains were calculated from the simulation and are reported to be ≤0.2. Clearly, such a scenario is not supported by our data as the acyl chains of Cer[NS] and LA remain in a predominantly rigid state. However, the order parameters for Cer[EOS] calculated from the MD simulation (22) do show a plateau with high-order parameters between C10 and C25, which drops to very low values for C2-C9 and C26-C32. This is in good agreement with our data. For the linoleate, the MD order parameters are not isotropic but relatively low throughout the C18:2 chain (≤0.1).

What are the biological implications of these results? The SC lipids are our first line of defense against various environmental threats; thus, it is not surprising that they have been described as highly rigid and tightly packed for decades (5). Yet, rigid and tightly packed lipid multilayers seem incompatible with numerous skin properties and actions. First, skin is remarkably elastic; it swells upon hydration, it can be stretched and bounces back into place, and the lipids should adapt to such movements without breaks that would compromise the permeability barrier (67). The barrier lipids also share the extracellular space with enzymes, inhibitors, signaling molecules, and antimicrobial peptides, all of which would require less rigid, more dynamic environments for their action (68). Fluid chains are also needed during the barrier assembly to squeeze into and fill narrow extracellular spaces without significant packing defects and, hence, permeability (69). Here, we propose an LPP model of alternating rigid (FFA and Cer acyl chains) and dynamic layers (sphingosine-rich layer and isotropic linoleate slab; Fig. 8) that would satisfy this need for simultaneous rigidity and fluidity in the skin lipids.

This model is consistent with the SC lipid ultrastructure as seen by electron microscopy with RuO_4_ staining (3) (we propose that the broad lucent bands comprise the rigid acyl chains and the narrow lucent band is rich in sphingoid base chains) but also with the model proposed using computer simulations and cryo-electron microscopy on near-native skin (70) (the lipid arrangement is essentially the same, only the isotropic slab is more pronounced in our model). The localization of the isotropic linoleates in a layer-like fashion between the rigid acyl chains is consistent with the thin electron-dense band in transmission electron microscopy with RuO_4_ staining in both mature SC lipids and, importantly, in the precursor lipid lamellae in lamellar bodies (3). In mice with deficient-PNPLA1, a transacylase required for the linoleate attachment to form Cer[EOS], this band is absent (71). The isotropic linoleate slab in the hydrophobic core of both precursor and mature lipid lamellae resembles a lipid droplet lens (72), which makes sense as lipid droplet triglycerides are a source of linoleate for Cer[EOS] synthesis (73). This lipid droplet lens-like isotropic core of the lipid lamellae would alter their mechanical properties (e.g., triolein induces extraordinary conformational dynamics in phospholipid membranes, which are then able to squeeze through narrow passages between neighboring structures) (74). The proposed model also offers a relatively straightforward transition from lamellar body bilayer structures to the mature LPP: upon enzymatic processing of the lipid precursors and acidification, the Cer-rich bilayers would become unstable, and the strain will be reduced by sphingoid base chain flip (along with most Chol molecules), forming the LPP unit.

Thus, our model of the skin barrier lipids with rigid layers separated with two different dynamic “fillings” i) agrees well with ultrastructural data, ii) satisfies the need for simultaneous lipid rigidity (to ensure low permeability) and fluidity (to ensure elasticity, accommodate enzymes or antimicrobial peptides), and iii) offers a straightforward way to remodel the lamellar body lipids into the final lipid barrier.

## Data availability

All data are available upon request from D. Huster, Institute of Medical Physics and Biophysics, University of Leipzig (daniel.huster@medizin.uni-leipzig.de) and K. Vávrová, Faculty of Pharmacy, Charles University, Hradec Králové (Kateřina Vávrová (vavrovak@faf.cuni.cz).

## Supplemental data

This article contains supplemental data (25, 31, 34, 35, 61, 62, 75, 76, 77, 78).

## Conflict of interest

The authors declare that they have no conflicts of interest with the contents of this article.

## References

[1] Scheuplein R.J., Blank I.H. (1971). Permeability of the skin. Physiol. Rev..

[2] Iwai I., Han H., den H.L., Svensson S., Ofverstedt L.G., Anwar J. (2012). The human skin barrier is organized as stacked bilayers of fully extended ceramides with cholesterol molecules associated with the ceramide sphingoid moiety. J. Invest. Dermatol..

[3] Madison K.C., Swartzendruber D.C., Wertz P.W., Downing D.T. (1987). Presence of intact intercellular lipid lamellae in the upper layers of the stratum corneum. J. Invest. Dermatol..

[4] Swartzendruber D.C., Wertz P.W., Kitko D.J., Madison K.C., Downing D.T. (1989). Molecular models of the intercellular lipid lamellae in mammalian stratum corneum. J. Invest. Dermatol..

[5] van Smeden J., Janssens M., Gooris G.S., Bouwstra J.A. (2014). The important role of stratum corneum lipids for the cutaneous barrier function. Biochim. Biophys. Acta.

[6] Bouwstra J.A., Ponec M. (2006). The skin barrier in healthy and diseased state. Biochim. Biophys. Acta.

[7] White S.H., Mirejovsky D., King G.I. (1988). Structure of lamellar lipid domains and corneocyte envelopes of murine stratum corneum. An X-ray diffraction study. Biochemistry.

[8] Kessner D., Ruettinger A., Kiselev M.A., Wartewig S., Neubert R.H. (2008). Properties of ceramides and their impact on the stratum corneum structure. Part 2: stratum corneum lipid model systems. Skin Pharmacol. Physiol..

[9] Hou S.Y., Mitra A.K., White S.H., Menon G.K., Ghadially R., Elias P.M. (1991). Membrane structures in normal and essential fatty acid-deficient stratum corneum: characterization by ruthenium tetroxide staining and x-ray diffraction. J. Invest. Dermatol..

[10] Bouwstra J.A., Gooris G.S., van der Spek J.A., Bras W. (1991). Structural investigations of human stratum corneum by small-angle X-ray scattering. J. Invest. Dermatol..

[11] Mendelsohn R., Moore D.J. (2000). Infrared determination of conformational order and phase behavior in ceramides and stratum corneum models. Met. Enzymol..

[12] Pham Q.D., Topgaard D., Sparr E. (2017). Tracking solvents in the skin through atomically resolved measurements of molecular mobility in intact stratum corneum. Proc. Natl. Acad. Sci. U. S. A..

[13] Stahlberg S., Skolova B., Madhu P.K., Vogel A., Vavrova K., Huster D. (2015). Probing the role of the ceramide acyl chain length and sphingosine unsaturation in model skin barrier lipid mixtures by 2H solid-state NMR spectroscopy. Langmuir.

[14] Kitson N., Thewalt J., Lafleur M., Bloom M. (1994). A model membrane approach to the epidermal permeability barrier. Biochemistry.

[15] Brief E., Kwak S., Cheng J.T., Kitson N., Thewalt J., Lafleur M. (2009). Phase behavior of an equimolar mixture of N-palmitoyl-D-erythro-sphingosine, cholesterol, and palmitic acid, a mixture with optimized hydrophobic matching. Langmuir.

[16] Engelbrecht T.N., Schröter A., Hauss T., Deme B., Scheidt H.A., Huster D. (2012). The impact of ceramides NP and AP on the nanostructure of stratum corneum lipid bilayer. Part I: neutron diffraction and H-2 NMR studies on multilamellar models based on ceramides with symmetric alkyl chain length distribution. Soft Matter.

[17] Paz Ramos A., Gooris G., Bouwstra J., Lafleur M. (2018). Evidence of hydrocarbon nanodrops in highly ordered stratum corneum model membranes. J. Lipid Res..

[18] Eichner A., Stahlberg S., Sonnenberger S., Lange S., Dobner B., Ostermann A. (2017). Influence of the penetration enhancer isopropyl myristate on stratum corneum lipid model membranes revealed by neutron diffraction and 2H NMR experiments. Biochim. Biophys. Acta.

[19] Paz Ramos A., Lafleur M. (2015). Chain length of free fatty acids influences the phase behavior of stratum corneum model membranes. Langmuir.

[20] Bouwstra J.A., Gooris G.S., Dubbelaar F.E., Ponec M. (2001). Phase behavior of lipid mixtures based on human ceramides: coexistence of crystalline and liquid phases. J. Lipid Res..

[21] Pham Q.D., Mojumdar E.H., Gooris G.S., Bouwstra J.A., Sparr E., Topgaard D. (2018). Solid and fluid segments within the same molecule of stratum corneum ceramide lipid. Q. Rev. Biophys..

[22] Wang E., Klauda J.B. (2019). Molecular structure of the long periodicity phase in the stratum corneum. J. Am. Chem. Soc..

[23] Engberg O., Kovacik A., Pullmannová P., Juhašcik M., Opálka L., Huster D. (2020). The sphingosine and acyl chains of ceramide [NS] show very different structure and dynamics challenging our understanding of the skin barrier. Angew. Chem. Int. Ed..

[24] Fandrei F., Engberg O., Opalka L., Jancalkova P., Pullmannova P., Steinhart M. (2022). Cholesterol sulfate fluidizes the sterol fraction of the stratum corneum lipid phase and increases its permeability. J. Lipid Res..

[25] Opalka L., Kovacik A., Sochorova M., Roh J., Kunes J., Lenco J. (2015). Scalable synthesis of human ultralong chain ceramides. Org. Lett..

[26] Kovacik A., Vogel A., Adler J., Pullmannova P., Vavrova K., Huster D. (2018). Probing the role of ceramide hydroxylation in skin barrier lipid models by 2H solid-state NMR spectroscopy and X-ray powder diffraction. Biochim. Biophys. Acta.

[27] Groen D., Gooris G.S., Bouwstra J.A. (2010). Model membranes prepared with ceramide EOS, cholesterol and free fatty acids form a unique lamellar phase. Langmuir.

[28] Davis J.H., Jeffrey K.R., Bloom M., Valic M.I., Higgs T.P. (1976). Quadrupolar echo deuteron magnetic resonance spectroscopy in ordered hydrocarbon chains. Chem. Phys. Lett..

[29] Huster D., Arnold K., Gawrisch K. (1998). Influence of docosahexaenoic acid and cholesterol on lateral lipid organization in phospholipid membranes. Biochemistry.

[30] Zhou Z., Kümmerle R., Qiu X., Redwine D., Cong R., Taha A. (2007). A new decoupling method for accurate quantification of polyethylene copolymer composition and triad sequence distribution with 13C NMR. J. Magn. Reson..

[31] Smith A.A. (2017). INFOS: spectrum fitting software for NMR analysis. J. Biomol. NMR..

[32] Smith A.A., Ernst M., Meier B.H. (2018). Optimized "detectors" for dynamics analysis in solid-state NMR. J. Chem. Phys..

[33] Gonthier J., Barrett M.A., Aguettaz O., Baudoin S., Bourgeat-Lami E., Deme B. (2019). BerILL: the ultimate humidity chamber for neutron scattering. J. Neutron Res..

[34] Kucerka N., Nieh M.P., Pencer J., Sachs J.N., Katsaras J. (2009). What determines the thickness of a biological membrane. Gen. Physiol. Biophys..

[35] Kirschner D.A., Sidman R.L. (1976). X-ray diffraction study of myelin structure in immature and mutant mice. Biochim. Biophys. Acta.

[36] Pullmannova P., Curikova-Kindlova B.A., Ondrejcekova V., Kovacik A., Dvorakova K., Dulanska L. (2023). Polymorphism, nanostructures, and barrier properties of ceramide-based lipid films. ACS Omega.

[37] Kiuchi F., Nakamura N., Saitoh M., Komagome K., Hiramatsu H., Takimoto N. (1997). Synthesis and nematocidal activity of aralkyl- and aralkenylamides related to piperamide on second-stage larvae of Toxocara canis. Chem. Pharm. Bull..

[38] Dhimitruka H., SantaLucia J. (2006). Investigation of the Yamaguchi esterification mechanism. Synthesis of a Lux-S enzyme inhibitor using an improved esterification method. Org. Lett..

[39] Pullmannova P., Ermakova E., Kovacik A., Opalka L., Maixner J., Zbytovska J. (2019). Long and very long lamellar phases in model stratum corneum lipid membranes. J. Lipid Res..

[40] Marsh D. (2012). Lateral order in gel, subgel and crystalline phases of lipid membranes: wide-angle X-ray scattering. Chem. Phys. Lipids.

[41] Taylor M.G., Akiyama T., Smith I.C.P. (1981). The molecular dynamics of cholesterol in bilayer membranes: a deuterium NMR study. Chem. Phys. Lipids.

[42] Dufourc E.J., Parish E.J., Chitrakorn S., Smith C.P. (1984). Structural and dynamical details of cholesterol-lipid interaction as revealed by deuterium NMR. Biochemistry.

[43] Polozov I.V., Gawrisch K. (2006). Characterization of the liquid-ordered state by proton MAS NMR. Biophys. J..

[44] Bunge A., Müller P., Stöckl M., Herrmann A., Huster D. (2008). Characterization of the ternary mixture of sphingomyelin, POPC, and cholesterol: support for an inhomogeneous lipid distribution at high temperatures. Biophys. J..

[45] Bartels T., Lankalapalli R.S., Bittman R., Beyer K., Brown M.F. (2008). Raftlike mixtures of sphingomyelin and cholesterol investigated by solid-state ^2^H NMR spectroscopy. J. Am. Chem. Soc..

[46] Veatch S.L., Soubias O., Keller S.L., Gawrisch K. (2007). Critical fluctuations in domain-forming lipid mixtures. Proc. Natl. Acad. Sci. U. S. A..

[47] Stahlberg S., Lange S., Dobner B., Huster D. (2016). Probing the role of ceramide headgroup polarity in short-chain model skin barrier lipid mixtures by 2H solid-state NMR spectroscopy. Langmuir.

[48] Kwak S., Brief E., Langlais D., Kitson N., Lafleur M., Thewalt J. (2012). Ethanol perturbs lipid organization in models of stratum corneum membranes: an investigation combining differential scanning calorimetry, infrared and 2H NMR spectroscopy. Biochim. Biophys. Acta.

[49] Castro B.M., Prieto M., Silva L.C. (2014). Ceramide: a simple sphingolipid with unique biophysical properties. Prog. Lipid Res..

[50] Mouts A., Vattulainen E., Matsufuji T., Kinoshita M., Matsumori N., Slotte J.P. (2018). On the importance of the C(1)-OH and C(3)-OH functional groups of the long-chain base of ceramide for interlipid interaction and lateral segregation into ceramide-rich domains. Langmuir.

[51] Fanani M.L., Maggio B. (2017). The many faces (and phases) of ceramide and sphingomyelin II - binary mixtures. Biophys. Rev..

[52] Alonso A., Goni F.M. (2018). The physical properties of ceramides in membranes. Annu. Rev. Biophys..

[53] Vavrova K., Hrabalek A., Mac-Mary S., Humbert P., Muret P. (2007). Ceramide analogue 14S24 selectively recovers perturbed human skin barrier. Br. J. Dermatol..

[54] Pham Q.D., Carlstrom G., Lafon O., Sparr E., Topgaard D. (2020). Quantification of the amount of mobile components in intact stratum corneum with natural-abundance 13C solid-state NMR. Phys. Chem. Chem. Phys..

[55] Huster D., Arnold K., Gawrisch K. (1999). Investigation of lipid organization in biological membranes by two-dimensional nuclear Overhauser enhancement spectroscopy. J. Phys. Chem. B..

[56] Davis J.H., Auger M., Hodges R.S. (1995). High resolution ^1^H nuclear magnetic resonance of a transmembrane peptide. Biophys. J..

[57] Forbes J., Husted C., Oldfield E. (1988). High-field, high-resolution proton "magic-angle" sample-spinning nuclear magnetic resonance spectroscopic studies of gel and liquid crystalline lipid bilayers and the effects of cholesterol. J. Am. Chem. Soc..

[58] Nowacka A., Bongartz N.A., Ollila O.H., Nylander T., Topgaard D. (2013). Signal intensities in (1)H-(1)(3)C CP and INEPT MAS NMR of liquid crystals. J. Magn. Reson..

[59] Smith A.A., Ernst M., Meier B.H. (2017). Because the light is better here: correlation-time analysis by NMR spectroscopy. Angew. Chem. Int. Ed..

[60] Smith A.A., Vogel A., Engberg O., Hildebrand P.W., Huster D. (2022). A method to construct the dynamic landscape of a bio-membrane with experiment and simulation. Nat. Commun..

[61] Franks N.P., Lieb W.R. (1979). The structure of lipid bilayers and the effects of general anaesthetics. An x-ray and neutron diffraction study. J. Mol. Biol..

[62] Mojumdar E.H., Gooris G.S., Barlow D.J., Lawrence M.J., Deme B., Bouwstra J.A. (2015). Skin lipids: localization of ceramide and fatty acid in the unit cell of the long periodicity phase. Biophys. J..

[63] Shamaprasad P., Frame C.O., Moore T.C., Yang A., Iacovella C.R., Bouwstra J.A. (2022). Using molecular simulation to understand the skin barrier. Prog. Lipid Res..

[64] Bouwstra J.A., Gooris G.S., Dubbelaar F.E., Ponec M. (2002). Phase behavior of stratum corneum lipid mixtures based on human ceramides: the role of natural and synthetic ceramide 1. J. Invest. Dermatol..

[65] Janssens M., Gooris G.S., Bouwstra J.A. (2009). Infrared spectroscopy studies of mixtures prepared with synthetic ceramides varying in head group architecture: coexistence of liquid and crystalline phases. Biochim. Biophys. Acta.

[66] McIntosh T.J. (2003). Organization of skin stratum corneum extracellular lamellae: diffraction evidence for asymmetric distribution of cholesterol. Biophys. J..

[67] Forslind B. (1994). A domain mosaic model of the skin barrier. Acta Derm. Venereol..

[68] Elias P.M. (2012). Structure and function of the stratum corneum extracellular matrix. J. Invest. Dermatol..

[69] Sagrafena I., Paraskevopoulos G., Pullmannova P., Opalka L., Novackova A., Lourantou O. (2022). Assembly of human stratum corneum lipids in vitro: fluidity matters. J. Invest. Dermatol..

[70] Lundborg M., Narangifard A., Wennberg C.L., Lindahl E., Daneholt B., Norlen L. (2018). Human skin barrier structure and function analyzed by cryo-EM and molecular dynamics simulation. J. Struct. Biol..

[71] Opalka L., Meyer J.M., Ondrejcekova V., Svatosova L., Radner F.P.W., Vavrova K. (2022). omega-O-Acylceramides but not omega-hydroxy ceramides are required for healthy lamellar phase architecture of skin barrier lipids. J. Lipid Res..

[72] Olzmann J.A., Carvalho P. (2019). Dynamics and functions of lipid droplets. Nat. Rev. Mol. Cell Biol..

[73] Breiden B., Sandhoff K. (2014). The role of sphingolipid metabolism in cutaneous permeabilitybarrier formation. Biochim. Biophys. Acta.

[74] Pakkanen K.I., Duelund L., Qvortrup K., Pedersen J.S., Ipsen J.H. (2011). Mechanics and dynamics of triglyceride-phospholipid model membranes: implications for cellular properties and function. Biochim. Biophys. Acta.

[75] Groen D., Gooris G.S., Barlow D.J., Lawrence M.J., van Mechelen J.B., Deme B. (2011). Disposition of ceramide in model lipid membranes determined by neutron diffraction. Biophys J.

[76] Nagle J.F., Akabori K., Treece B.W., Tristram-Nagle S. (2016). Determination of mosaicity in oriented stacks of lipid bilayers. Soft Matter.

[77] Coleman T.F., Li Y. (1996). An interior trust region approach for nonlinear minimization subject to bounds. SIAM J Optim.

[78] Coleman T.F., Li Y. (1996). A reflective Newton method for minimizing a quadratic function subject to bounds on some of the variables. SIAM J Optim.

