# Supplementary material for: The intriguing molecular dynamics of Cer[EOS] in rigid skin barrier lipid layers requires improvement of the model
Source: J Lipid Res. 2023 Mar 21;64(5):100356. doi: 10.1016/j.jlr.2023.100356 (PMC10154977; doi:10.1016/j.jlr.2023.100356)
Supplement: Supporting information [file mmc1.docx]

**Supporting Information**

**The Intriguing Molecular Dynamics of Cer[EOS] in Rigid Skin Barrier Lipid Layers Requires Improvement of the Model**

Ferdinand Fandrei,^1,#^ Tomáš Havrišák,^2,#^ Lukáš Opálka,^2,#^ Oskar Engberg,^1^ Albert A. Smith,^1^ Petra Pullmannová,^2^ Norbert Kučerka^3^, Veronika Ondrejčeková^2^, Bruno Demé^4^, Lucie Nováková,^5^ Miloš Steinhart,^6^ Kateřina Vávrová,^2^ Daniel Huster^1,^*

^1^ Institute of Medical Physics and Biophysics, University of Leipzig, Härtelstr. 16-18, 04275 Leipzig, Germany

^2^ Skin Barrier Research Group, Faculty of Pharmacy, Charles University, Akademika Heyrovského 1203, 50005 Hradec Králové, Czech Republic

^3^ Faculty of Pharmacy, Comenius University in Bratislava, Odbojárov 10, 832 32 Bratislava, Slovakia

^4^ Institut Laue-Langevin, 71 avenue des Martyrs, CS 20156, 38042 Grenoble, CEDEX 9, France

^5^Department of Analytical Chemistry, Faculty of Pharmacy, Charles University, Akademika Heyrovského 1203, 50005 Hradec Králové, Czech Republic

^6^Institute of Macromolecular Chemistry, Czech Academy of Science in Prague, Heyrovského nám. 2,

162 06 Prague, Czech Republic

^#^ These authors contributed equally

# Supporting Materials and Methods

## Synthesis

### Dodecane-1,12-diyl-*d*_24_ diacetate **(1)**

1 g (2.85 mmol) of d_24_-1,12-dibromododecane and 1 g (12.1 mmol) of sodium acetate were dissolved in 60 mL of DMF. The reaction mixture was stirred at 100 °C for 3 hours. Then 50 mL of toluene was added and solvents were evaporated. This procedure was repeated one more time to remove all DMF. Crude product was purified using column chromatography (Hex/EtOAc 30:1) to provide 680 mg (77 %) of **(1)** as a white solid. **^1^H NMR** (500 MHz, CDCl_3_) δ 2.05 (s, 6H) ppm. **^13^C NMR** (126 MHz, CDCl_3_) δ 171.24, 20.99 ppm. **IR** 2193, 2089, 1729, 1372, 1270, 1225 cm^-1^. **Melting point** 32 – 34 °C. **HRMS** C_16_H_6_D_24_O_4_ [M+H]^+^ calculated 311.3723; found 311.3729; [M+Na]^+^ calculated 333.3543; found 333.3549.

### Dodecane-*d*_24_-1,12-diol **(2)**

677 mg (2.18 mmol) of **(1)** was dissolved in 40 mL of MeOH and 360 mg of DOWEX 50 (53 % w/w) was added. The reaction mixture was stirred over night at reflux. After cooling down, DOWEX was filtered off, solvent was evaporated and the crude product was purified using column chromatography (Hex/EtOAc 2:1) to provide 463 mg (94 %) of **(2)** as a white solid. **^1^H NMR** (600 MHz, MeOD) δ 4.82 (s, 2H) ppm. **^13^C NMR** (151 MHz, MeOD) no signals. **IR** 3403, 3337, 2199, 2089, 1745 cm^-1^. **Melting point** 77 – 78 °C. **HRMS** C_12_H_2_D_24_O_2_ [M+H]^+^ calculated 227.3512; found 227.3519; [M+Na]^+^ calculated 249.3331; found 249.3337.

### 12-bromododecan-1,1,2,2,3,3,4,4,5,5,6,6,7,7,8,8,9,9,10,10,11,11,12,12-*d*_24_-1-ol **(3)**

457 mg (2.02 mmol) of **(2)** was dissolved in 15 mL of toluene and heated to reflux. 0.3 mL of 48 % HBr (2.62 mmol) was added dropwise and the reaction mixture was stirred at reflux for 90 min. After cooling down, the reaction mixture was diluted with Et_2_O and extracted with 1M NaOH and brine. Organic phase was evaporated and the crude product was purified using column chromatography (Hex/EtOAc 15:1). The unreacted starting material was again dissolved in 10 mL of toluene and the process was repeated with 0.1 mL of 48 % HBr. After both reaction cycles, 465 mg (80 %) of **(3)** was obtained as a yellow oil. **^1^H NMR** (500 MHz, CDCl_3_) no signals. **^13^C NMR** (126 MHz, CDCl_3_) no signals. **IR** 3391, 3325, 2195, 2090, 1745 cm^-1^.

### 2-[(12-bromododecyl-1,1,2,2,3,3,4,4,5,5,6,6,7,7,8,8,9,9,10,10,11,11,12,12-*d*_24_)oxy]tetrahydro-2*H*-pyran **(4)**

435 mg (1.5 mmol) of **(3)** was mixed with 152 mg (0.6 mmol) of pyridinium *p*-toluenesulfonate and dried at high vacuum. 7 mL of dry CHCl_3_ was added followed by an addition of 280 µL (3 mmol) of 3,4-dihydro-2*H*-pyran. The reaction mixture was stirred for 3 hours at reflux under an Ar atmosphere. Reaction was quenched by an addition of 15 mL of water and it was extracted with 3 × 15 mL of CHCl_3_. Mixed organic phase was evaporated and the crude product was purified using column chromatography (Hex/EtOAc 50:1) to provide 524 mg (94 %) of **(4)** as a yellow oil. **^1^H NMR** (500 MHz, CDCl_3_) δ 4.66 – 4.50 (m, 1H), 3.97 – 3.81 (m, 1H), 3.58 – 3.44 (m, 1H), 1.93 – 1.77 (m, 1H), 1.78 – 1.66 (m, 1H), 1.65 – 1.46 (m, 4H) ppm. **^13^C NMR** (126 MHz, CDCl_3_) δ 98.80, 62.35, 30.79, 25.50, 19.71 ppm. **IR** 2941, 2197, 2096 cm^-1^. **HRMS** C_17_H_9_D_24_BrO_2_ [M+Na]^+^ calculated 395.3063; found 395.3065.

### 12-[(tetrahydro-2*H*-pyran-2-yl)oxy]dodecyl-1,1,2,2,3,3,4,4,5,5,6,6,7,7,8,8,9,9,10,10,11,11,12,12-*d*_24_ acetate **(5)**

512 mg (1.4 mmol) of **(4)** was mixed with 225 mg (2.8 mmol) of sodium acetate and dissolved in 12 mL of DMF. The reaction mixture was stirred at 100 °C for 3 hours. Then 10 mL of toluene was added and solvents were evaporated. This procedure was repeated one more time to remove all DMF. Crude product was purified using column chromatography (Hex/EtOAc 30:1) to provide 444 mg (91 %) of **(5)** as a colorless oil. **^1^H NMR** (600 MHz, CDCl_3_) δ 4.59 – 4.51 (m, 1H), 3.92 – 3.79 (m, 1H), 3.53 – 3.44 (m, 1H), 2.02 (s, 3H), 1.88 – 1.76 (m, 1H), 1.73 – 1.65 (m, 1H), 1.61 – 1.46 (m, 4H) ppm. **^13^C NMR** (151 MHz, CDCl_3_) δ 171.35, 98.90, 62.44, 30.89, 25.60, 21.10, 19.81 ppm. **IR** 2941, 2199, 2097, 1739 cm^-1^. **HRMS** C_19_H_12_D_24_O_4_ [M+Na]^+^ calculated 375.4012; found 375.4022.

### 12-[(tetrahydro-2*H*-pyran-2-yl)oxy]dodecan-1,1,2,2,3,3,4,4,5,5,6,6,7,7,8,8,9,9,10,10,11,11,12,12-*d*_24_-1-ol **(6)**

431 mg (1.2 mmol) of **(5)** was dried at high vacuum, dissolved in 9 mL of dry THF and cooled down to 0 °C in an ice bath. 3 mL (3 mmol) of 1M LiAlH_4_ solution in THF was then added dropwise, reaction mixture was removed from an ice bath and it was stirred over night at room temperature under an Ar atmosphere. The reaction was quenched by a careful addition of 10 mL of water and it was extracted with 3 × 15 mL of CHCl_3_. Organic phase was evaporated and the crude product was purified using column chromatography (Hex/EtOAc 6:1) to provide 361 mg (95 %) of **(6)** as a yellowish oil. **^1^H NMR** (600 MHz, CDCl_3_) δ 4.57 – 4.53 (m, 1H), 3.90 – 3.81 (m, 1H), 3.51 – 3.44 (m, 1H), 1.86 – 1.76 (m, 1H), 1.74 – 1.65 (m, 1H), 1.62 – 1.43 (m, 4H) ppm. **^13^C NMR** (151 MHz, CDCl_3_) δ 98.90, 62.43, 30.88, 25.59, 19.79 ppm. **IR** 3356, 2941, 2197, 2094 cm^-1^. **HRMS** C_17_H_10_D_24_O_3_ [M+Na]^+^ calculated 333.3907; found 333.3914.

### 12-[(tetrahydro-2*H*-pyran-2-yl)oxy]dodecanal-1,2,2,3,3,4,4,5,5,6,6,7,7,8,8,9,9,10,10,11,11,12,12-*d*_23_ **(7)**

185 µL (2.1 mmol) of oxalyl chloride was dissolved in 10 mL of dry DCM in an oven-dried flask under an Ar atmosphere. The flask was cooled down to -60 °C and 350 µL (4.6 mmol) of dry DMSO was added in 5 mL of dry DCM. After 10 min of stirring, 355 mg (1.2 mmol) of **(6)** in 5 mL of dry DCM was added and the reaction mixture was stirred at -60 °C for 90 min. After 90 min, 640 µL (4.6 mmol) of dry triethylamine was added, reaction mixture was slowly allowed to reach room temperature and it was further left stirring at room temperature over night under an Ar atmosphere. The reaction was quenched by an addition of brine and it was extracted with 3 × 15 mL of CHCl_3_. The mixed organic phase was evaporated and the crude product was purified using column chromatography (Hex/EtOAc 15:1) to provide 238 mg (68 %) of **(7)** as a colorless oil. **^1^H NMR** (500 MHz, CDCl_3_) δ 4.60 – 4.55 (m, 1H), 3.92 – 3.84 (m, 1H), 3.55 – 3.45 (m, 1H), 1.89 – 1.77 (m, 1H), 1.77 – 1.66 (m, 1H), 1.63 – 1.44 (m, 4H) ppm. **^13^C NMR** (126 MHz, CDCl_3_) δ 98.80, 62.34, 30.79, 25.49, 19.71 ppm. **IR** 2941, 2198, 2095, 1712 cm^-1^. **HRMS** C_17_H_9_D_23_O_3_ [M+Na]^+^ calculated 330.3687; found 330.3694.

### (3-carboxypropyl-1,1,2,2,3,3-*d*_6_)triphenylphosphonium bromide **(8)**

1416 mg (4.1 mmol) of triphenylphosphine was mixed with 401 mg (4.3 mmol) of d_6_-γ-butyrolactone and heated without a solvent at 190 °C for 3 hours until the melt solidified. After cooling, the reaction mixture was dissolved in minimal volume of CHCl_3_ and it was precipitated in pre-cooled Et_2_O. The precipitated product was filtered off and dried to yield 1470 mg (82 %) of **(8)** as a white solid. **^1^H NMR** (600 MHz, MeOD) δ 7.91 – 7.85 (m, 3H), 7.85 – 7.79 (m, 6H), 7.78 – 7.71 (m, 6H) ppm. **^13^C NMR** (151 MHz, MeOD) δ 174.37, 135.04, 133.56, 133.49, 130.30, 130.21, 118.69, 118.13 ppm. **IR** 3011, 2179, 2112, 1716, 1588, 1441, 1361, 1174, 1116, 1107 cm^-1^. **Melting point** 241 – 244 °C.

### (*E/Z*)-16-[(tetrahydro-2*H*-pyran-2-yl)oxy]hexadec-4-enoic-2,2,3,3,4,5,6,6,7,7,8,8,9,9,10,10,11,11,12,12,13,13,14,14,15,15,16,16-*d*_28_ acid **(9)**

1040 mg (2.4 mmol) of **(8)** was dried at high vacuum, then suspended in 5 mL of dry THF under an Ar atmosphere and cooled down to -30 °C. 5.5 mL (5.5 mmol) of 1M NaHMDS in THF was added dropwise and the mixture was stirred for additional 60 min. 227 mg (0.7 mmol) of **(7)** in 5 mL of THF was added and the cooling was continued for 30 min. The reaction was stirred over night at room temperature under an Ar atmosphere. Reaction was quenched by an addition of 20 mL of saturated NH_4_Cl, it was acidified with concentrated HCl to pH 1 and extracted with 3 × 50 mL of CHCl_3_. Mixed organic phase was evaporated and the crude product was purified using column chromatography (Hex/EtOAc 15:1 with 0.5 % AcOH) to provide 180 mg (63 %) of **(9)** as a white oil. **^1^H NMR** (600 MHz, CDCl_3_) δ 4.59 – 4.55 (m, 1H), 3.90 – 3.83 (m, 1H), 3.52 – 3.45 (m, 1H), 1.87 – 1.76 (m, 1H), 1.74 – 1.65 (m, 1H), 1.63 – 1.44 (m, 4H) ppm. **^13^C NMR** (151 MHz, CDCl_3_) δ 178.67, 126.90, 98.90, 62.43, 30.86, 25.57, 19.77 ppm. **IR** 2941, 2197, 2095, 1737, 1709 cm^-1^. **HRMS** C_21_H_10_D_28_O_4_ [M+Na]^+^ calculated 405.4420; found 405.4418.

### 16-[(tetrahydro-2*H*-pyran-2-yl)oxy]hexadecanoic-2,2,3,3,4,4,5,5,6,6,7,7,8,8,9,9,10,10,11,11,12,12,13,13,14,14,15,15,16,16-*d*_30_ acid **(10)**

160 mg (0.4 mmol) of (9) was dissolved in 20 mL of EtOAc in double-neck flask and 13 mg (1.2 µmol) of 10 % Pd/C was added. The atmosphere was replaced with D_2_ from a balloon. The reaxtion mixture was vigorously stirred over night at room temperature. Pd was filtered off, solvent was removed by evaporation and the crude product was purified using column chromatography (Hex/EtOAc 6:1 with 0.5 % AcOH) to provide 142 mg (88 %) of **(10)** as a white solid. **^1^H NMR** (500 MHz, CDCl_3_) δ 4.62 – 4.56 (m, 1H), 3.92 – 3.84 (m, 1H), 3.55 – 3.47 (m, 1H), 1.90 – 1.78 (m, 1H), 1.78 – 1.66 (m, 1H), 1.66 – 1.46 (m, 4H) ppm. **^13^C NMR** (126 MHz, CDCl_3_) δ 179.41, 98.78, 62.31, 30.77, 25.49, 19.66 ppm. **IR** 2944, 2194, 2089, 1700 cm^-1^. **Melting point** 43 – 44.5 °C. **HRMS** C_21_H_10_D_30_O_4_ [M+Na]^+^ calculated 409.4702; found 409.4703.

### 16-[(tetrahydro-2*H*-pyran-2-yl)oxy]hexadecan-2,2,3,3,4,4,5,5,6,6,7,7,8,8,9,9,10,10,11,11,12,12,13,13,14,14,15,15,16,16-*d*_30_-1-ol **(11)**

48 mg (0.12 mmol) of **(10)** was dried on high vacuum, dissolved in 3 mL of dry THF and cooled down to 0 °C in an ice bath under an Ar atmosphere. Subsequently, 260 µL (0.26 mmol) of 1M BH_3_·THF complex was added dropwise, temperature was slowly increased to room temperature and the reaction mixture was stirred over night. The reaction was quenched by an addition of MeOH and solvents were evaporated. This process was repeated two more times to remove all traces of BH_3_. The crude product was purified using column chromatography (Hex/EtOAc 2:1 with 0.5 % AcOH) to provide 42 mg (90 %) of **(11)** as a white solid. The reaction was repeated again with 70 mg of **(10)** with the same yield. Overall yield was then 112 mg of **(11)**. **^1^H NMR** (600 MHz, CDCl_3_) δ 4.58 – 4.53 (m, 1H), 3.90 – 3.82 (m, 1H), 3.61 (d, J = 6.1 Hz, 2H), 3.52 – 3.45 (m, 1H), 1.87 – 1.76 (m, 1H), 1.74 – 1.66 (m, 1H), 1.61 – 1.46 (m, 4H) ppm. **^13^C NMR** (151 MHz, CDCl_3_) δ 98.90, 63.04, 62.43, 30.89, 25.60, 19.80 ppm. **IR** 3424, 2899, 2195, 2088, 1441 cm^-1^. **Melting point** 43.5 – 45 °C. **HRMS** C_21_H_12_D_30_O_3_ [M+Na]^+^ calculated 395.4909; found 359.4909.

### 16-[(tetrahydro-2*H*-pyran-2-yl)oxy]hexadecanal-2,2,3,3,4,4,5,5,6,6,7,7,8,8,9,9,10,10,11,11,12,12,13,13,14,14,15,15,16,16-*d*_30_ **(12)**

33 µL (0.38 mmol) of oxalyl chloride was dissolved in 2 mL of dry DCM in an oven-dried flask under an Ar atmosphere and cooled down to -55 °C. 60 µL (0.84 mmol) of dry DMSO in 1 mL of dry DCM was added and the reaction mixture was stirred for 10 min. 101 mg (0.27 mmol) of **(11)** in 1 mL of dry DCM was added and the stirring continued for 90 min. Then 117 µL of dry triethylamine was added, the reaction mixture was slowly allowed to heat up to room temperature and it was stirred over night. Reaction was quenched by an addition of 10 mL of brine and it was extracted by 3 × 15 mL of CHCl_3_. Mixed organic phase was evaporated and the crude product was purified using column chromatography (Hex/EtOAc 15:1) to provide 66 mg (66 %) of **(12)** as a clear oil. **^1^H NMR** (600 MHz, CDCl_3_) δ 9.75 (t, J = 1.9 Hz, 1H), 4.58 – 4.54 (m, 1H), 3.89 – 3.83 (m, 1H), 3.52 – 3.45 (m, 1H), 1.86 – 1.78 (m, 1H), 1.74 – 1.66 (m, 1H), 1.63 – 1.43 (m, 4H) ppm. **^13^C NMR** (151 MHz, CDCl_3_) δ 203.12, 98.91, 62.45, 30.90, 25.60, 19.82 ppm. **IR** 2940, 2196, 2094, 1727 cm^-1^. **HRMS** C_21_H_10_D_30_O_3_ [M+Na]^+^ calculated 393.4753; found 393.4719.

### (*E/Z*)-32-[(tetrahydro-2*H*-pyran-2-yl)oxy]dotriacont-16-enoic-18,18,19,19,20,20,21,21,22,22,23,23,24,24,25,25,26,26,27,27,28,28,29,29,30,30,31,31,32,32-*d*_30_ acid **(13)**

195 mg (0.33 mmol) of (15-carboxypentadecyl)triphenylphosphonium bromide (prepared according to (1)) was dried on high vacuum, suspended in 5 mL of dry THF and cooled down to -20 °C under an Ar atmosphere. 750 µL (0.75 mmol) of 1M NaHMDS in THF was added dropwise and the reaction mixture was stirred for 3 hours. Then, 66 mg (0.18 mmol) of **(12)** in 3 mL of dry THF was added and cooling continued for additional 30 min. The reaction was stirred over night at room temperature. Reaction was quenched by an addition of 20 mL of saturated NH_4_Cl solution, acidified by concentrated HCl to pH 1 and extracted by 3 × 20 mL CHCl_3_. Crude product was purified using a column chromatography (Hex/EtOAc 15:1 with 0.5 % AcOH) to provide 65 mg (60 %) of **(13)** as a white solid. **^1^H NMR** (500 MHz, CDCl_3_) δ 5.41 – 5.31 (m, 2H), 4.62 – 4.57 (m, 1H), 3.93 – 3.85 (m, 1H), 3.56 – 3.48 (m, 1H), 2.35 (t, J = 7.5 Hz, 2H), 2.06 – 1.93 (m, 4H), 1.90 – 1.79 (m, 1H), 1.78 – 1.69 (m, 1H), 1.69 – 1.46 (m, 4H), 1.46 – 1.08 (m, 22H) ppm. **^13^C NMR** (126 MHz, CDCl_3_) δ 179.16, 129.91, 129.86, 98.77, 62.31, 33.92, 30.76, 29.76, 29.66, 29.64, 29.59, 29.55, 29.44, 29.30, 29.24, 29.06, 27.19, 26.97, 25.48, 24.69, 19.66 ppm. **IR** 2917, 2849, 2193, 2089, 1694, 1490 cm^-1^. **Melting point** 43.5 – 45 °C. **HRMS** C_37_H_40_D_30_O_4_ [M+Na]^+^ calculated 631.7049; found 631.7008.

### 2,5-dioxopyrrolidin-1-yl (*E/Z*)-32-[(tetrahydro-2*H*-pyran-2-yl)oxy]dotriacont-16-enoate-18,18,19,19,20,20,21,21,22,22,23,23,24,24,25,25,26,26,27,27,28,28,29,29,30,30,31,31,32,32-*d*_30_ **(14)**

63 mg (0.10 mmol) of **(13)** was mixed with 67 mg (0.20 mmol) of disuccinimidyl carbonate, dried on high vacuum and dissolved in 15 mL of dry DCM under an Ar atmosphere. Reaction mixture was cooled down to 0 °C in an ice bath and 50 µL (0.20 mmol) of dry diisopropylethylamine was added. Reaction mixture was stirred over night at room temperature. Solvent was evaporated and the crude product was purified using column chromatography (Hex/EtOAc 6:1 with 0.5 % AcOH) to provide 57 mg (78 %) of **(14)** as a white solid. **^1^H NMR** (500 MHz, CDCl_3_) δ 5.41 – 5.30 (m, 2H), 4.60 – 4.55 (m, 1H), 3.92 – 3.84 (m, 1H), 3.55 – 3.47 (m, 1H), 2.84 (s, 4H), 2.61 (t, J = 7.5 Hz, 2H), 2.07 – 1.98 (m, 4H), 1.90 – 1.66 (m, 4H), 1.63 – 1.47 (m, 4H), 1.45 – 1.18 (m, 20H) ppm. **^13^C NMR** (126 MHz, CDCl_3_) δ 169.15, 168.67, 129.90, 129.86, 98.79, 62.33, 30.93, 30.79, 29.77, 29.69, 29.67, 29.65, 29.62, 29.57, 29.55, 29.35, 29.32, 29.08, 28.79, 27.20, 27.00, 26.97, 25.57, 25.50, 24.56, 19.71 ppm. **IR** 2922, 2850, 2195, 2089, 1820, 1788, 1742, 1726, 1464, 1371 cm^-1^. **Melting point** 56 – 57 °C. **HRMS** C_41_H_43_D_30_NO_6_ [M+Na]^+^ calculated 728.7213; found 728.7173.

### 2,5-dioxopyrrolidin-1-yl 32-[(tetrahydro-2*H*-pyran-2-yl)oxy]dotriacontanoate-16,17,18,18,19,19,20,20,21,21,22,22,23,23,24,24,25,25,26,26,27,27,28,28,29,29,30,30,31,31,32,32-*d*_32_ **(15)**

55 mg (0.078 mmol) of **(14)** was dissolved in 15 mL of EtOAc in a double-necked flask, 6.5 mg (0.007 mmol) of 10 % PdC was added and the atmosphere was replaced with a D_2_ from a balloon. The reaction mixture was stirred over night at room temperature under a D_2_ atmosphere. Palladium was removed by filtration and the solvent was evaporated. ^1^H NMR indicated a complete reduction of the double bond, thus, the product was used for next reaction without further purification. Yield of the product was 52 mg (94 %). **^1^H NMR** (500 MHz, CDCl_3_:MeOD 5:1) δ 4.61 – 4.55 (m, 1H), 3.92 – 3.84 (m, 1H), 3.55 – 3.47 (m, 1H), 2.84 (s, 4H), 2.61 (t, J = 7.5 Hz, 2H), 1.89 – 1.79 (m, 1H), 1.79 – 1.66 (m, 3H), 1.64 – 1.47 (m, 4H), 1.46 – 1.36 (m, 2H), 1.36 – 1.07 (m, 24H) ppm. **^13^C NMR** (126 MHz, CDCl­_3_­­­­­­) δ 169.15, 168.67, 98.79, 62.33, 30.93, 30.79, 29.70, 29.68, 29.66, 29.62, 29.58, 29.55, 29.45, 29.34, 29.08, 28.78, 25.57, 25.50, 24.56, 19.70 ppm. **IR** 2920, 2850,2194, 2088, 1820, 1788, 1725, 1466, 1372 cm^-1^. **Melting point** 95 – 97 °C. **HRMS** C_41_H_43_D_32_NO_6_ [M+Na]^+^ calculated 732.7495; found 732.7483.

### 2,5-dioxopyrrolidin-1-yl 32-hydroxydotriacontanoate-16,17,18,18,19,19,20,20,21,21,22,22,23,23,24,24,25,25,26,26,27,27,28,28,29,29,30,30,31,31,32,32-*d*_32_ **(16)**

50 mg (0.07 mmol) of **(15)** was dissolved in 4 mL of MeOH and 40 mg (0.21 mmol) of *p*-toluenesulfonic acid monohydrate was added. The reaction mixture was stirred over night at room temperature. Solvent was evaporated and the crude product was purified using column chromatography (CHCl_3_/MeOH 100:1) to provide 39 mg (89 %) of **(16)** as a white solid. **^1^H NMR** (500 MHz, CDCl_3_) δ 2.76 (s, 4H), 2.51 (t, J = 7.5 Hz, 2H), 1.69 – 1.60 (m, 2H), 1.36 – 1.26 (m, 2H), 1.26 – 0.97 (m, 24H) ppm. **^13^C NMR** (126 MHz, CDCl_3_) δ 173.70, 172.85, 34.75, 33.55, 33.52, 33.48, 33.41, 33.21, 32.95, 32.95, 32.64, 29.44, 28.41 ppm. **IR** 3587, 2920, 2850, 2194, 2089, 1818, 1786, 1717, 1470, 1374 cm^-1^. **Melting point** 110 – 112 °C. **HRMS** C_36_H_35_D_32_NO_5_ [M+Na]^+^ calculated 648.6920; found 648.6902.

### 2,5-dioxopyrrolidin-1-yl 32-[((9*Z*,12*Z*)-octadeca-9,12-dienoyl)oxy]dotriacontanoate-16,17,18,18,19,19,20,20,21,21,22,22,23,23,24,24,25,25,26,26,27,27,28,28,29,29,30,30,31,31,32,32-*d*_32_ **(17)**

17 mg (0.027 mmol) of **(16)** was dried at high vacuum. 8 mg (0.03 mmol) of linoleic acid in 3.5 mL of dry THF was added under an Ar atmosphere and the reaction mixture was dilluted with additional 3.5 mL of dry THF. 5 µL (0.03 mmol) of 2,4,6-trichlorobenzoyl chloride and 10 µL (0.07 mmol) of dry triethylamine were subsequently added and the reaction mixture was stirred for 2 min at room temperature. 2 mg (0.016 mmol) of 4-(dimethylamino)pyridine was added and the reaction mixture was stirred over night at room temperature. Solvent was evaporated and the crude product was purified using column chromatography (CHCl_3_/MeOH 100:1) to provide 16 mg (67 %) of **(17)** as a white solid. **^1^H NMR** (500 MHz, CDCl_3_) δ 5.44 – 5.29 (m, 4H), 2.84 (s, 4H), 2.78 (t, J = 6.7 Hz, 2H), 2.63 – 2.58 (m, 2H), 2.34 – 2.26 (m, 2H), 2.06 (q, J = 7.0 Hz, 4H), 1.80 – 1.70 (m, 2H), 1.68 – 1.54 (m, 4H), 1.49 – 1.11 (m, 38H), 0.93 – 0.87 (m, 3H) ppm. **^13^C NMR** (126 MHz, CDCl_3_) δ 174.00, 169.16, 168.68, 130.20, 130.04, 128.02, 127.89, 34.39, 31.51, 30.93, 29.69, 29.66, 29.62, 29.58, 29.55, 29.34, 29.16, 29.11, 29.08, 28.78, 27.18, 25.61, 25.58, 25.00, 24.56, 22.56, 14.06 ppm. **IR** 2919, 2850, 2193, 2088, 1824, 1787, 1742, 1725, 1469, 1379 cm^-1^. **Melting point** 80 – 83 °C. **HRMS** C_54_H_65_D_32_NO_6_ [M+H]^+^ calculated 888.9397; found 888.9384.

### 32-[((2*S*,3*R*,*E*)-1,3-dihydroxyoctadec-4-en-2-yl)amino]-32-oxodotriacontyl-1,1,2,2,3,3,4,4,5,5,6,6,7,7,8,8,9,9,10,10,11,11,12,12,13,13,14,14,15,15,16,17-*d*_32_ (9*Z*,12*Z*)-octadeca-9,12-dienoate **(18)**

15 mg (0.017 mmol) of **(17)** was mixed with 7 mg (0.024 mmol) of sphingosine and together dried on high vacuum. 3 mL of dry THF and 1.5 mL of dry DCM were added under an Ar atmosphere followed by an addition of 9 µL (0.051 mmol) of dry diisopropylethylamine. The reaction mixture was stirred over night at room temperature. Solvents were evaporated and the crude product was purified using column chromatography (CHCl_3_/MeOH 50:1) to provide 12 mg (67 %) of **(18)** as a white solid. **^1^H NMR** (500 MHz, CDCl_3_) δ 6.25 (d, J = 7.5 Hz, 1H), 5.92 – 5.67 (m, 1H), 5.65 – 5.48 (m, 1H), 5.48 – 5.24 (m, 4H), 4.47 – 4.25 (m, 1H), 4.00 – 3.88 (m, 2H), 3.76 – 3.63 (m, 1H), 2.81 – 2.72 (m, 4H), 2.29 (t, J = 7.5 Hz, 2H), 2.27 – 2.16 (m, 2H), 2.11 – 2.02 (m, 6H), 1.70 – 1.58 (m, 4H), 1.44 – 1.15 (m, 60H), 0.95 – 0.82 (m, 6H) ppm. **^13^C NMR** (126 MHz, CDCl_3_) δ 174.03, 173.88, 134.28, 130.20, 130.04, 128.81, 128.03, 128.00, 127.90, 74.70, 62.52, 54.47, 36.84, 34.40, 32.27, 31.91, 31.52, 29.71, 29.69, 29.67, 29.65, 29.62, 29.59, 29.56, 29.51, 29.48, 29.37, 29.35, 29.34, 29.28, 29.21, 29.16, 29.13, 29.11, 27.19, 25.75, 25.62, 25.01, 22.68, 22.56, 14.10, 14.05 ppm. **IR** 3305, 2919, 2851, 2194, 2089, 1733, 1647, 1558, 1467, 1378 cm^-1^. **Melting point** 79 – 81 °C. **HRMS** C_68_H_97_D_32_NO_5_ [M+H]^+^ calculated 1073.1952; found 1073.1943; [M+Na]^+^ calculated 1095.1772; found 1095.1756.

### 12-bromododecanal-1,2,2,3,3,4,4,5,5,6,6,7,7,8,8,9,9,10,10,11,11,12,12-*d*_23_ **(19)**

305 µL (3.55 mmol) of oxalyl chloride was dissolved in 20 mL of dry DCM in an oven-dried flask under an Ar atmosphere. The flask was cooled down to -60 °C and 433 µL (6.09 mmol) of dry DMSO was added in 10 mL of dry DCM. After 10 min of stirring, 586 mg (2.03 mmol) of **(3)** in 10 mL of dry DCM was added and the reaction mixture was stirred at -60 °C for 90 min. After 90 min, 850 µL (6.09 mmol) of dry triethylamine was added, reaction mixture was slowly allowed to reach room temperature and it was further left stirring at room temperature over night under an Ar atmosphere. The reaction was quenched by an addition of brine and it was extracted with 3 × 15 mL of CHCl_3_. The mixed organic phase was evaporated and the crude product was purified using column chromatography (Hex/EtOAc 20:1) to provide 425 mg (73 %) of **(19)** as a colorless oil. **^1^H NMR** (500 MHz, CDCl_3_) no signals. **^13^C NMR** (126 MHz, CDCl_3_) no signals. **IR** 2198, 2096, 1712 cm^-1^.

### (*E/Z*)-16-bromohexadec-4-enoic-2,2,3,3,4,5,6,6,7,7,8,8,9,9,10,10,11,11,12,12,13,13,14,14,15,15,16,16-*d*_28_ acid **(20)**

2211 mg (5.08 mmol) of **(8)** was dried at high vacuum, then suspended in 10 mL of dry THF under an Ar atmosphere and cooled down to -30 °C. 11.6 mL (11.6 mmol) of 1M NaHMDS in THF was added dropwise and the mixture was stirred for additional 60 min. 415 mg (1.45 mmol) of **(19)** in 10 mL of THF was added and the cooling was continued for 30 min. The reaction was stirred over night at room temperature under an Ar atmosphere. Reaction was quenched by an addition of 40 mL of saturated NH_4_Cl, it was acidified with concentrated HCl to pH 1 and extracted with 3 × 100 mL of CHCl_3_. Mixed organic phase was evaporated and the crude product was purified using column chromatography (Hex/EtOAc 15:1 with 0.5 % AcOH) to provide 151 mg (29 %) of **(20)** as a clear oil. **^1^H NMR** (500 MHz, CDCl_3_) no signals. **^13^C NMR** (126 MHz, CDCl_3_) δ 179.33, 127.29, 126.64, 32.08 ppm. **IR** 2925, 2197, 2096, 1708, 1410, 1298 cm^-1^.

### (*E/Z*)-(15-carboxypentadec-12-en-1-yl-1,1,2,2,3,3,4,4,5,5,6,6,7,7,8,8,9,9,10,10,11,11,12,13,14,14,15,15-*d*_28_)triphenylphosphonium bromide **(21)**

140 mg (0.39 mmol) of **(20)** was mixed with 113 mg (0.43 mmol) of PPh_3_. The reaction mixture was stirred without solvent at 140 °C for 30 hours. After cooling down, the solid was dissolved in a minimal volume of CHCl_3_ and poured into a pre-cooled Et_2_O. The precipitate was filtered off and the procedure was repeated two more times to provide 206 mg (85 %) of **(21)** as a white solid. **^1^H NMR** (600 MHz, CDCl_3_) δ 7.70 – 7.62 (m, 6H), 7.57 – 7.50 (m, 3H), 7.49 – 7.41 (m, 6H) ppm. **^13^C NMR** (151 MHz, CDCl_3_) δ 132.26, 132.19, 132.10, 128.66, 128.57 ppm. **IR** 2925, 2854, 2196, 2096, 1701 cm^-1^.

### (4*E/Z*,16*E/Z*)-32-[(tetrahydro-2*H*-pyran-2-yl)oxy]dotriaconta-4,16-dienoic-2,2,3,3,4,5,6,6,7,7,8,8,9,9,10,10,11,11,12,12,13,13,14,14,15,15,16-*d*_27_ acid **(22)**

128 mg (0.21 mmol) of **(21)** was dried on high vacuum, suspended in 5 mL of dry THF and cooled down to -20 °C under an Ar atmosphere. 460 µL (0.46 mmol) of 1M NaHMDS in THF was added dropwise and the reaction mixture was stirred for 3 hours. Then, 215 mg (0.63 mmol) of 16-[(tetrahydro-2*H*-pyran-2-yl)oxy]hexadecanal (prepared according to (1)) in 3 mL of dry THF was added and cooling continued for additional 30 min. The reaction was stirred over night at room temperature. Reaction was quenched by an addition of 20 mL of saturated NH_4_Cl solution, acidified by concentrated HCl to pH 1 and extracted by 3 × 20 mL CHCl_3_. Crude product was purified using a column chromatography (Hex/EtOAc 15:1 with 0.5 % AcOH) to provide 48 mg (39 %) of **(22)** as a white solid. **^1^H NMR** (600 MHz, CDCl_3_) δ 5.38 – 5.29 (m, 1H), 4.59 – 4.55 (m, 1H), 3.90 – 3.82 (m, 1H), 3.75 – 3.68 (m, 1H), 3.53 – 3.46 (m, 1H), 3.41 – 3.34 (m, 1H), 2.33 (t, J = 7.5 Hz, 1H), 1.99 (q, J = 6.7 Hz, 1H), 1.87 – 1.76 (m, 1H), 1.74 – 1.67 (m, 1H), 1.65 – 1.43 (m, 8H), 1.43 – 1.10 (m, 22H) ppm. **^13^C NMR** (151 MHz, CDCl_3_) δ 179.27, 130.01, 129.90, 98.91, 67.81, 62.40, 34.01, 30.86, 29.87, 29.83, 29.77, 29.75, 29.69, 29.63, 29.57, 29.49, 29.40, 29.31, 29.13, 27.29, 26.32, 25.59, 24.78, 19.75 ppm. **IR** 2919, 2851, 2196, 2095, 1704, 1466, 1351 cm^-1^. **Melting point** 29 – 30 °C. **HRMS** C_37_H_41_D_27_O_4_ [M+Na]^+^ calculated 626.6705; found 626.6678.

### 2,5-dioxopyrrolidin-1-yl (4E/Z,16E/Z)-32-[(tetrahydro-2*H*-pyran-2-yl)oxy]dotriaconta-4,16-dienoate-2,2,3,3,4,5,6,6,7,7,8,8,9,9,10,10,11,11,12,12,13,13,14,14,15,15,16-*d*_27_ **(23)**

47 mg (0.08 mmol) of **(22)** was mixed with 41 mg (0.16 mmol) of disuccinimidyl carbonate, dried on high vacuum and dissolved in 12 mL of dry DCM under an Ar atmosphere. Reaction mixture was cooled down to 0 °C in an ice bath and 27 µL (0.16 mmol) of dry diisopropylethylamine was added. Reaction mixture was stirred over night at room temperature. Solvent was evaporated and the crude product was purified using column chromatography (Hex/EtOAc 6:1 with 0.5 % AcOH) to provide 39 mg (71 %) of **(23)** as a white solid. **^1^H NMR** (500 MHz, CDCl_3_) δ 5.39 – 5.30 (m, 1H), 4.61 – 4.56 (m, 1H), 3.92 – 3.84 (m, 1H), 3.78 – 3.69 (m, 1H), 3.55 – 3.47 (m, 1H), 3.43 – 3.35 (m, 1H), 2.84 (s, 4H), 2.67 – 2.56 (m, 1H), 2.24 – 1.93 (m, 1H), 1.89 – 1.79 (m, 1H), 1.78 – 1.66 (m, 1H), 1.66 – 1.48 (m, 6H), 1.38 – 1.22 (m, 24H) ppm. **^13^C NMR** (126 MHz, CDCl_3_) δ 169.12, 169.06, 129.90, 129.81, 98.83, 67.69, 62.32, 30.78, 29.78, 29.75, 29.68, 29.65, 29.60, 29.56, 29.54, 29.49, 29.34, 29.32, 27.21, 26.24, 25.58, 25.51, 19.69 ppm. **IR** 2921, 2852, 2196, 2091, 1817, 1789, 1743, 1647, 1464, 1373 cm^-1^. **Melting point** 41 – 43 °C. **HRMS** C_41_H_44_D_27_NO_6_ [M+Na]^+^ calculated 723.6868; found 723.6848.

### 2,5-dioxopyrrolidin-1-yl 32-[(tetrahydro-2*H*-pyran-2-yl)oxy]dotriacontanoate-2,2,3,3,4,4,5,5,6,6,7,7,8,8,9,9,10,10,11,11,12,12,13,13,14,14,15,15,16,16,17-*d*_31_ **(24)**

13 mg (0.019 mmol) of **(23)** was dissolved in 5 mL of EtOAc in a double-necked flask, 1.6 mg (0.0017 mmol) of 10 % PdC was added and the atmosphere was replaced with a D_2_ from a balloon. The reaction mixture was stirred over night at room temperature under a D_2_ atmosphere. Palladium was removed by filtration and the solvent was evaporated. ^1^H NMR indicated a complete reduction of the double bond, thus, the product was used for next reaction without further purification. Yield of the product was 12 mg (91 %), white solid. **^1^H NMR** (600 MHz, CDCl_3_) δ 4.63 – 4.51 (m, 1H), 3.90 – 3.80 (m, 1H), 3.77 – 3.67 (m, 1H), 3.53 – 3.44 (m, 1H), 3.44 – 3.31 (m, 1H), 2.82 (s, 4H), 2.65 – 2.50 (m, 1H), 2.40 – 2.23 (m, 1H), 1.90 – 1.45 (m, 6H), 1.44 – 1.04 (m, 27H) ppm. **^13^C NMR** (151 MHz, CDCl_3_) δ 169.24, 98.93, 67.80, 62.43, 32.01, 30.89, 29.85, 29.78, 29.58, 29.44, 26.33, 25.68, 25.61, 22.77, 19.79, 14.19, 1.10 ppm. **IR** 2920, 2850, 2194, 2088, 1820, 1741, 1726, 1558, 1465, 1375 cm^-1^. **Melting point** 62 – 66 °C. **HRMS** C_41_H_44_D_31_NO_6_ [M+Na]^+^ calculated 731.7432; found 731.7422.

### 2,5-dioxopyrrolidin-1-yl 32-hydroxydotriacontanoate-2,2,3,3,4,4,5,5,6,6,7,7,8,8,9,9,10,10,11,11,12,12,13,13,14,14,15,15,16,16,17-*d*_31_ **(25)**

10 mg (0.014 mmol) of **(24)** was dissolved in 2 mL of MeOH and 8 mg (0.042 mmol) of *p*-toluenesulfonic acid monohydrate was added. The reaction mixture was stirred over night at room temperature. Solvent was evaporated and the crude product was purified using column chromatography (CHCl_3_/MeOH 100:1) to provide 5 mg (57 %) of **(25)** as a white solid. **^1^H NMR** (600 MHz, CDCl_3_) δ 3.65 – 3.59 (m, 2H), 2.83 (s, 4H), 1.62 – 1.51 (m, 2H), 1.41 – 1.14 (m, 27H) ppm. **^13^C NMR** (151 MHz, CDCl_3_) δ 169.24, 63.21, 32.91, 29.78, 29.69, 29.52, 25.82, 25.68 ppm. **IR** 2920, 2850, 2194, 2088, 1817, 1785, 1736, 1466, 1376 cm^-1^. **Melting point** 83 – 86 °C. **HRMS** C_36_H_36_D_31_NO_5_ [M+Na]^+^ calculated 647.6857; found 647.6846.

### 2,5-dioxopyrrolidin-1-yl 32-[((9*Z*,12*Z*)-octadeca-9,12-dienoyl)oxy]dotriacontanoate-2,2,3,3,4,4,5,5,6,6,7,7,8,8,9,9,10,10,11,11,12,12,13,13,14,14,15,15,16,16,17-*d*_31_ **(26)**

4.5 mg (0.0072 mmol) of **(25)** was dried at high vacuum. 2.2 mg (0.0079 mmol) of linoleic acid in 1 mL of dry THF was added under an Ar atmosphere and the reaction mixture was dilluted with additional 1 mL of dry THF. 1.2 µL (0.0079 mmol) of 2,4,6-trichlorobenzoyl chloride and 2.5 µL (0.018 mmol) of dry triethylamine were subsequently added and the reaction mixture was stirred for 2 min at room temperature. 0.5 mg (0.0043 mmol) of 4-(dimethylamino)pyridine was added and the reaction mixture was stirred over night at room temperature. Solvent was evaporated and the crude product was purified using column chromatography (CHCl_3_/MeOH 100:1) to provide 2.3 mg (36 %) of **(26)** as an oil that solidified upon standing. **^1^H NMR** (500 MHz, CDCl_3_) δ 5.41 – 5.32 (m, 4H), 4.06 (t, J = 6.7 Hz, 2H), 2.85 (s, 4H), 2.78 (t, J = 6.7 Hz, 2H), 2.61 (t, J = 7.6 Hz, 2H), 2.30 (t, J = 7.5 Hz, 2H), 2.06 (q, J = 7.0 Hz, 4H), 1.75 (q, J = 7.5 Hz, 2H), 1.70 – 1.48 (m, 6H), 1.48 – 1.13 (m, 35H), 1.00 – 0.77 (m, 3H) ppm. **^13^C NMR** (126 MHz, CDCl_3_) δ 178.76, 177.69, 130.22, 128.08, 127.90, 64.77, 40.22, 31.92, 29.70, 29.00, 27.20, 25.59, 22.69, 22.57, 14.12 ppm. **IR** 2921, 2851, 2193, 2088, 1741, 1578, 1464, 1377 cm^-1^. **HRMS** C_54_H_66_D_31_NO_6_ [M+H]^+^ calculated 887.9335; found 887.9315.

### 32-[((2*S*,3*R*,*E*)-1,3-dihydroxyoctadec-4-en-2-yl)amino]-32-oxodotriacontyl-16,17,17,18,18,19,19,20,20,21,21,22,22,23,23,24,24,25,25,26,26,27,27,28,28,29,29,30,30,31,31-*d*_31_ (9*Z*,12*Z*)-octadeca-9,12-dienoate **(27)**

2 mg (0.0023 mmol) of **(26)** was mixed with 1 mg (0.0034 mmol) of sphingosine and together dried on high vacuum. 1 mL of dry THF and 0.5 mL of dry DCM were added under an Ar atmosphere followed by an addition of 1.2 µL (0.0069 mmol) of dry diisopropylethylamine. The reaction mixture was stirred over night at room temperature. Solvents were evaporated and the crude product was purified using column chromatography (CHCl_3_/MeOH 50:1) to provide 2.1 mg (88 %) of **(27)** as a white solid. **^1^H NMR** (600 MHz, CDCl_3_) δ 6.20 (d, J = 7.6 Hz, 1H), 5.82 – 5.74 (m, 1H), 5.63 – 5.47 (m, 1H), 5.46 – 5.23 (m, 4H), 4.33 – 4.30 (m, 1H), 4.04 (t, J = 6.7 Hz, 2H), 3.98 – 3.87 (m, 2H), 3.72 – 3.68 (m, 1H), 2.76 (t, J = 6.9 Hz, 2H), 2.57 – 2.54 (m, 2H), 2.28 (t, J = 7.6 Hz, 2H), 2.08 – 2.00 (m, 6H), 1.80 – 1.44 (m, 10H), 1.44 – 1.14 (m, 55H), 0.92 – 0.78 (m, 6H) ppm. **^13^C NMR** (151 MHz, CDCl_3_) δ 128.92, 74.89, 64.51, 62.66, 32.35, 32.01, 29.78, 29.44, 29.21, 27.29, 26.03, 22.77, 14.20 ppm. **IR** 2918, 2851, 2192, 2088, 1737, 1611, 1547, 1467, 1377, 1262 cm^-1^. **Melting point** 100 – 102 °C. **HRMS** C_68_H_98_D_31_NO_5_ [M+H]^+^ calculated 1072.1889; found 1072.1886; [M+Na]^+^ calculated 1094.1709; found 1094.1702.

### 2,5-dioxopyrrolidin-1-yl 32-[((9*Z*,12*Z*)-octadeca-9,12-dienoyl-*d*_31_)oxy]dotriacontanoate **(28)**

172 mg (0.29 mmol) of 2,5-dioxopyrrolidin-1-yl 32-hydroxydotriacontanoate (prepared according to (1)) was dried at high vacuum. 100 mg (0.32 mmol) of linoleic acid-*d*_32_ in 35 mL of dry THF was added under an Ar atmosphere and the reaction mixture was dilluted with additional 35 mL of dry THF. 50 µL (0.32 mmol) of 2,4,6-trichlorobenzoyl chloride and 100 µL (0.72 mmol) of dry triethylamine were subsequently added and the reaction mixture was stirred for 2 min at room temperature. 21 mg (0.17 mmol) of 4-(dimethylamino)pyridine was added and the reaction mixture was stirred over night at room temperature. Solvent was evaporated and the crude product was purified using column chromatography (CHCl_3_/MeOH 100:1) to provide 191 mg (74 %) of **(28)** as a white solid. **^1^H NMR** (500 MHz, CDCl_3_) δ 4.06 (t, J = 6.8 Hz, 2H), 2.87 – 2.82 (m, 4H), 2.61 (t, J = 7.5 Hz, 2H), 1.80 – 1.70 (m, 2H), 1.67 – 1.57 (m, 2H), 1.54 – 1.36 (m, 2H), 1.37 – 1.15 (m, 52H) ppm. **^13^C NMR** (126 MHz, CDCl_3_) δ 174.03, 169.14, 168.67, 128.00, 64.37, 31.37, 30.31, 29.70, 29.62, 29.58, 29.55, 29.53, 29.42, 29.35, 29.25, 29.08, 28.79, 28.65, 25.93, 25.58, 24.57 ppm. **IR** 3447, 2919, 2849, 2198, 2095, 1825, 1788, 1741, 1726, 1473, 1275, 1209, 1071 cm^-1^. **Melting point** 80 – 83 °C. **HRMS** C_54_H_66_D_31_NO_6_ [M+H]^+^ calculated 887.9334; found 887.9317; [M+Na]^+^ calculated 909.9154; found 909.9155.

### 2,5-dioxopyrrolidin-1-yl 32-[((9*Z*,12*Z*)-octadeca-9,12-dienoyl-2,3,4,5,6,7,8,9,10,11,12,13,14,15,16,17,18-^13^C_18_)oxy]dotriacontanoate **(29)**

91 mg (0.15 mmol) of 2,5-dioxopyrrolidin-1-yl 32-hydroxydotriacontanoate (prepared according to (1)) was dried at high vacuum. 50 mg (0.17 mmol) of linoleic acid-^13^C_18_ in 19 mL of dry THF was added under an Ar atmosphere and the reaction mixture was dilluted with additional 19 mL of dry THF. 26 µL (0.17 mmol) of 2,4,6-trichlorobenzoyl chloride and 53 µL (0.38 mmol) of dry triethylamine were subsequently added and the reaction mixture was stirred for 2 min at room temperature. 11 mg (0.09 mmol) of 4-(dimethylamino)pyridine was added and the reaction mixture was stirred over night at room temperature. Solvent was evaporated and the crude product was purified using column chromatography (CHCl_3_/MeOH 100:1) to provide 71 mg (53 %) of **(29)** as a white solid. **^1^H NMR** (500 MHz, CDCl_3_) δ 5.68 – 5.36 (m, 2H), 5.37 – 5.12 (m, 2H), 4.50 – 4.32 (m, 2H), 4.06 (td, J = 6.7, 2.9 Hz, 2H), 3.03 – 2.77 (m, 6H), 2.76 – 2.53 (m, 4H), 2.25 – 2.08 (m, 4H), 2.04 – 1.83 (m, 2H), 1.82 – 1.69 (m, 6H), 1.68 – 1.57 (m, 4H), 1.53 – 1.10 (m, 60H), 1.08 – 0.96 (m, 2H), 0.85 – 0.68 (m, 1H) ppm. **^13^C NMR** (126 MHz, CDCl_3_) δ 174.18, 173.73, 169.14, 130.85 – 129.34 (m), 128.66 – 127.17 (m), 66.68, 64.40, 35.26 – 33.68 (m), 31.51 (td, J = 34.3, 3.3 Hz), 30.16 – 28.28 (m), 27.16 (ddt, J = 41.6, 34.1, 3.8 Hz), 26.40 – 24.29 (m), 22.55 (td, J = 34.5, 3.9 Hz), 14.05 (dd, J = 34.6, 3.8 Hz) ppm. **IR** 3446, 2918, 2850, 1822, 1788, 1742, 1726, 1695, 1471, 1370, 1273, 1209, 1071 cm^-1^. **Melting point** 84 – 87 °C. **HRMS** C_36_^13^C_18_H_97_NO_6_ [M+H]^+^ calculated 874.7993; found 874.7993; [M+Na]^+^ calculated 896.7812; found 896.7819.

### 32-[((2*S*,3*R*,*E*)-1,3-dihydroxyoctadec-4-en-2-yl)amino]-32-oxodotriacontyl (9*Z*,12*Z*)-octadeca-9,12-dienoate-*d*_31_ **(30)**

50 mg (0.056 mmol) of **(28)** was mixed with 20 mg (0.067 mmol) of sphingosine and together dried on high vacuum. 9 mL of dry THF and 4.5 mL of dry DCM were added under an Ar atmosphere followed by an addition of 30 µL (0.168 mmol) of dry diisopropylethylamine. The reaction mixture was stirred over night at room temperature. Solvents were evaporated and the crude product was purified using column chromatography (CHCl_3_/MeOH 50:1) to provide 52 mg (87 %) of **(30)** as a white solid. **^1^H NMR** (500 MHz, CDCl_3_) δ 6.25 (d, J = 7.6 Hz, 1H), 5.92 – 5.71 (m, 1H), 5.67 – 5.47 (m, 1H), 4.45 – 4.24 (m, 1H), 4.06 (t, J = 6.8 Hz, 2H), 4.01 – 3.80 (m, 2H), 3.77 – 3.57 (m, 1H), 2.37 – 2.16 (m, 2H), 2.16 – 1.98 (m, 2H), 1.70 – 1.57 (m, 4H), 1.54 – 1.01 (m, 76H), 0.89 (t, J = 6.9 Hz, 3H) ppm. **^13^C NMR** (126 MHz, CDCl_3_) δ 173.88, 134.27, 128.80, 74.70, 64.39, 62.52, 54.45, 36.84, 32.27, 31.91, 29.70, 29.67, 29.64, 29.61, 29.57, 29.52, 29.51, 29.48, 29.36, 29.28, 29.25, 29.21, 29.10, 28.64, 25.92, 25.75, 22.68, 14.11 ppm. **IR** 3342, 2917, 2850, 2199, 2095, 1732, 1615, 1549, 1472, 1277 cm^-1^. **Melting point** 82 – 83.5 °C. **HRMS** C_68_H_98_D_31_NO_5_ [M+H]^+^ calculated 1072.1889; found 1072.1879; [M+Na]^+^ calculated 1094.1709; found 1094.1700.

### 32-oxo-32-[((2*S*,3*S*,4*R*)-1,3,4-trihydroxyoctadecan-2-yl)amino]dotriacontyl (9*Z*,12*Z*)-octadeca-9,12-dienoate-*d*_31_ **(31)**

30 mg (0.034 mmol) of **(28)** was mixed with 13 mg (0.041 mmol) of phytosphingosine and together dried on high vacuum. 6 mL of dry THF and 3 mL of dry DCM were added under an Ar atmosphere followed by an addition of 18 µL (0.123 mmol) of dry diisopropylethylamine. The reaction mixture was stirred over night at room temperature. Solvents were evaporated and the crude product was purified using column chromatography (CHCl_3_/MeOH 50:1) to provide 34 mg (93 %) of **(31)** as a white solid. **^1^H NMR** (500 MHz, CDCl_3_) δ 6.35 (d, J = 7.2 Hz, 1H), 4.27 – 4.11 (m, 1H), 4.06 (t, J = 6.8 Hz, 2H), 3.98 – 3.88 (m, 2H), 3.84 – 3.68 (m, 1H), 3.68 – 3.48 (m, 1H), 2.24 (t, J = 7.6 Hz, 2H), 1.71 – 1.54 (m, 6H), 1.54 – 1.41 (m, 2H), 1.39 – 1.04 (m, 76H), 1.00 – 0.70 (m, 3H) ppm. **^13^C NMR** (126 MHz, CDCl_3_) δ 170.68, 170.61, 71.68, 68.42, 60.55, 57.04, 47.97, 45.23, 45.06, 44.89, 44.72, 44.55, 32.54, 29.04, 27.85, 26.47, 25.64, 25.30, 24.53, 21.74, 18.61, 9.96 ppm. **IR** 3311, 2918, 2850, 2470, 2200, 2095, 1736, 1637, 1544, 1471, 1278 cm^-1^. **Melting point** 106 – 108 °C. **HRMS** C_68_H_100_D_31_NO_6_ [M+H]^+^ calculated 1090.1995; found 1090.1992; [M+Na]^+^ calculated 1112.1814; found 1112.1818.

### 32-[((2*S*,3*R*,*E*)-1,3-dihydroxyoctadec-4-en-2-yl)amino]-32-oxodotriacontyl (9*Z*,12*Z*)-octadeca-9,12-dienoate-1,2,3,4,5,6,7,8,9,10,11,12,13,14,15,16,17,18-^13^C_18_ **(32)**

37 mg (0.042 mmol) of **(29)** was mixed with 15 mg (0.05 mmol) of sphingosine and together dried on high vacuum. 7 mL of dry THF and 3.5 mL of dry DCM were added under an Ar atmosphere followed by an addition of 22 µL (0.126 mmol) of dry diisopropylethylamine. The reaction mixture was stirred over night at room temperature. Solvents were evaporated and the crude product was purified using column chromatography (CHCl_3_/MeOH 50:1) to provide 35 mg (78 %) of **(32)** as a white solid. **^1^H NMR** (500 MHz, CDCl_3_) δ 6.26 (d, J = 7.6 Hz, 1H), 5.94 – 5.67 (m, 1H), 5.66 – 5.43 (m, 1H), 4.39 (t, J = 6.6 Hz, 1H), 4.36 – 4.30 (m, 1H), 4.06 (td, J = 6.7, 2.9 Hz, 1H), 4.01 – 3.87 (m, 2H), 3.78 – 3.67 (m, 1H), 3.00 – 2.85 (m, 1H), 2.86 – 2.74 (m, 2H), 2.71 – 2.59 (m, 1H), 2.49 – 2.37 (m, 1H), 2.31 – 2.11 (m, 4H), 2.11 – 2.01 (m, 2H), 2.01 – 1.86 (m, 1H), 1.85 – 1.71 (m, 1H), 1.71 – 1.56 (m, 5H), 1.56 – 1.07 (m, 102H), 1.07 – 0.97 (m, 1H), 0.89 (t, J = 6.9 Hz, 2H), 0.84 – 0.70 (m, 1H) ppm. **^13^C NMR** (126 MHz, CDCl_3_) δ 174.24, 173.78, 134.26, 131.11 – 129.40 (m), 128.80, 128.53 – 126.99 (m), 74.66, 62.50, 54.47, 36.83, 35.23 – 33.60 (m), 32.27, 31.51 (td, J = 34.5, 3.3 Hz), 30.43 – 28.20 (m), 27.16 (ddt, J = 41.5, 34.0, 3.9 Hz), 26.23 – 24.36 (m), 22.54 (td, J = 34.5, 4.0 Hz), 14.05 (dd, J = 34.6, 4.0 Hz) ppm. **IR** 2917, 2850, 1741, 1693, 1614, 1549, 1472, 1274 cm^-1^. **Melting point** 85 – 87 °C. **HRMS** C_50_^13^C_18_H_129_NO_5_ [M+H]^+^ calculated 1059.0547; found 1059.0542; [M+Na]^+^ calculated 1081.0367; found 1081.0363.

32-[((2*S*,3*R*,*E*)-1,3-dihydroxyoctadec-4-en-2-yl)amino]-32-oxodotriacontyl-1,1,2,2,3,3,4,4,5,5,6,6,7,7,8,8,9,9,10,10,11,11,12,12,13,13,14,14,15,15,16,17-*d*_32_ (9*Z*,12*Z*)-octadeca-9,12-dienoate **(18)**


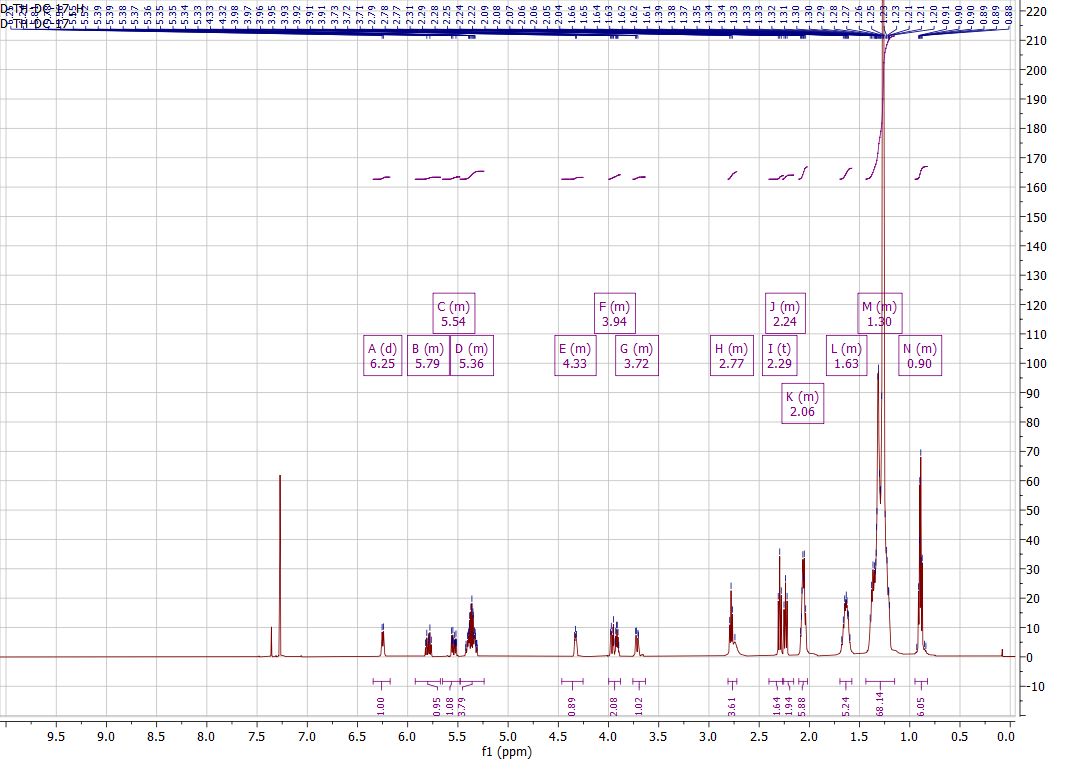


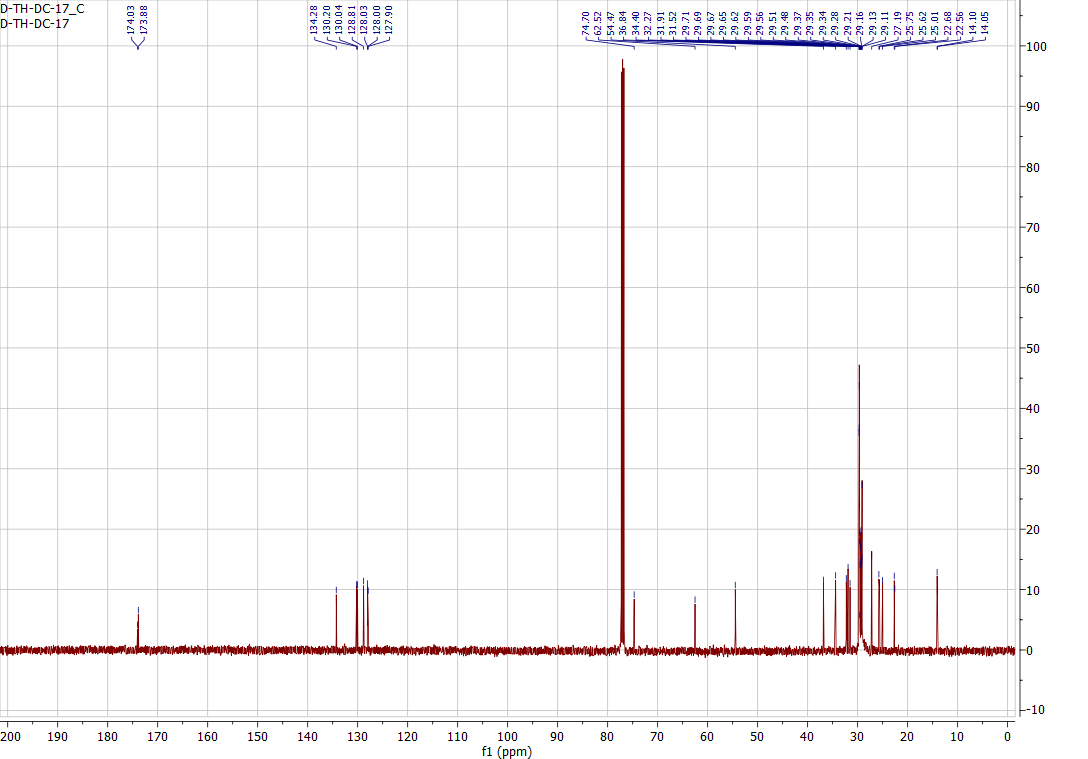


32-[((2*S*,3*R*,*E*)-1,3-dihydroxyoctadec-4-en-2-yl)amino]-32-oxodotriacontyl-16,17,17,18,18,19,19,20,20,21,21,22,22,23,23,24,24,25,25,26,26,27,27,28,28,29,29,30,30,31,31-*d*_31_ (9*Z*,12*Z*)-octadeca-9,12-dienoate **(27)**


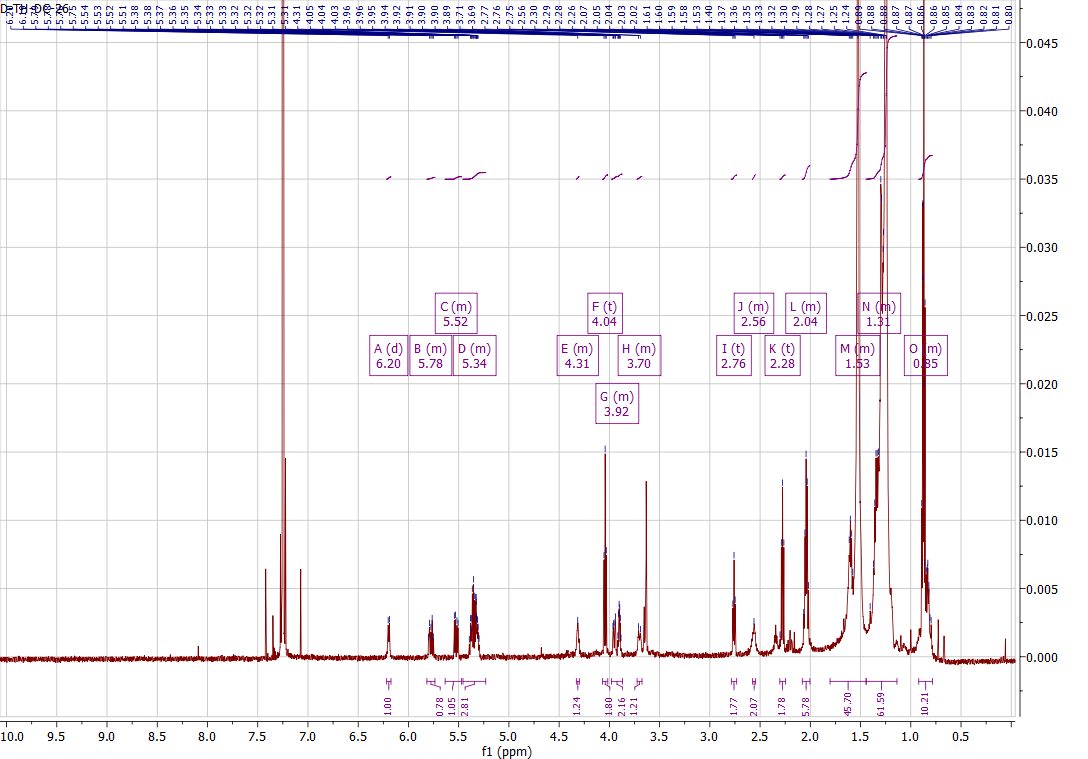


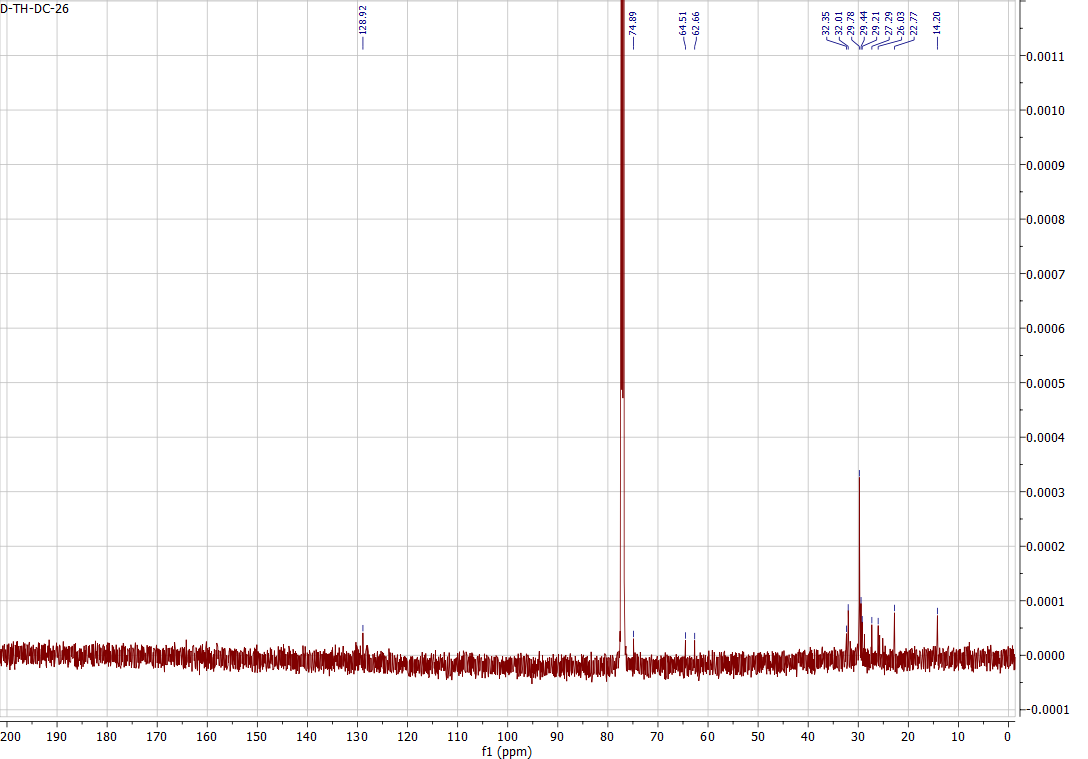


32-[((2*S*,3*R*,*E*)-1,3-dihydroxyoctadec-4-en-2-yl)amino]-32-oxodotriacontyl (9*Z*,12*Z*)-octadeca-9,12-dienoate-*d*_31_ **(30)**


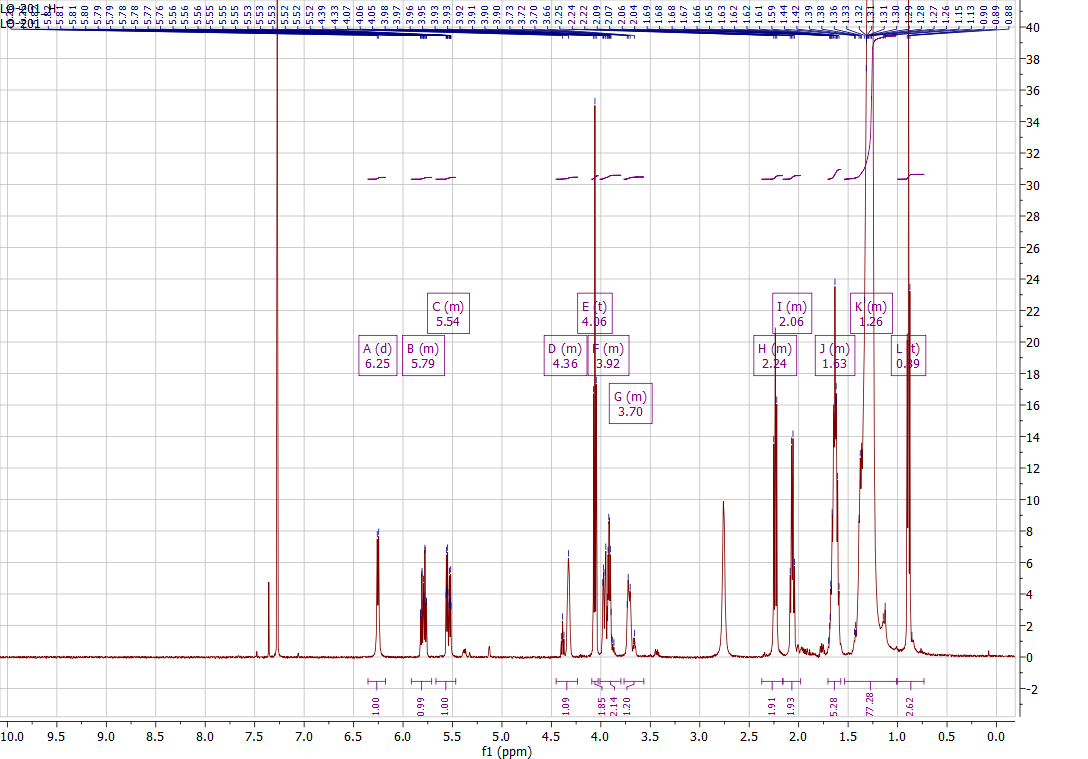


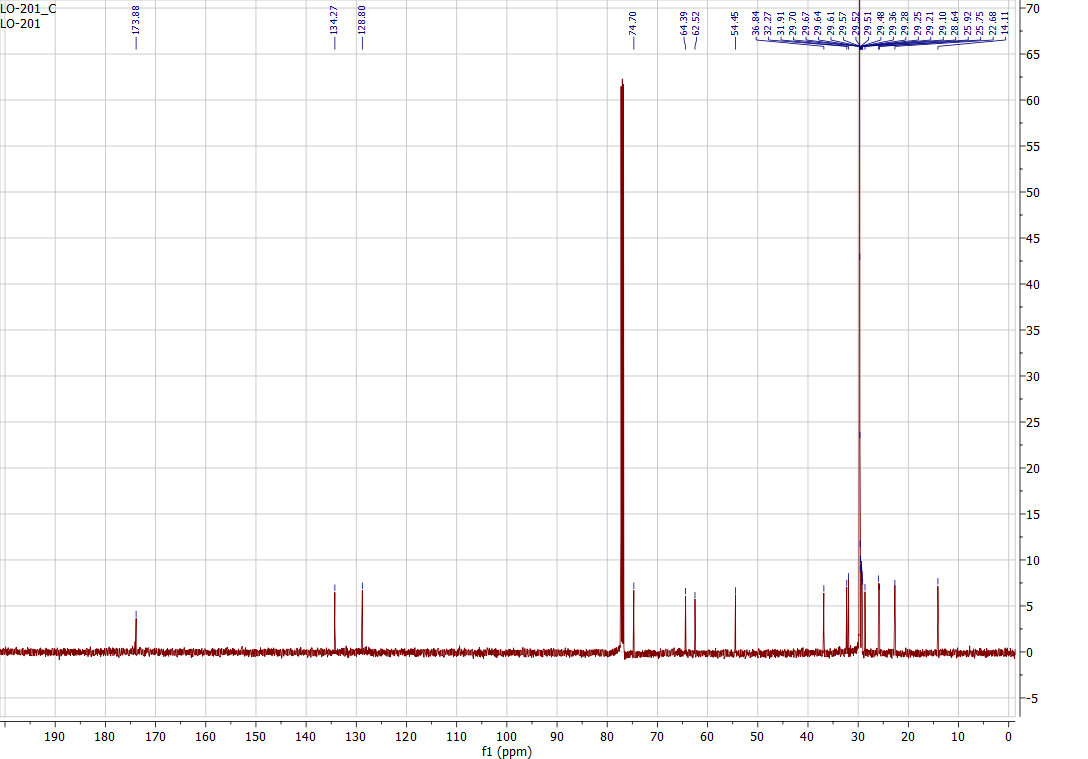


32-oxo-32-[((2*S*,3*S*,4*R*)-1,3,4-trihydroxyoctadecan-2-yl)amino]dotriacontyl (9*Z*,12*Z*)-octadeca-9,12-dienoate-*d*_31_ **(31)**


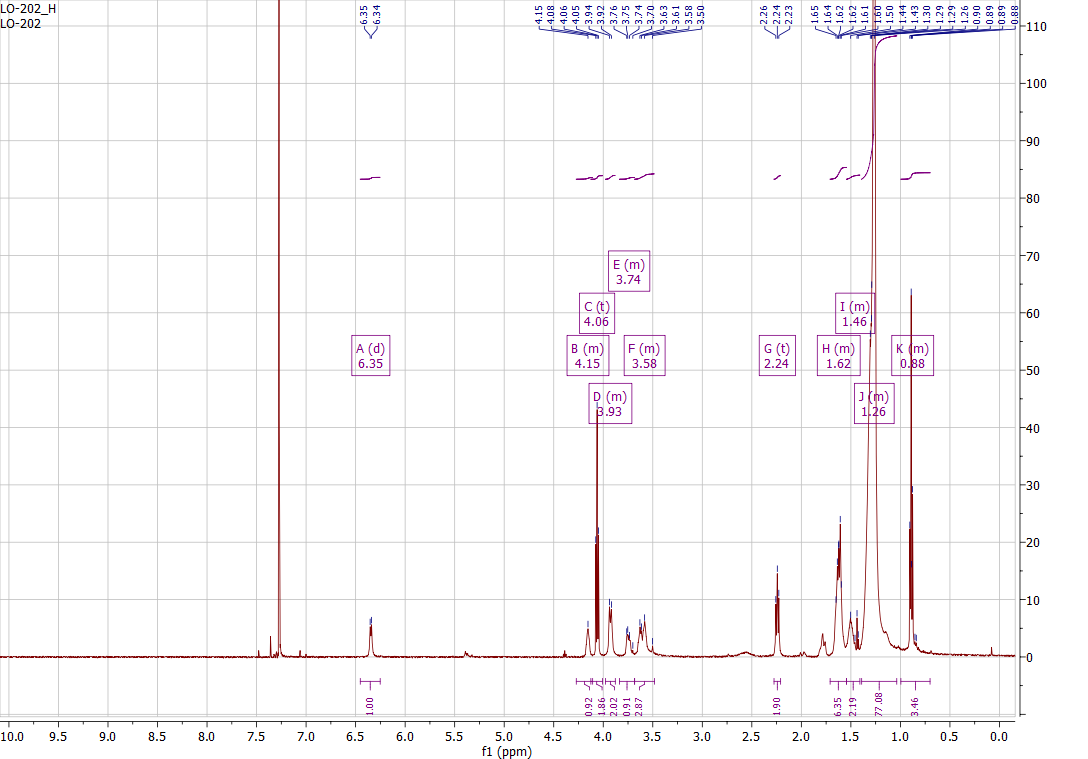


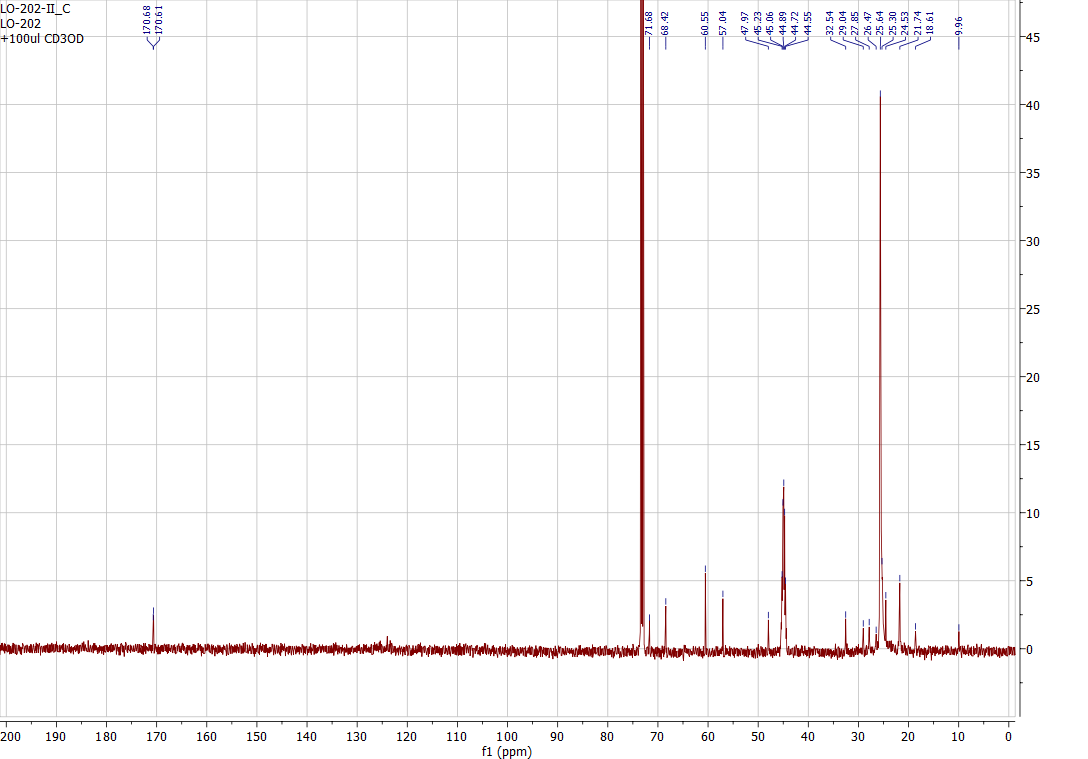


32-[((2*S*,3*R*,*E*)-1,3-dihydroxyoctadec-4-en-2-yl)amino]-32-oxodotriacontyl (9*Z*,12*Z*)-octadeca-9,12-dienoate-1,2,3,4,5,6,7,8,9,10,11,12,13,14,15,16,17,18-^13^C_18_ **(32)**


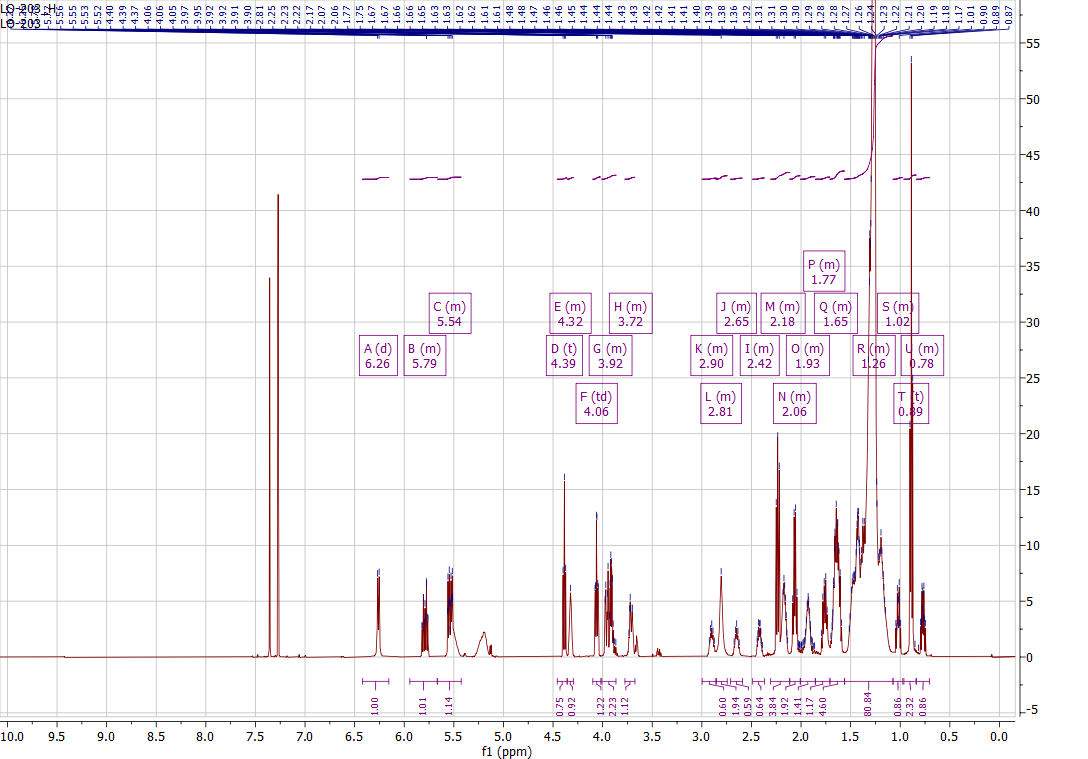


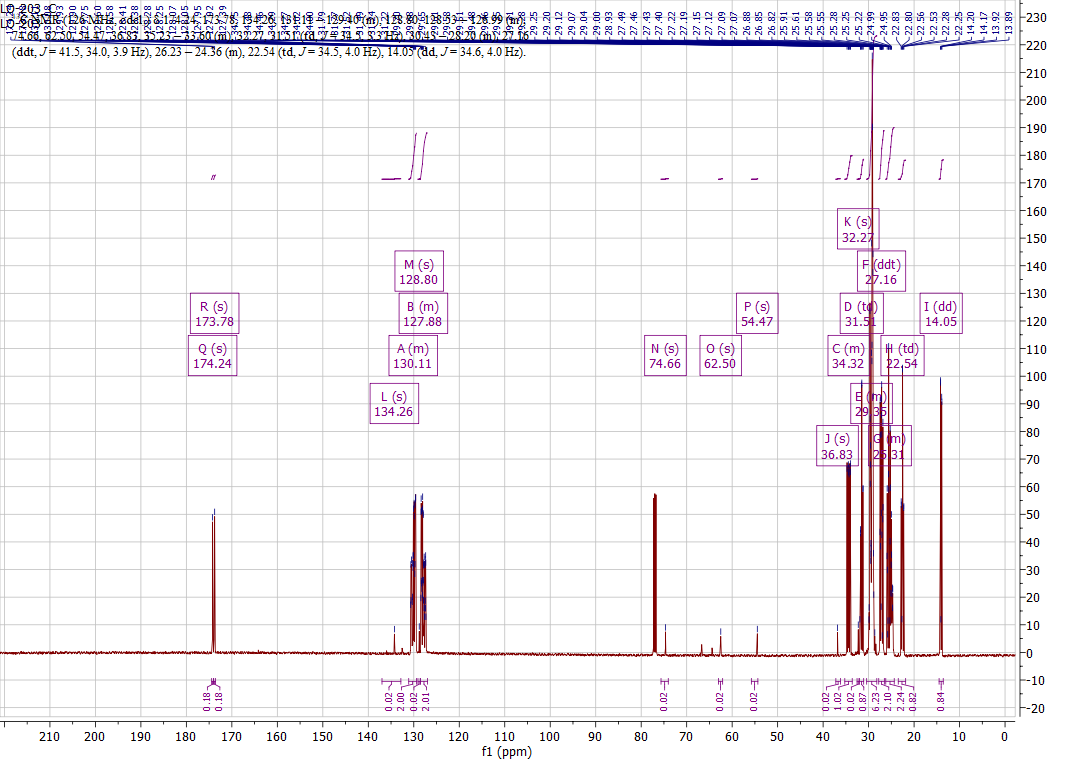


Error analysis in the determination of the phase proportion from the ^2^H NMR spectra

In order to estimate the error of the weights found in Figs. 3 and 4, we used a Monte Carlo (MC) approach to determine the impact of spectrum noise on the uncertainty of the weights. For each experimental spectrum, we take the original spectrum and add noise with the same root mean square variation (RMS) as the RMS of the original spectrum. The resulting new spectrum is then fitted with the same set of parameters as the original fit. For example, for Cer[NS] at 50°C, fit parameters were two order parameters, corresponding to methylene carbons and the methyl group of the fluid phase; a third order parameter is fixed to zero for the isotropic phase. For each order parameter, we also fit a linewidth and a weight of that component, yielding a total of 8 (2+3+3) free parameters. This process was repeated 600 times for different randomly generated noise. Standard deviations of the weights were then evaluated over the 600 copies. This procedure is representative of the error brought about by variation of the spectral noise, but will not include contributions from any systematic errors in modeling.

A few important notes about this process should be made. First, synthetic noise is produced using white noise that is subsequently processed with the same processing parameters as the original spectrum, to ensure noise characteristics close to the experimental noise (‘noise_gen.m’ in INFOS (2)). Second, we add the noise to the original spectrum, so that the fitted spectra actually have a total RMS of $\sqrt{2}*RMS_{\exp}$, although the part of the noise that actually varies during the MC analysis has the correct RMS. Then, we might expect an overestimate of the error. However, we tested our approach for Cer[NS] at 32°C, where the experimental spectrum was replaced by a noiseless synthetic spectrum (to which we then add the noise). The resulting errors were very similar to those obtained using the experimental spectrum. For this reason, we decided the safest approach is to risk a slight overestimate of the standard deviation of the weighting parameters, rather than overstate confidence in our results. Spectral fits for each step in the MC analysis were performed using a nested-fitting approach, that is, non-linear parameters (order parameters, line broadenings) were fitted using a trust-region-reflective algorithm (9) as implemented in the MATLAB Optimization Toolbox “lsqnonlin.m” function. Within each step of this outer fit, the optimal set of weights were obtained using a linear fitting algorithm as the inner fit (‘lsqlin.m’ in MATLAB) (10). Since the linear algorithms are faster/more stable, this yields an overall better performance since it reduces the number of free parameters in the non-linear algorithm. At each step in the Monte Carlo analysis, the starting points of the parameters were randomly set, although within 10% of the original fit, to avoid having the parameters fall into local minima which are not as well-fit as the original, and also to avoid having parameter “swaps” (where, for example, a large and small order parameter get switched in the fit– not actually changing the fit significantly, but appearing as large error in the MC). Finally, it is important to note the when we calculate the standard deviation of the weights, we first sum together all components contributing to the given phase, and take the standard deviation of the result. This eliminates the influence of covariance of the weights of different components within the same phase (for example, methyl and methylene contributions to the fluid phase in Cer[NS]).

The results for the manual adjustment as well as the Monte Carlo fit are reported in Table S1 below. Note that in a few cases, the manual fits found in main text Figure 3/4 do not necessarily agree with the automated fits used in the Monte Carlo analysis, although the manual fits give very good agreement with the experimental spectrum. In this case, the manual fit typically satisfies some physical requirement, e.g. the methyl peak’s *S* should be approximately 1/3 of the methylene peaks, although for LA-*d*_47_ at 25 ℃, the automated fit finds a much smaller value. In this case, we find the manual fits more reliable, but note that there is a high degree of uncertainty in the weights of the different phases, which may not be fully captured by the Monte Carlo analysis. Cases of disagreement between automated and manual fits’ disagreement can be found in the table.

**Table S1.** Phase contributions in the Cer[EOS]/Cer[NS]/FFA/Chol mixture determined from the ^2^H NMR spectra fitting the individual spectral components.

|  | Cer[NS]-*d*_47_, manual adjustment | | | |
| --- | --- | --- | --- | --- |
| Temperature | crystalline / % | *S* = 0.7 component / % | fluid / % | isotropic /% |
| 25°C | 99 | - | - | 1 |
| 32°C | 45 | 36 | 19 | - |
| 50°C | - | - | 97 | 3 |
| 65°C | - | - | - | 100 |
|  | Cer[NS]-*d*_47_, Monte Carlo fit | | | |
| 25°C | 95 ± 3 | - | - | 5 ± 3 |
| 32°C | 45 ± 6 | 40 ± 2 | 15 ± 6 | <1 |
| 50°C | - | - | 98 ± 1 | 2 ± 1 |
| 65°C | - | - | - | 100 |

|  | LA-*d*_47_, manual adjustment | | | |
| --- | --- | --- | --- | --- |
| Temperature | crystalline / % | *S* = 0.7 component / % | fluid / % | isotropic /% |
| 25°C | 89 |  |  | 11 |
| 32°C | 60 | 23 | 14 | 3 |
| 50°C | - | - | 89 | 11 |
| 65°C | - | - | - | 100 |
|  | LA-*d*_47_, Monte Carlo fit | | | |
| 25°C | 89 ± 10 | - | - | 11 ± 10 |
| 32°C | 49 ± 4 | 24 ± 5 | 24 ± 5 | 4 ± 1 |
| 50°C | - | - | 92 ± 1 | 8 ± 1 |
| 65°C | - | - | - | 100 |

|  | Chol-*d*_6_, manual adjustment | | | |
| --- | --- | --- | --- | --- |
| Temperature | crystalline / % | *S* = 0.7 component / % | fluid / % | isotropic /% |
| 25°C | 91 | - | 6 | 3 |
| 32°C | 64 | - | 33 | 3 |
| 50°C | - | - | 91 | 9 |
| 65°C | - | - | - | 100 |
|  | Chol-*d*_6_, Monte Carlo fit | | | |
| 25°C | 83 ± 5 | - | 12 ± 4 | 5 ± 3 |
| 32°C | 55 ± 6 | - | 44 ± 6 | 1 ± 4 |
| 50°C | - | - | 87 ± 6 | 13 ± 6 |
| 65°C | - | - | - | 100 |

|  | Cer[EOS]-sphingo-*d*_9_, manual adjustment | | |
| --- | --- | --- | --- |
| Temperature | crystalline / % | fluid / % | isotropic /% |
| 25°C | - | 95 | 5 |
| 32°C | - | 95 | 5 |
| 50°C | - | 95 | 5 |
| 65°C | - | - | 100 |
|  | Cer[EOS]-sphingo-*d*_9_, Monte Carlo fit | | |
|  |  |  |  |
| 25°C | - | 96 ± 2 | 4 ± 1 |
| 32°C | - | 91 ± 2 | 9 ± 2 |
| 50°C | - | 97 ± 2 | 3 ± 2 |
| 65°C | - | - | 100 |

|  | Cer[EOS]-upper-*d*_31_, manual adjustment | | |
| --- | --- | --- | --- |
| Temperature | crystalline / % | fluid / % | isotropic /% |
| 25°C | 71 | 12 | 17 |
| 32°C | 53 | 22 | 25 |
| 50°C | - | 44 | 56 |
| 65°C | - | - | 100 |
|  | Cer[EOS]-upper-*d*_31_, Monte Carlo fit | | |
|  |  |  |  |
| 25°C | 42 ± 1 | 42 ± 1 | 16 ± 1 |
| 32°C | 53 ± 3 | 33 ± 4 | 14 ± 1 |
| 50°C | - | 58 ± 2 | 42 ± 2 |
| 65°C | - | - | 100 |

|  | Cer[EOS]-middle-*d*_32_, manual adjustment | | |
| --- | --- | --- | --- |
| Temperature | crystalline / % | fluid / % | isotropic /% |
| 25°C | 47 | 7 | 46 |
| 32°C | 27 | 19 | 54 |
| 50°C | - | 49 | 51 |
| 65°C | - | - | 100 |
|  | Cer[EOS]-middle-*d*_32_, Monte Carlo fit | | |
|  |  |  |  |
| 25°C | 42 ± 2 | 8 ± 2 | 50 ± 1 |
| 32°C | 16 ± 1 | 29 ± 1 | 55 ± 1 |
| 50°C | - | 53 ± 1 | 47 ± 1 |
| 65°C | - | - | 100 |

^13^C NMR Relaxation Analysis

^13^C relaxation consisted of 3 experiment types at different fields: ^13^C *T*_1_ at 400 and 600 MHz ^1^H frequency, ^1^H–^13^C steady-state heteronuclear NOE at 400 and 600 MHz, and ^13^C *T*_1_*_ρ_* at 600 MHz ^1^H frequency with spin lock (*B*_1_) strengths on ^13^C of 11.4 and 22.9 kHz. Pulse sequences are shown in Supplemental Figure S1.


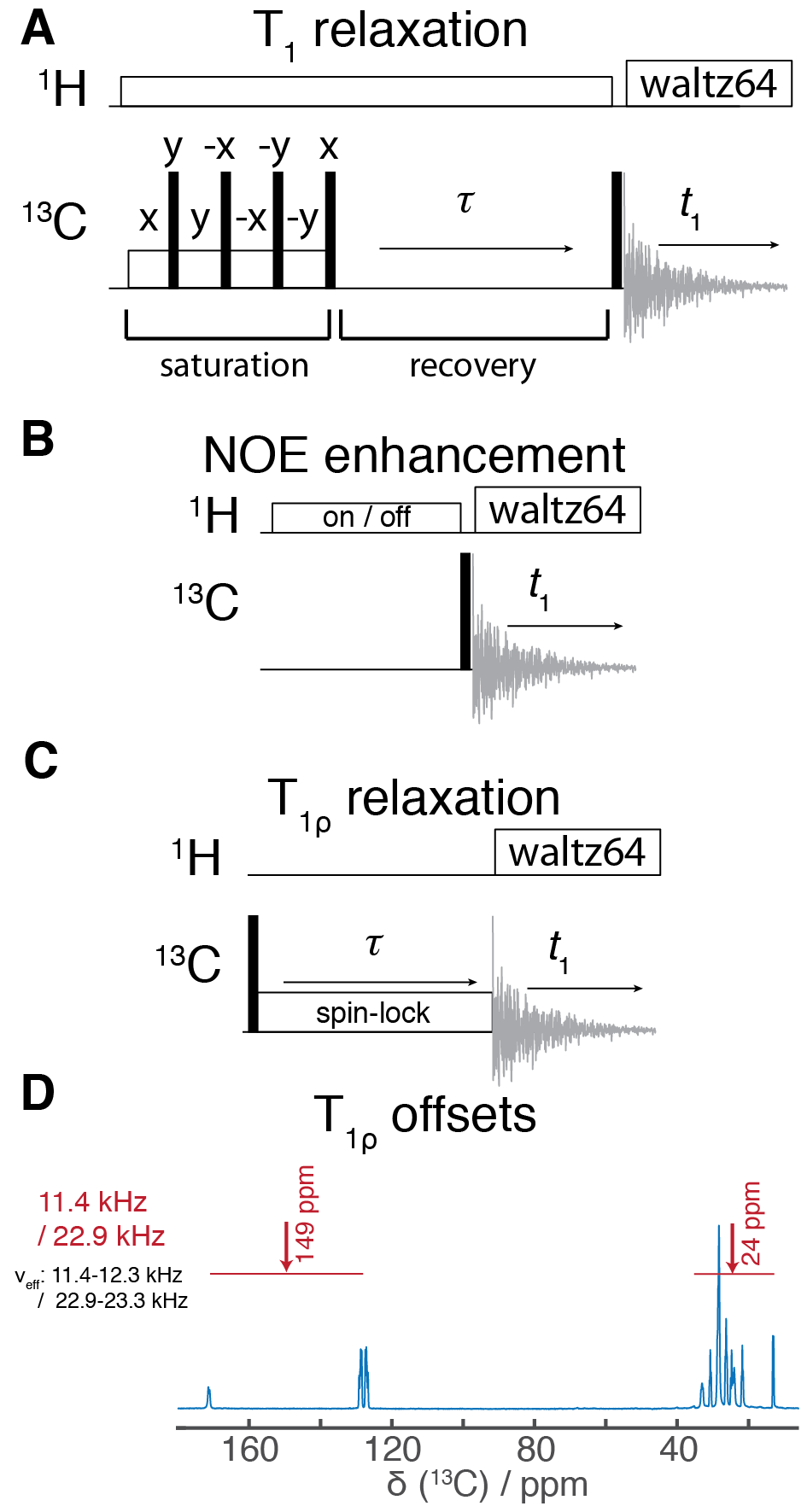


Supplemental Figure S1. Pulse sequences used for ^13^C relaxation. A shows the sequence for ^13^C saturation recovery including low-power saturation of the ^1^H during relaxation. B shows the sequence for NOE steady state enhancement. Enhancement is obtained by recording the sequence twice, once with ^1^H saturation and once without (on/off). Saturation delay is 10 s. C shows the pulse sequence for ^13^C T_1_*_ρ_* relaxation. D illustrates the placement of carrier frequencies for the T_1_*_ρ_* experiment where the effective spin lock strength varies between 11.4 and 12.3 kHz for one T_1_*_ρ_* experiment and between 22.9 and 23.3 kHz for the other experiment.

Experimental details are shown in Table . NOE steady-state experiments were performed with a 10 s ^1^H saturation period, where the enhancement is determined by acquiring the experiment once with low-power ^1^H saturation and once without saturation. ^13^C T_1_*_ρ_* are acquired for each spin-lock strength using two different ^13^C carrier frequencies, such that the effective field strength ($\sqrt{\omega_{1}^{2}+\Delta{\omega_{0}}^{2}}$) does not vary significantly for different resonances (i.e. $\sqrt{\omega_{1}^{2}+\Delta{\omega_{0}}^{2}}\approx\omega_{1}$). Carrier positions are shown in Supplemental Figure S1D, along with the range of effective fields resulting from those carrier positions.

Table S2: Experimental parameters for ^13^C relaxation measurements

| Experiment | *ω*_0H_/2π  [MHz] | *ω*_r_/2π [kHz] | *ω*_1_/2π [kHz] | Scans | Repetitions | Time Points | Start | Stop |
| --- | --- | --- | --- | --- | --- | --- | --- | --- |
| ^13^C T_1­_ | 600 | 6 | – | 128 | 2 | 21+1 | 10 ms | 10 s |
| ^1^H–^13^C NOE | 600 | 6 | – | 512 | 2+2 (on/off) | – | – | – |
| ^13^C T_1_ | 400 | 6 | – | 256 | 3 | 22+1 | 5 ms | 8 s |
| ^1^H–^13^C NOE | 400 | 6 | – | 256 | 3+3 (on/off) | – | – | – |
| ^13^C T_1_*_ρ_* | 600 | 6 | 22.9 | 96 | 2 (x2) | 18 | 200 μs | 41.8 ms |
| ^13^C T_1_*_ρ_* | 600 | 6 | 11.4 | 96 | 2 (x2) | 18 | 200 μs | 41.8 ms |

**Relaxation measurement data analysis.** ^13^C NMR relaxation data of the linoleoyl moiety of Cer[EOS] was analyzed in MATLAB, using the INFOS software. Before analysis, each 1D spectrum was baseline corrected. Then, a reference spectrum was produced by summing up all spectra in a series. The reference spectrum is fitted using an initial chemical shift list, assuming exponential signal decay (par.d1.Broad=’Lorentz’ in INFOS). The ^13^C spectrum and INFOS initial fit is shown in Supplemental Figure S2. Peak positions, linewidths, and amplitudes are variable in this initial fit. Subsequently, the full series of spectra were fitted simultaneously, using the FitTrace function in INFOS. In this fit, positions and linewidths were held fixed to their values in the initial fit, whereas the amplitudes and relaxation constants were allowed to vary.


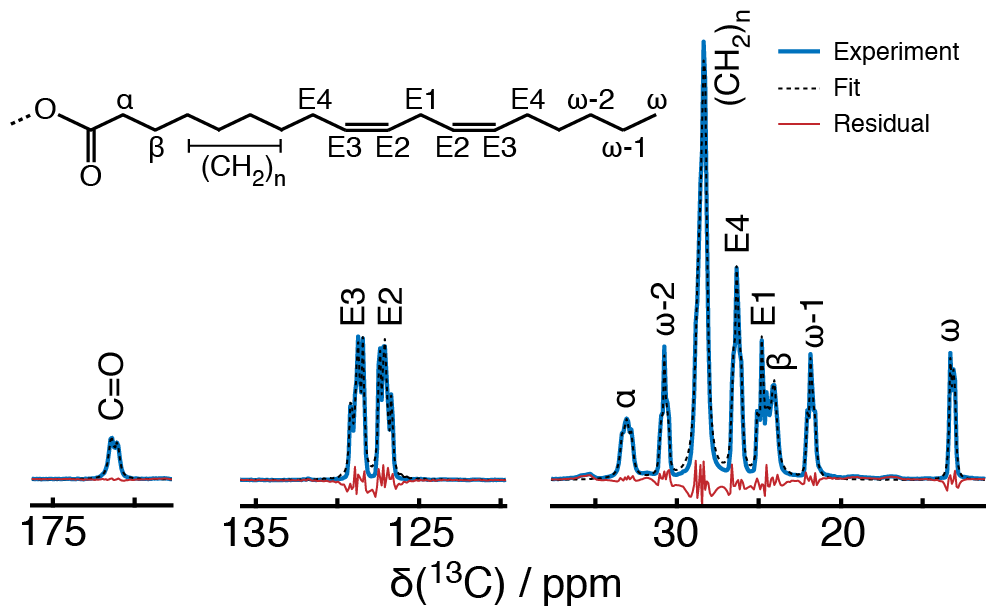


Supplemental Figure S2. Experimental ^13^C Cer[EOS] linoleic acid chain solid-state NMR spectrum, assignment, and INFOS fit. The linoleic acid chain is shown in the upper left with positions labeled on the molecule that correspond to labeled positions in the NMR spectrum. The experimental spectrum is shown as a blue solid line, the INFOS fit as a black dashed line, and the fit residual as a red line.

NOE results are reported as the rate constant for transfer from ^1^H to ^13^C ($\Gamma_{HC}$). This was obtained by fitting on and off spectra from the NOE, and calculating

| $\eta=\frac{I_{\text{on}}}{I_{\text{off}}}-1$  $\Gamma_{\text{HC}}=\eta*R_{\text{1,C}}\frac{\gamma_{\text{C}}}{\gamma_{\text{H}}}\frac{1}{n_{\text{H}}}$ | (1) |
| --- | --- |

Here, $R_{\text{1,C}}$ is the measured ^13^C NMR *R*_1_ rate, which is scaled by the number of bonded protons ($n_{\text{H}}$).

Error analysis was performed by fitting the individual spectra (as opposed to the full series) and extracting the peak intensities. This fit was done using the initial fit as starting point, and only allowing peak intensities to vary. Peak intensities were then fitted separately for each resonance, to the appropriate exponential decay or buildup function.

| $I_{i,\text{exp}}=A\exp\left( -t_{i}*R \right)+\varepsilon_{i}$  –or–  $I_{i,\text{exp}}=A(1-\exp\left( -t_{i}*R \right))+\varepsilon_{i}$ | (2) |
| --- | --- |

The errors are stored ($\varepsilon_{i}$), and a bootstrap data set is constructed as follows:

| $I_{i,\text{exp}}=A\exp\left( -t_{i}*R \right)+\varepsilon_{j}$ | (3) |
| --- | --- |

where $\varepsilon_{j}$ was randomly selected with replacement from the original data set. The new curve was fitted, and parameters stored. This is repeated 200 times, where the standard deviation of *R* is extracted from the bootstrapped curves is reported.

For error of the NOE experiments, standard deviation of $I_{\text{on}}$ and $I_{\text{off}}$ was determined using the ‘FitError’ function in INFOS (using default settings). Error of $\Gamma_{\text{HC}}$ is then determined using standard propagation-of-certainty rules, where we also account for the standard deviation obtained for the *R*_1_ measurement.

The resulting relaxation rate constants are shown below (bars) in addition to the back-calculated relaxation rate constants resulting from the detector analysis shown in main text Figure 6.


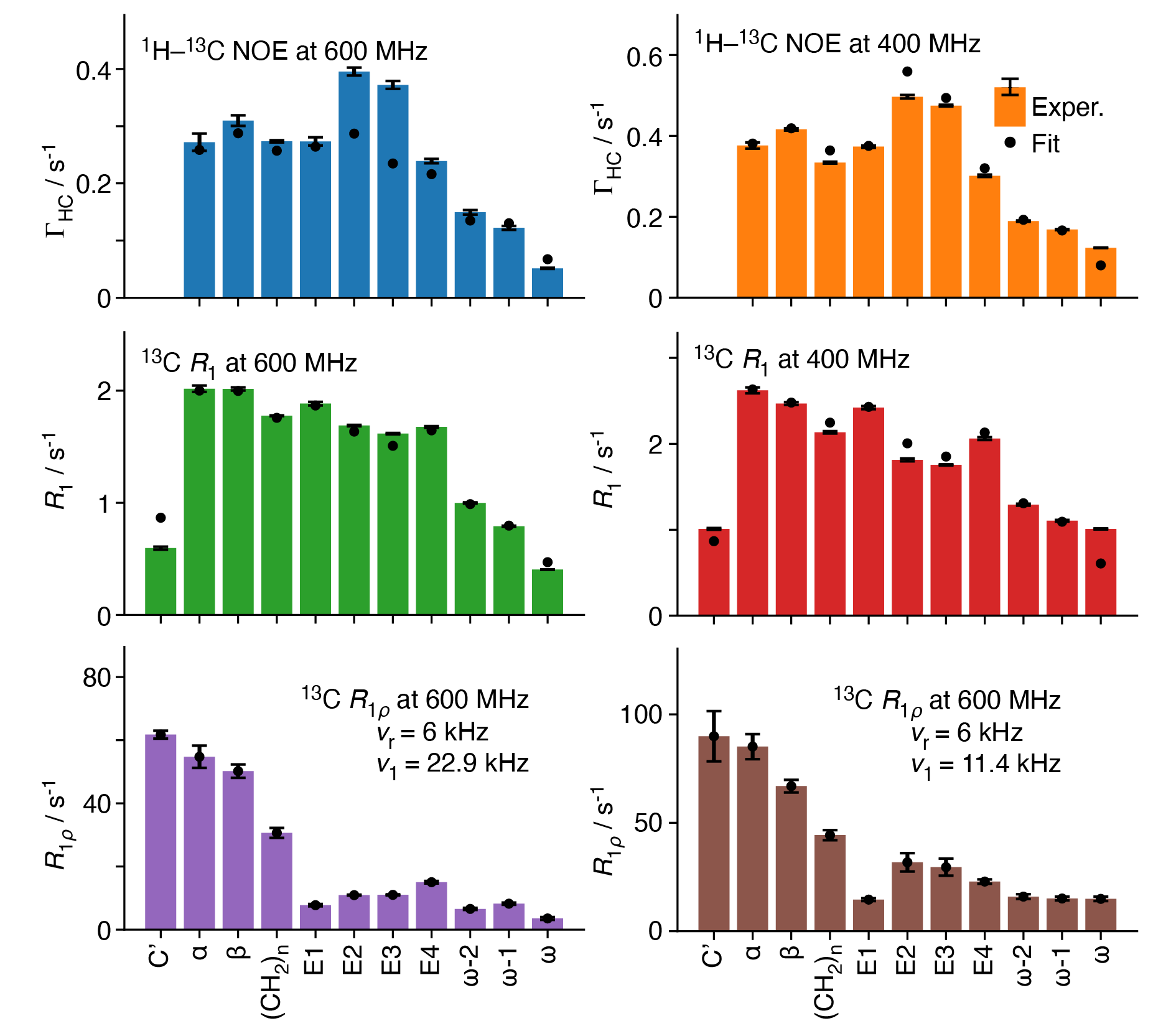


Supplemental Figure S3. Experiment relaxation rate constants and fitted rate constants resulting from detector analysis (analysis in main text Figure 6). Bars show experimental relaxation rate constants for the 6 experiments, error bars show $\boldsymbol{\pm}\boldsymbol{\sigma}$, and scatter points show the fitted relaxation I rate constants.

Neutron diffraction

The 1D diffraction patterns were analyzed as follows.
The 2θ angle was converted to scattering vector *q* [nm^-1^] according to equation:

$q=\frac{4\pi\sin(\theta)}{\lambda}$, [1]

where λ is the wavelength of the radiation and θ is the half of the scattering angle 2θ.

The ND reflections were fitted by the Gaussian-Lorentzian Cross Product function using OriginPro 2019b software. The background was fitted either by the linear or by the two-phase exponential decay function by the same software. The fitted peak parameters served for further calculations. The intensities *I_h_* (fitted peak areas) were corrected for the incident flux (*C_flux_*), the sample absorption (*C_abs_*), and the Lorentz factor (*C_Lor_*) according to (3) using the following equations 2, 3, 5.

$C_{flux}= \frac{1}{erf\left( \frac{L\sin\left( \theta\right)}{\sqrt{8}\sigma} \right)}$, [2]

where *erf* is the error function, *L* is the sample length (cm) and 2σ is the beam width (cm).

$C_{abs}= \frac{\frac{2\mu t}{\sin\left( \theta\right)}}{1-{exp}^{\frac{-2\mu t}{\sin\left( \theta\right)}}}$, [3]

where *t* is the sample thickness (cm), and μ (cm^-1^) is the absorption coefficient. The sample thickness *t* was estimated from the applied amount of lipid, sample area, and assumptive dry lipid density (ρ ≈ 0.87 g.cm^-3^) reported in (4). The absorption coefficient μ was calculated from the total neutron cross-section (i.e., the sum of the coherent and incoherent scattering cross-sections and absorption cross-section) of all the elements at the used wavelength (data from the NIST Center for Neutron Research), from the density and chemical composition of the sample according to the equation:

$\mu= \frac{\sum N_{i}\varphi_{i}}{V}$, [4]

where *N_i_* is the number of specific atoms *i*, *φ_i_* is the total neutron cross-section, and *V* is the volume of the sample. The hydration of the sample at various contrast conditions was included in the calculation of μ so we added 2 water molecules per lipid (4). The Lorentz correction was calculated as:

$C_{Lor}= \sin\left( 2\theta\right)$. [5]

The Lorentz correction in equation 5 applies for the well-aligned (oriented) lamellae and can be different for samples with more mosaic spread (5). The corrected values of scattering form factors for various orders *h* were calculated using the equation:

$\left| F_{h} \right|= \sqrt{C_{flux}C_{abs}C_{Lor}I_{h}}$, [6]

where *I_h_* is the scattering intensity of the individual order *h*. The phase angles of the centrosymmetric structure attain values of either 1 or -1 (6). The relative neutron scattering length density (NSLD, *ρ_(z)_*) profiles across the lipid layer with the origin (z = 0 nm) in its middle were calculated according to the equation:

$\rho_{\left( z \right)}= \sum_{h=1}^{h_{max}} F_{h}\cos\left( \frac{2\pi hz}{d} \right).$ [7]

The form factor errors δ*F_h_* were derived from the uncertainties of experimental intensities expressed as the square root of the integrated intensity *I_h_* of each reflection and recalculated to the uncertainties of the reconstructed NSLD profiles. The uncertainties of the relative NSLD profile (δρ_(z)_) were calculated according to (7) from the following equation:

${\delta\rho}_{(z)}=\pm\left[ \sum_{h=1}^{h_{max}} \left( {\delta F}_{h} \right)^{2}\cos^{2} \left( \frac{2\pi hz}{d} \right) \right]^{\frac{1}{2}}$. [8]


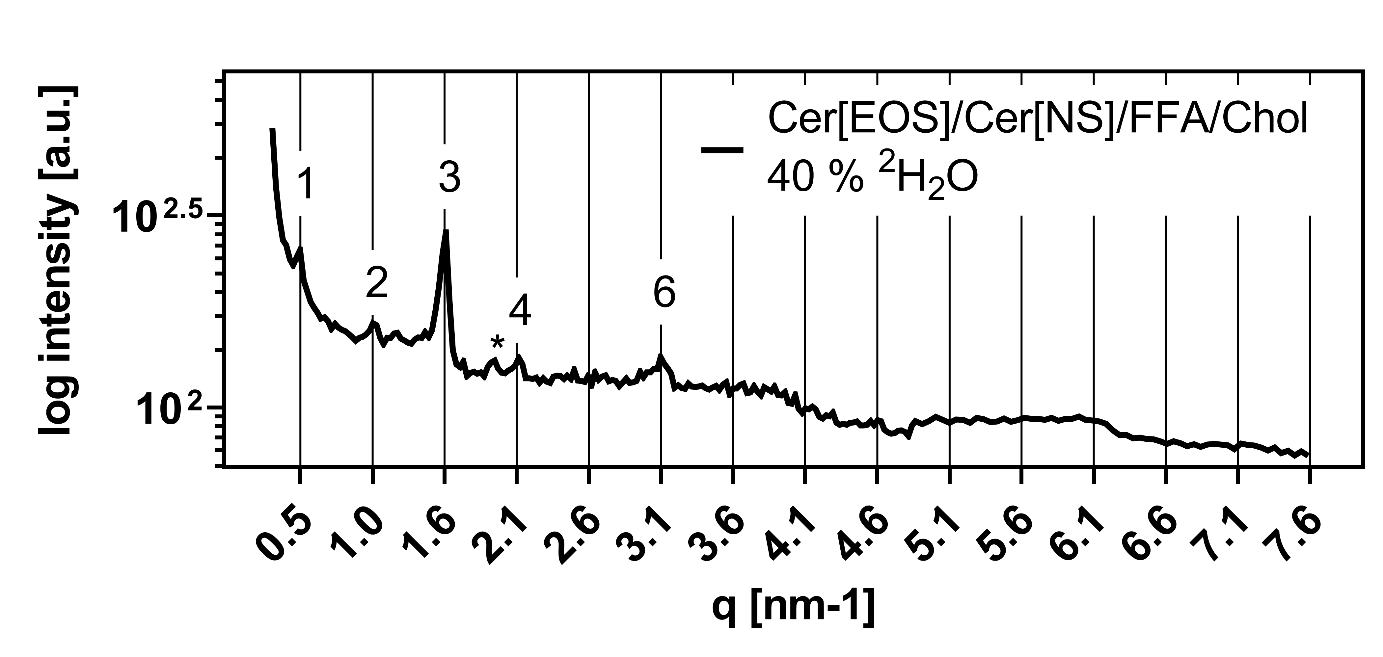


**Supplemental Figure S4.** The neutron diffraction (ND) pattern of the Cer[EOS]/Cer[NS]/FFA/Chol mixture at the molar ratio of 0.3/0.7/1/0.45 at 40 % ^2^H_2_O in H_2_O (v/v) at 100 % relative humidity (RH) and T = 32 °C. The asterisk indicates the peak of the separated Chol.


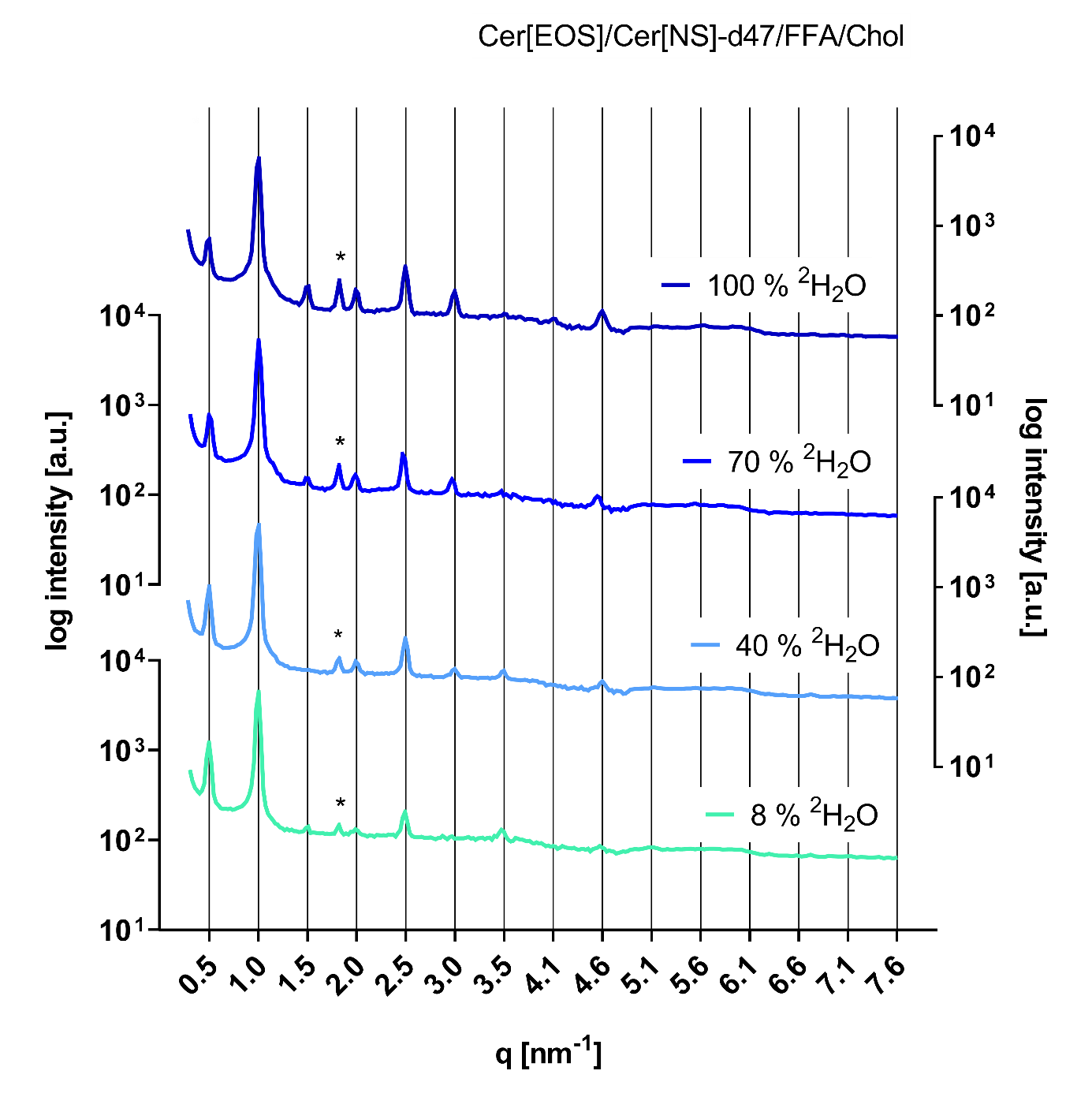


**Supplemental Figure S5.** The ND patterns of the Cer[EOS]/Cer[NS]-d47/FFA/Chol mixture at the molar ratio of 0.3/0.7/1/0.45 under the indicated contrast condition (% of ^2^H_2_O in H_2_O; v/v) at 100 % RH and T = 32 °C. The asterisks indicate the peaks of the separated Chol.

**Supplemental Figure S6.** The ND form factors *F(h)* of the Cer[EOS]/Cer[NS]-d47/FFA/Chol sample at the molar ratio of 0.3/0.7/1/0.45 in dependence on the % of ^2^H_2_O in H_2_O (v/v) evaluated according to (6).


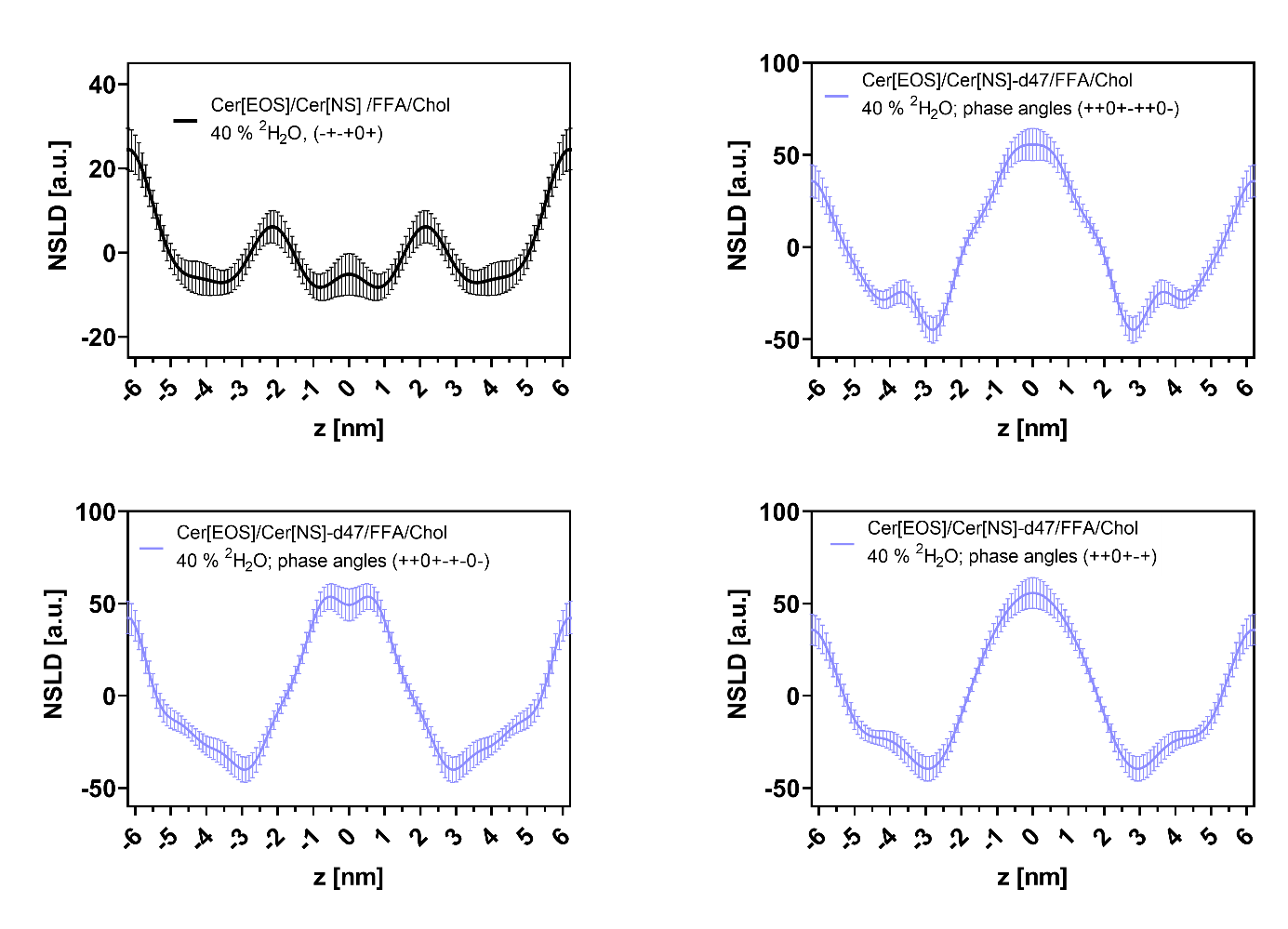


**Supplemental Figure S7.** The relative neutron scattering length density (NSLD) profiles of the Cer[EOS]/Cer[NS]/FFA/Chol and Cer[EOS]/Cer[NS]-*d*_47_/FFA/Chol samples reconstructed from the form factors *F(h)* with the indicated phase angles at 40 % of ^2^H_2_O in H_2_O (v/v). The phase angles for the protonated sample were adopted from Mojumdar et. al. (8). The phase angles for the deuterated sample were determined from the slopes of the *F(h)* vs. % ^2^H_2_O functions according to Franks and Lieb (6). Three variants of the NSLD profiles of the deuterated sample were reconstructed. They differ either in the number of the included *F(h)* or in the phase angle of the *F(7)* order, which determination was uncertain.


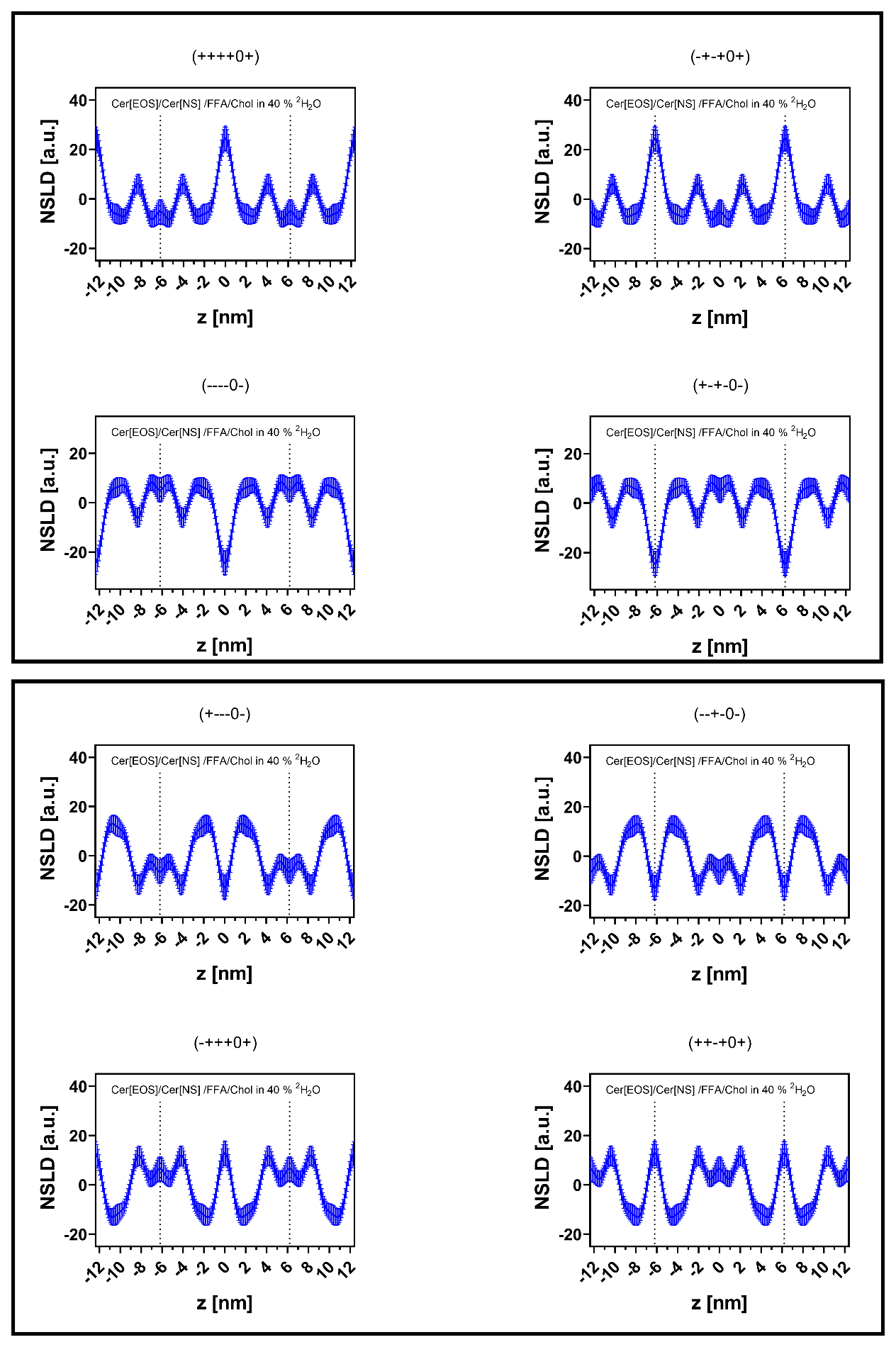


**Supplemental Figure S8a**. The relative NSLD profiles of the Cer[EOS]/Cer[NS]/FFA/Chol sample reconstructed from the form factors *F(h)* with the indicated phase angles at 40 % of ^2^H_2_O in H_2_O (v/v). The profiles placed in the same panel are either shifted by *d*/2 or inverted. The dotted lines indicate the repeat distance *d* [nm].


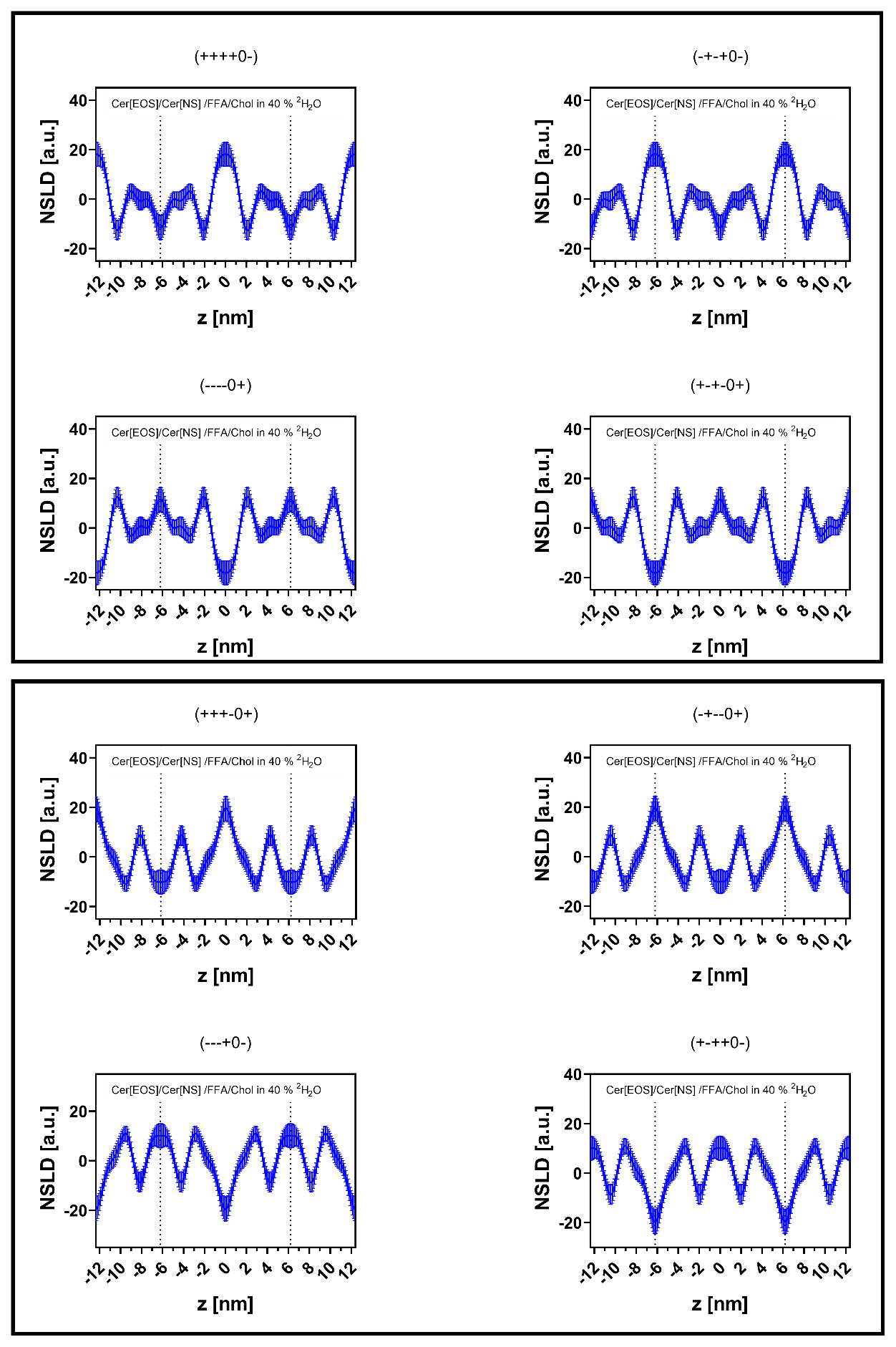


**Supplemental Figure S8b**. The relative NSLD profiles of the Cer[EOS]/Cer[NS]/FFA/Chol sample reconstructed from the form factors *F(h)* with the indicated phase angles at 40 % of ^2^H_2_O in H_2_O (v/v). The profiles placed in the same panel are either shifted by *d*/2 or inverted. The dotted lines indicate the repeat distance *d* [nm].


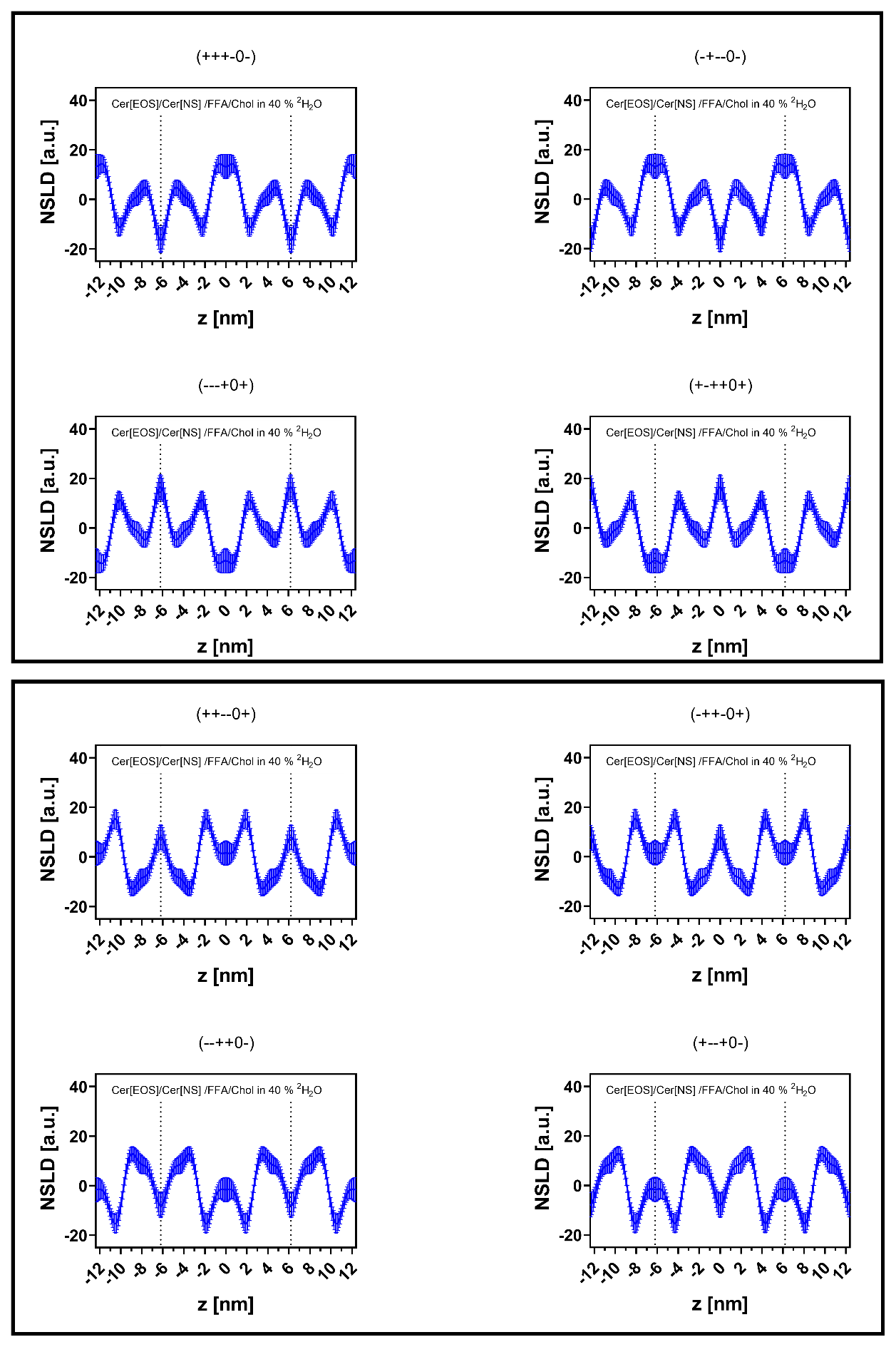


**Supplemental Figure S8c**. The relative NSLD profiles of the Cer[EOS]/Cer[NS]/FFA/Chol sample reconstructed from the form factors *F(h)* with the indicated phase angles at 40 % of ^2^H_2_O in H_2_O (v/v). The profiles placed in the same panel are either shifted by *d*/2 or inverted. The dotted lines indicate the repeat distance *d* [nm].


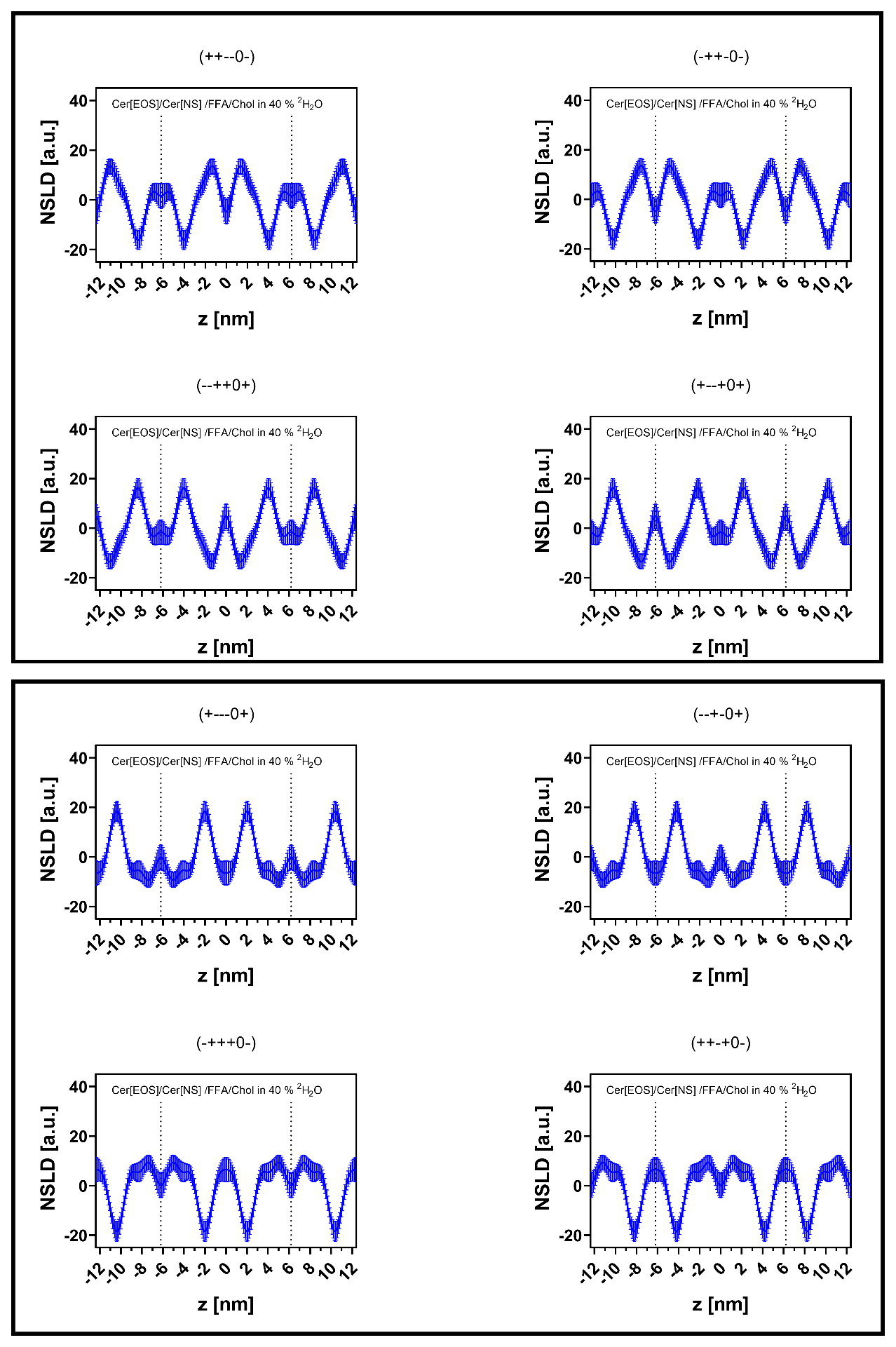


**Supplemental Figure S8d**. The relative NSLD profiles of the Cer[EOS]/Cer[NS]/FFA/Chol sample reconstructed from the form factors *F(h)* with the indicated phase angles at 40 % of ^2^H_2_O in H_2_O (v/v). The profiles placed in the same panel are either shifted by *d*/2 or inverted. The dotted lines indicate the repeat distance *d* [nm].


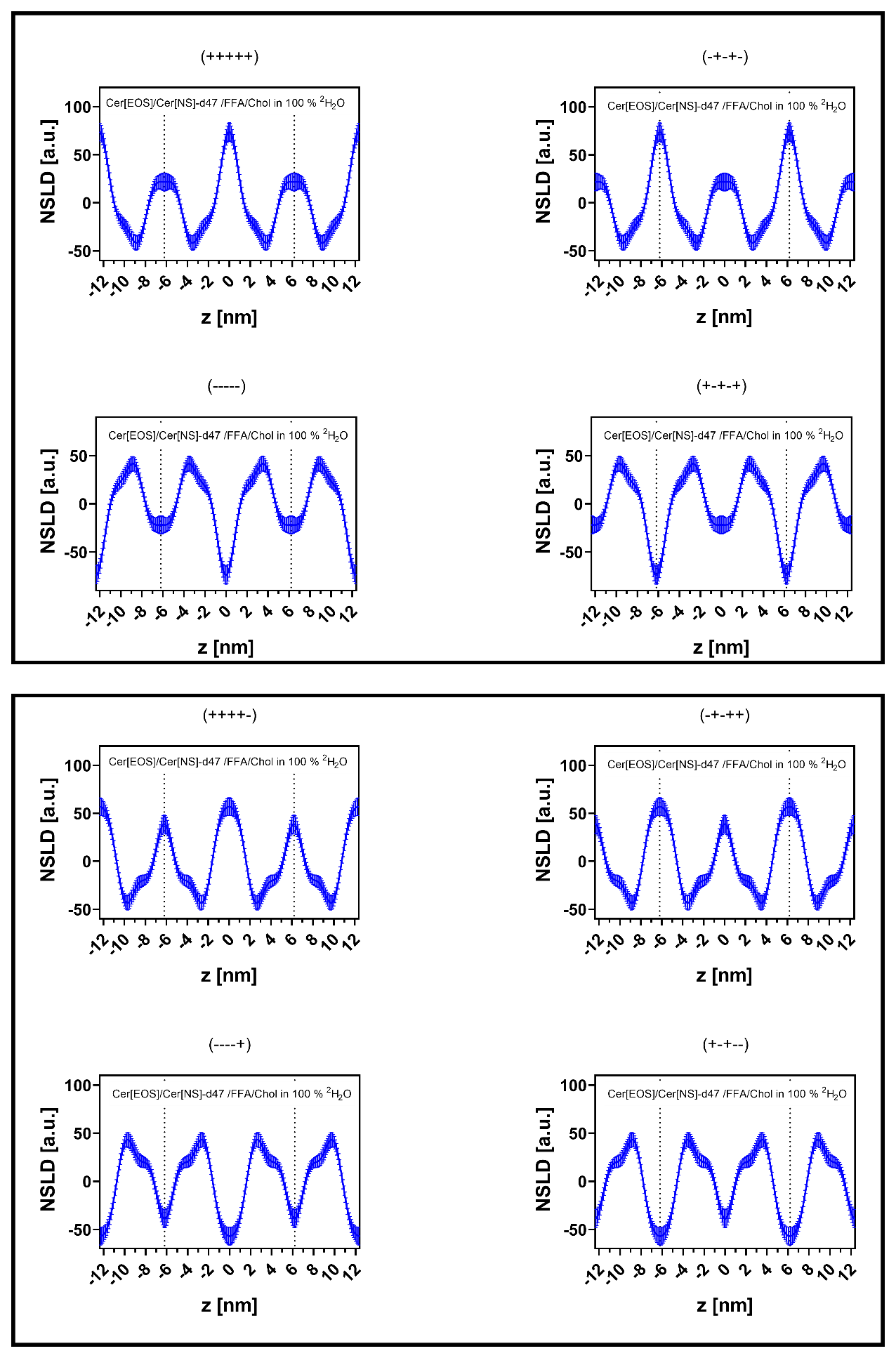


**Supplemental Figure S9a.** The relative NSLD profiles of the Cer[EOS]/Cer[NS]-*d*_47_/FFA/Chol sample reconstructed from the form factors *F(h)* with the indicated phase angles at 100 % of ^2^H_2_O in H_2_O (v/v). The profiles placed in the same panel are either shifted by *d*/2 or inverted. The dotted lines indicate the repeat distance *d* [nm].


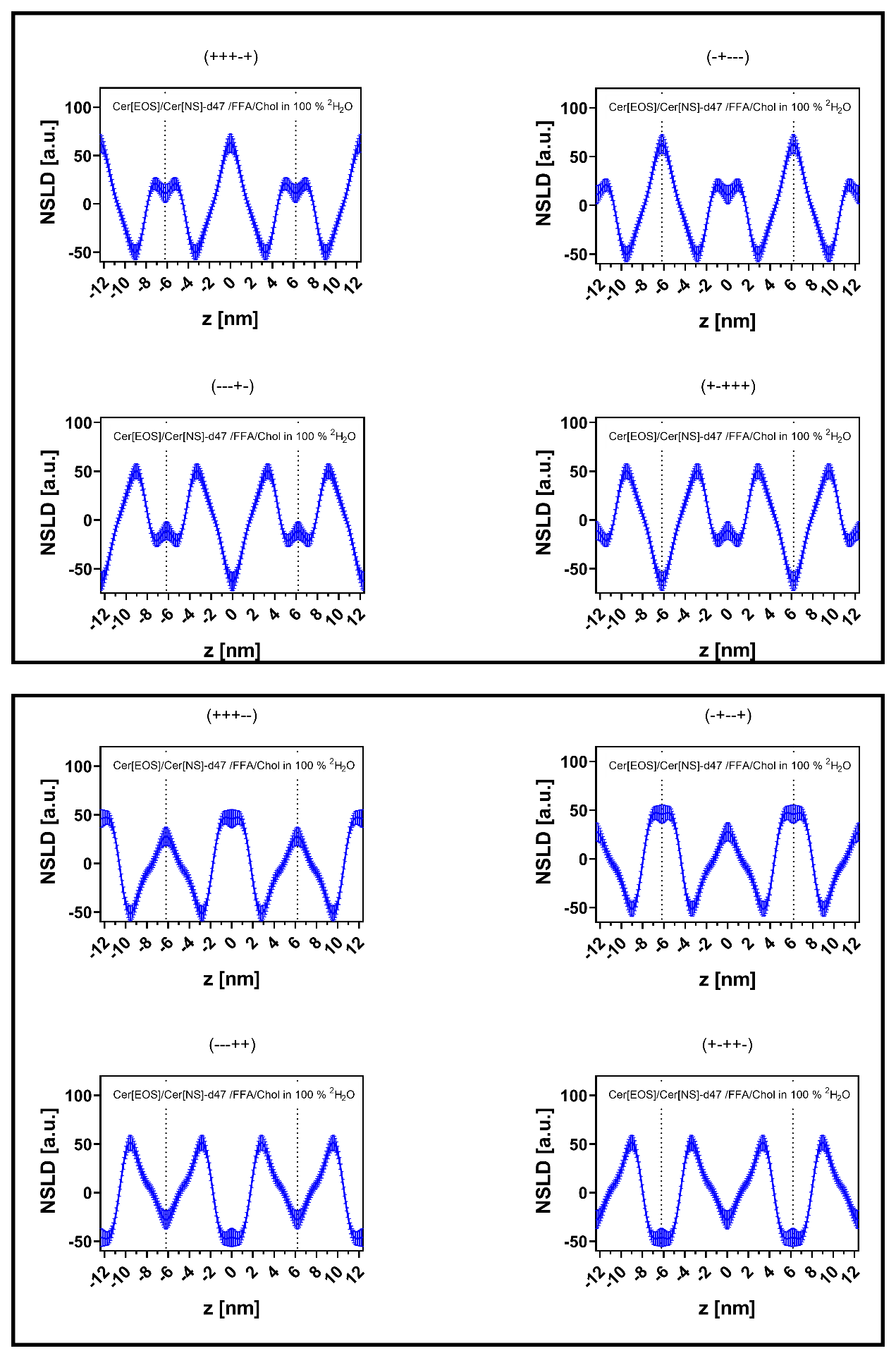


**Supplemental Figure S9b.** The relative NSLD profiles of the Cer[EOS]/Cer[NS]-*d*_47_/FFA/Chol sample reconstructed from the form factors *F(h)* with the indicated phase angles at 100 % of ^2^H_2_O in H_2_O (v/v). The profiles placed in the same panel are either shifted by *d*/2 or inverted. The dotted lines indicate the repeat distance *d* [nm].


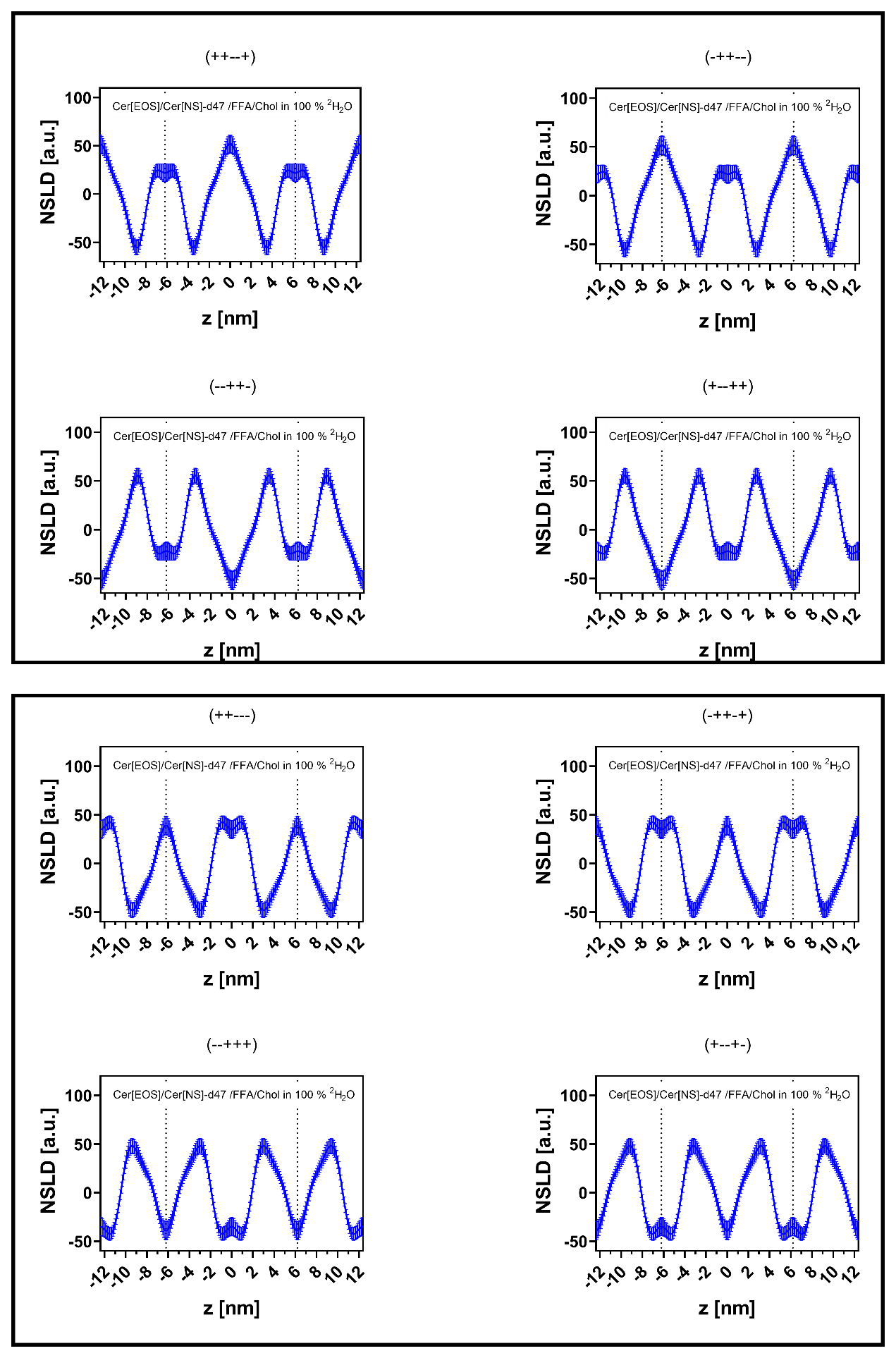


**Supplemental Figure S9c**. The relative NSLD profiles of the Cer[EOS]/Cer[NS]-*d*_47_/FFA/Chol sample reconstructed from the form factors *F(h)* with the indicated phase angles at 100 % of ^2^H_2_O in H_2_O (v/v). The profiles placed in the same panel are either shifted by *d*/2 or inverted. The dotted lines indicate the repeat distance *d* [nm].


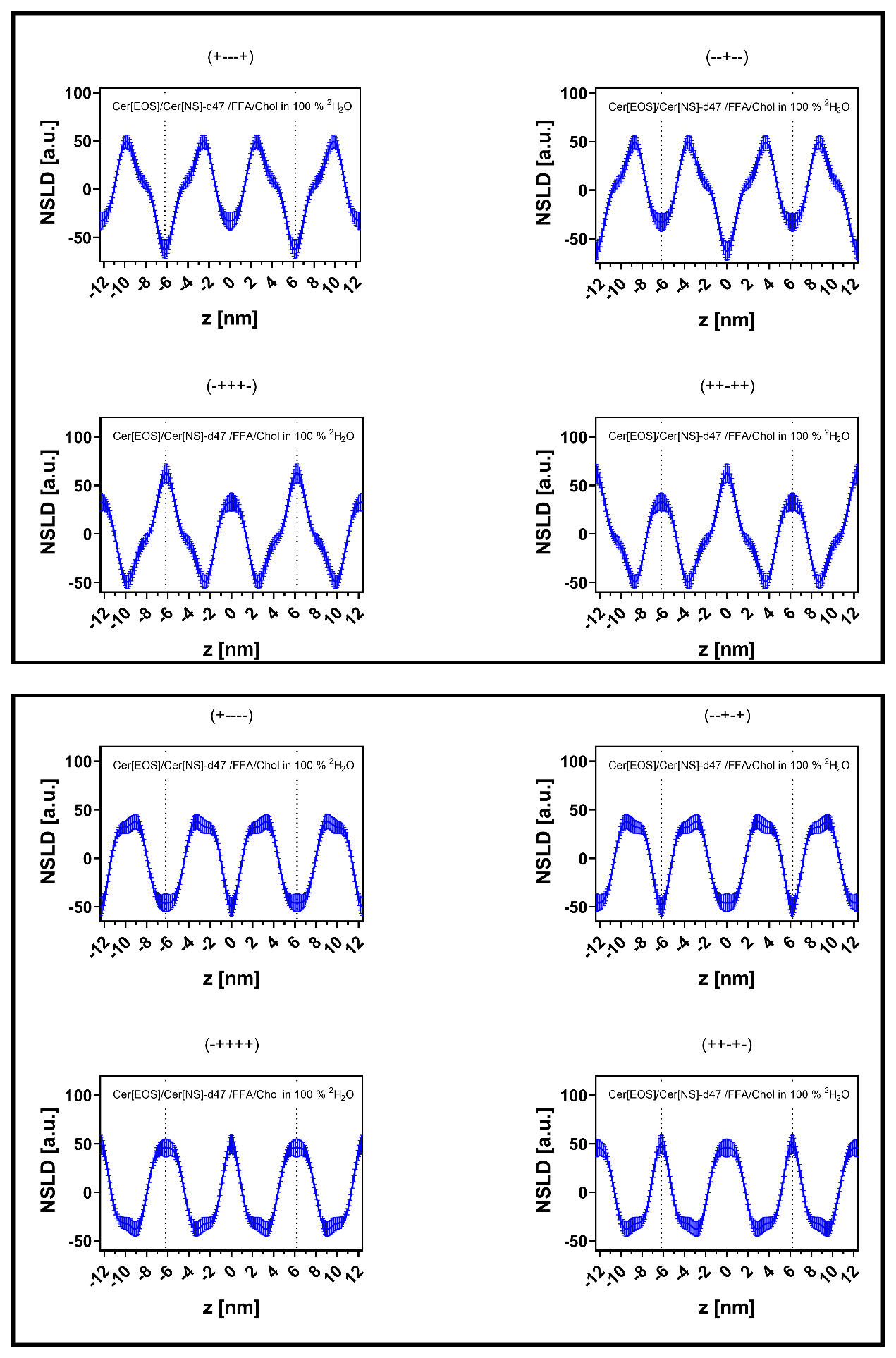


**Supplemental Figure S9d.** The relative NSLD profiles of the Cer[EOS]/Cer[NS]-*d*_47_/FFA/Chol sample reconstructed from the form factors *F(h)* with the indicated phase angles at 100 % of ^2^H_2_O in H_2_O (v/v). The profiles placed in the same panel are either shifted by *d*/2 or inverted. The dotted lines indicate the repeat distance *d* [nm].


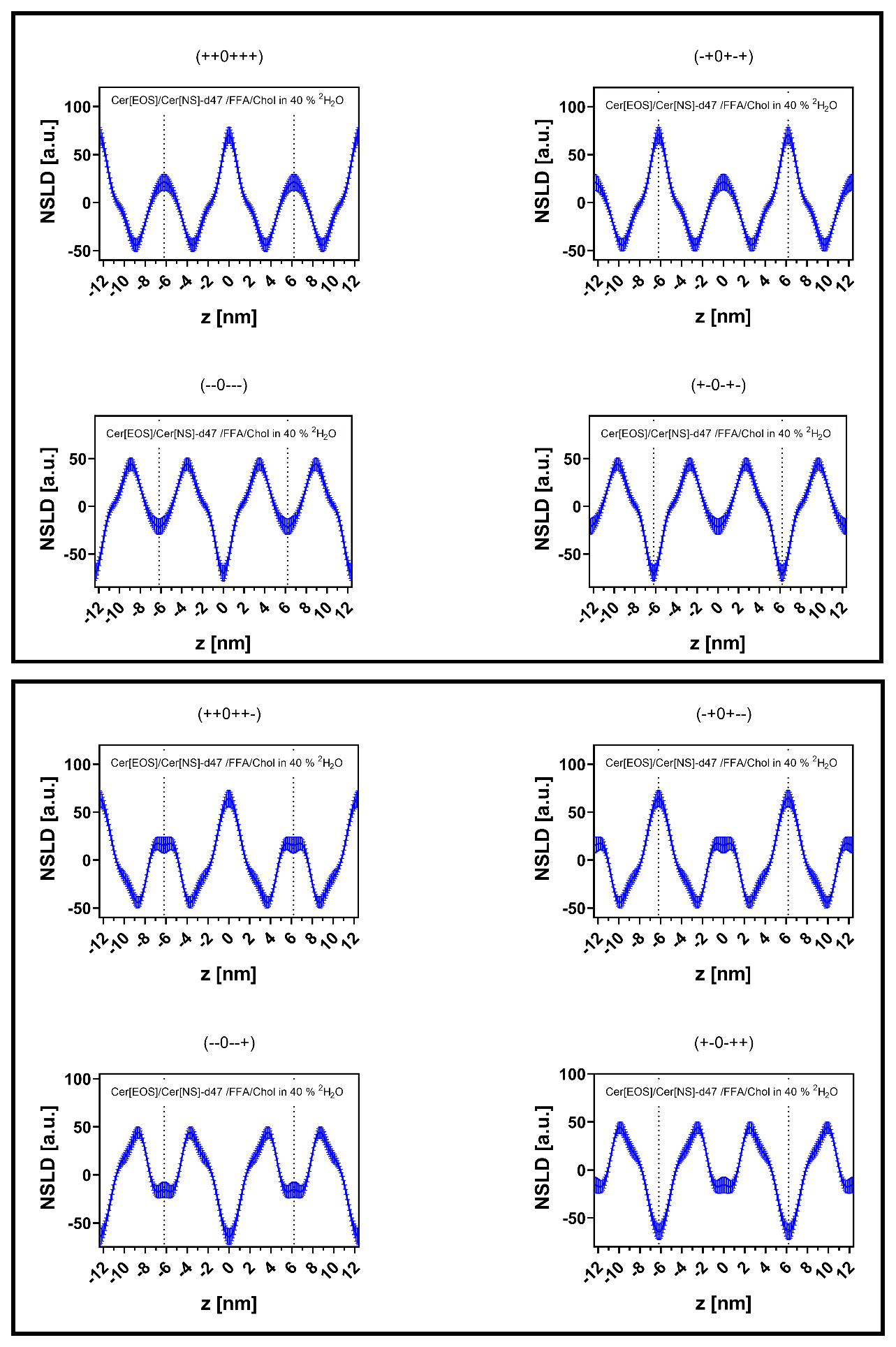


**Supplemental Figure S10a.** The relative NSLD profiles of the Cer[EOS]/Cer[NS]-*d*_47_/FFA/Chol sample reconstructed from the form factors *F(h)* with the indicated phase angles at 40 % of ^2^H_2_O in H_2_O (v/v). The profiles placed in the same panel are either shifted by *d*/2 or inverted. The dotted lines indicate the repeat distance *d* [nm].


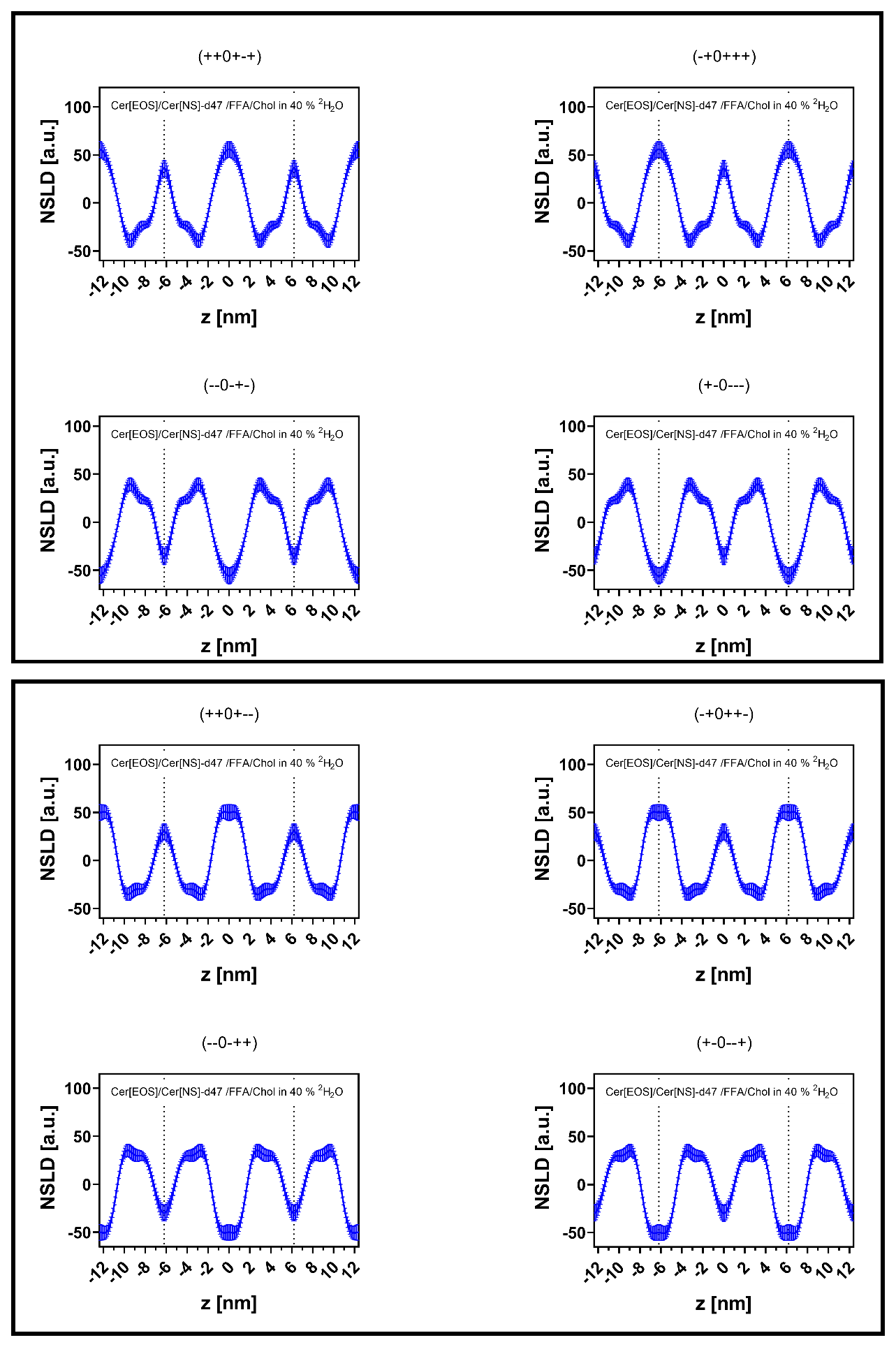


**Supplemental Figure S10b**. The relative NSLD profiles of the Cer[EOS]/Cer[NS]-*d*_47_/FFA/Chol sample reconstructed from the form factors *F(h)* with the indicated phase angles at 40 % of ^2^H_2_O in H_2_O (v/v). The profiles placed in the same panel are either shifted by *d*/2 or inverted. The dotted lines indicate the repeat distance *d* [nm].


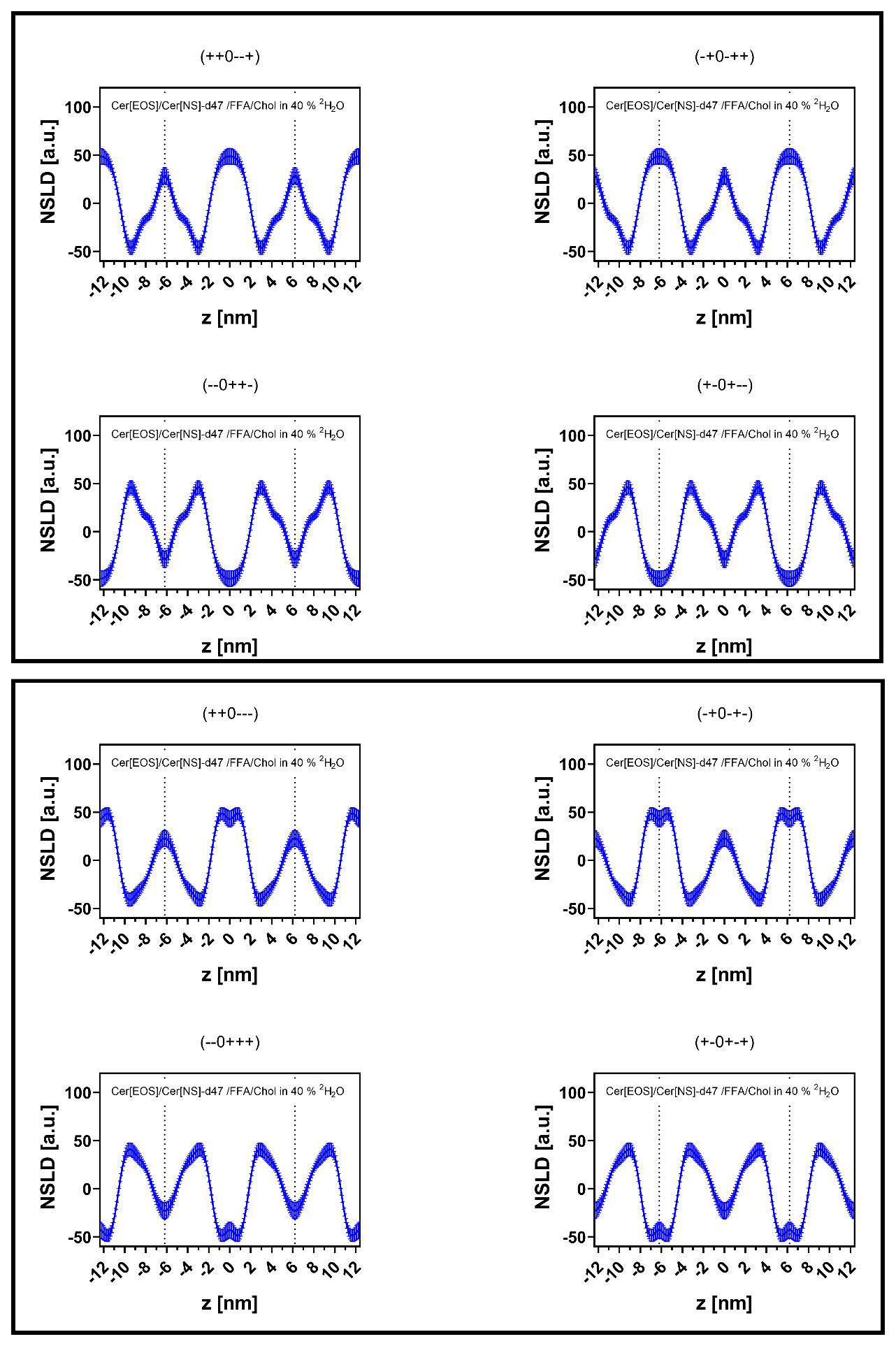


**Supplemental Figure S10c**. The relative NSLD profiles of the Cer[EOS]/Cer[NS]-*d*_47_/FFA/Chol sample reconstructed from the form factors *F(h)* with the indicated phase angles at 40 % of ^2^H_2_O in H_2_O (v/v). The profiles placed in the same panel are either shifted by *d*/2 or inverted. The dotted lines indicate the repeat distance *d* [nm].


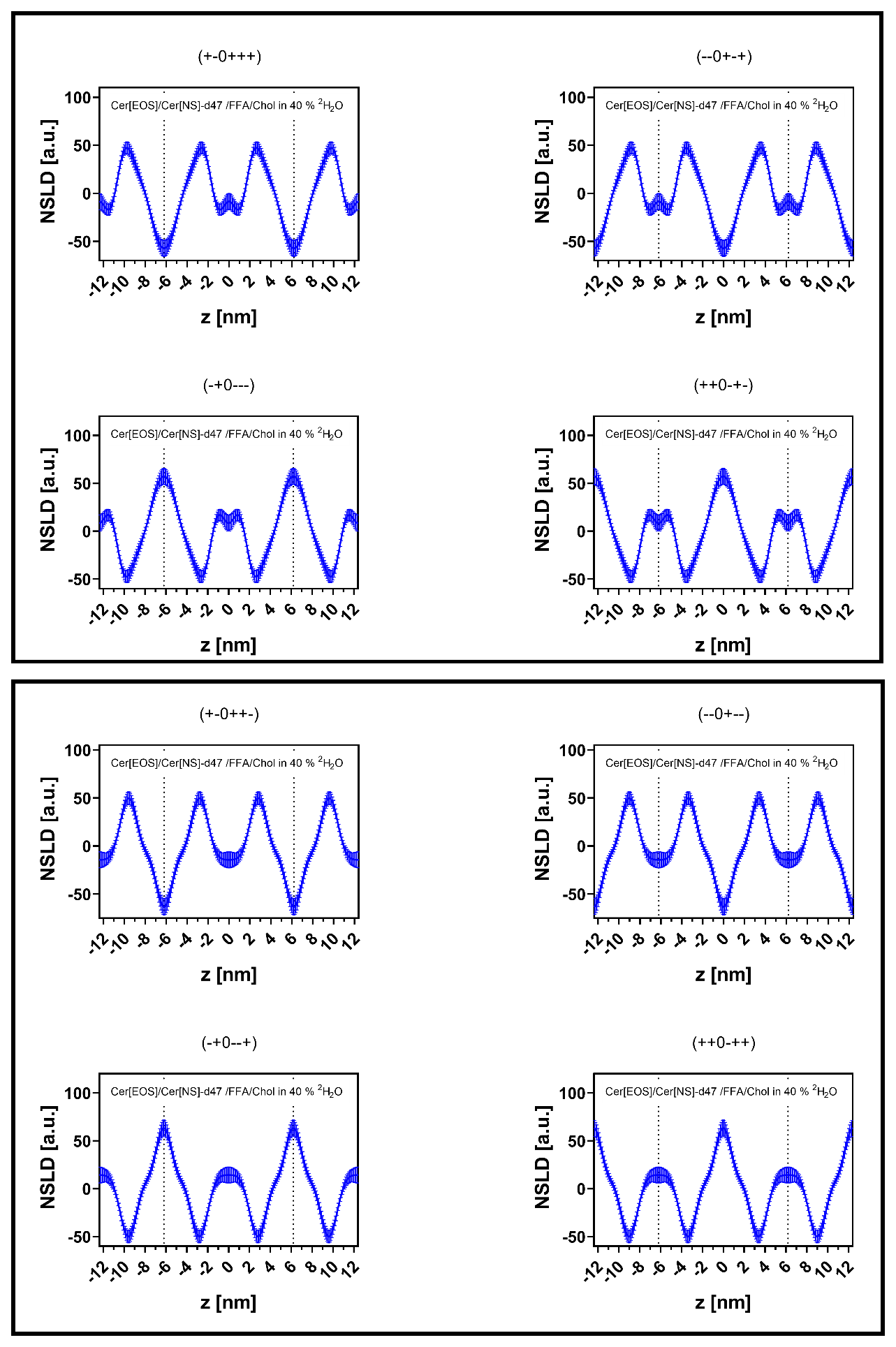


**Supplemental Figure S10d**. The relative NSLD profiles of the Cer[EOS]/Cer[NS]-*d*_47_/FFA/Chol sample reconstructed from the form factors *F(h)* with the indicated phase angles at 40 % of ^2^H_2_O in H_2_O (v/v). The profiles placed in the same panel are either shifted by *d*/2 or inverted. The dotted lines indicate the repeat distance *d* [nm].


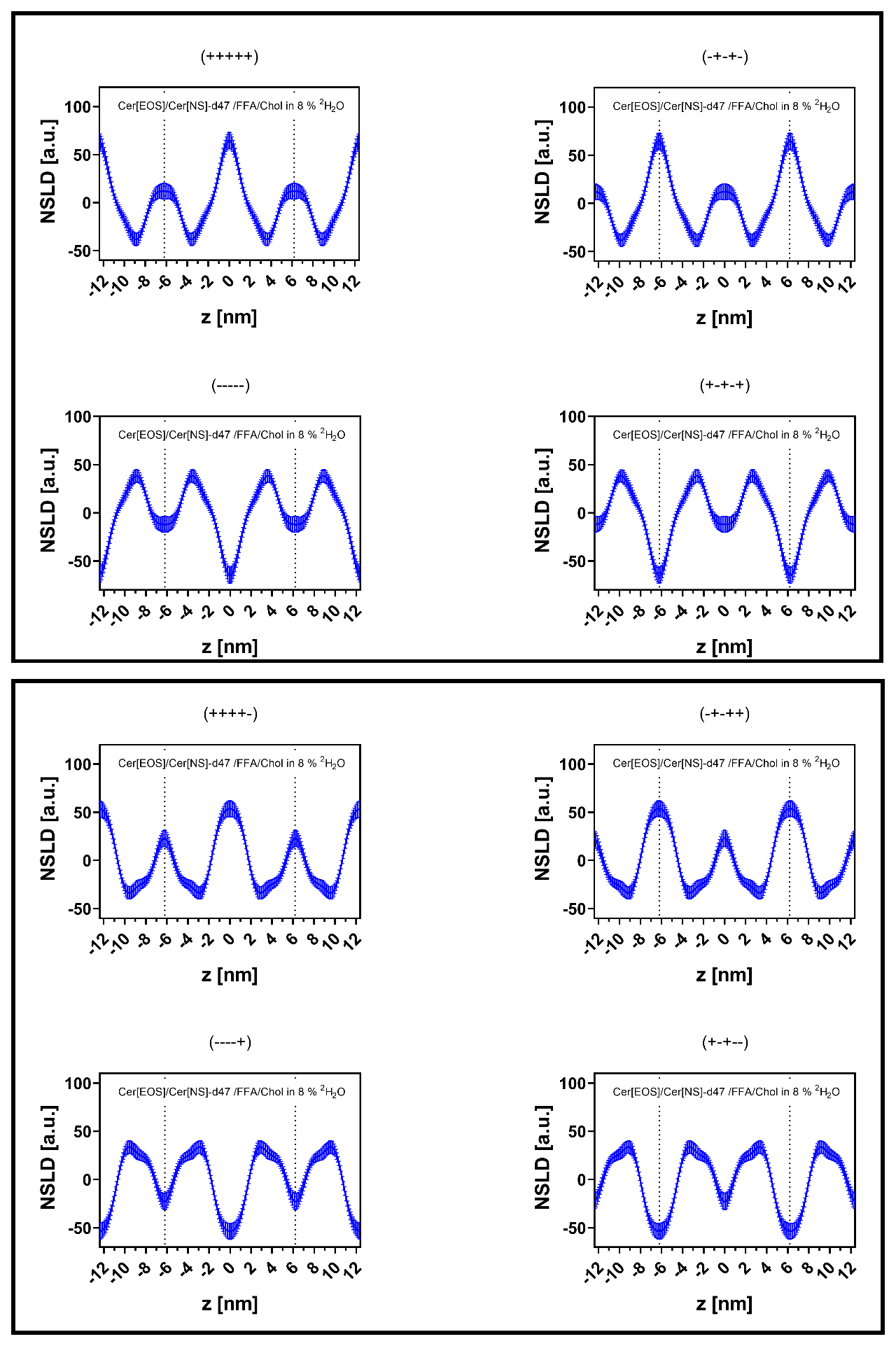


**Supplemental Figure S11a**. The relative NSLD profiles of the Cer[EOS]/Cer[NS]-*d*_47_/FFA/Chol sample reconstructed from the form factors *F(h)* with the indicated phase angles at 8 % of ^2^H_2_O in H_2_O (v/v). The profiles placed in the same panel are either shifted by *d*/2 or inverted. The dotted lines indicate the repeat distance *d* [nm].


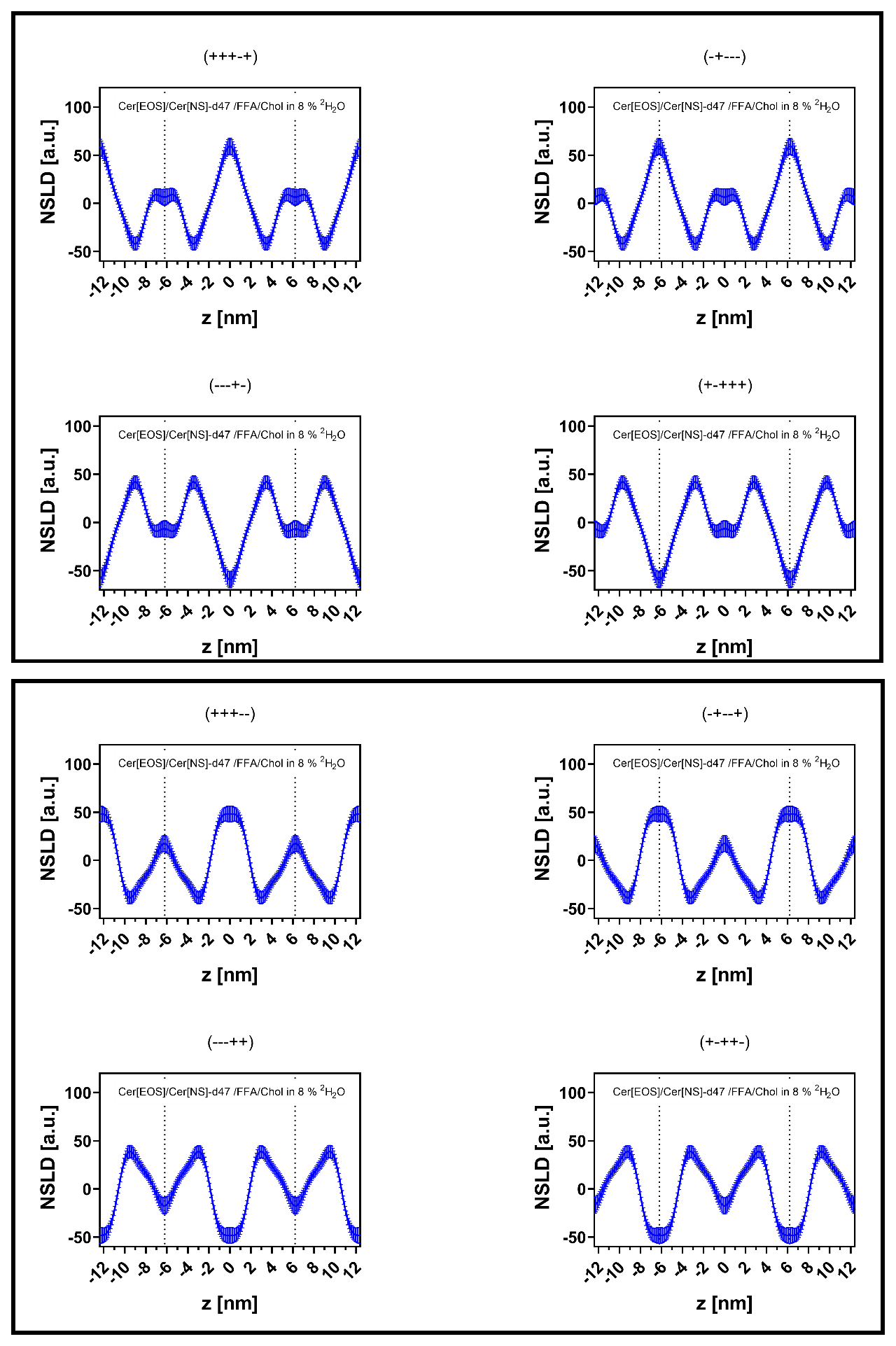


**Supplemental Figure S11b**. The relative NSLD profiles of the Cer[EOS]/Cer[NS]-*d*_47_/FFA/Chol sample reconstructed from the form factors *F(h)* with the indicated phase angles at 8 % of ^2^H_2_O in H_2_O (v/v). The profiles placed in the same panel are either shifted by *d*/2 or inverted. The dotted lines indicate the repeat distance *d* [nm].


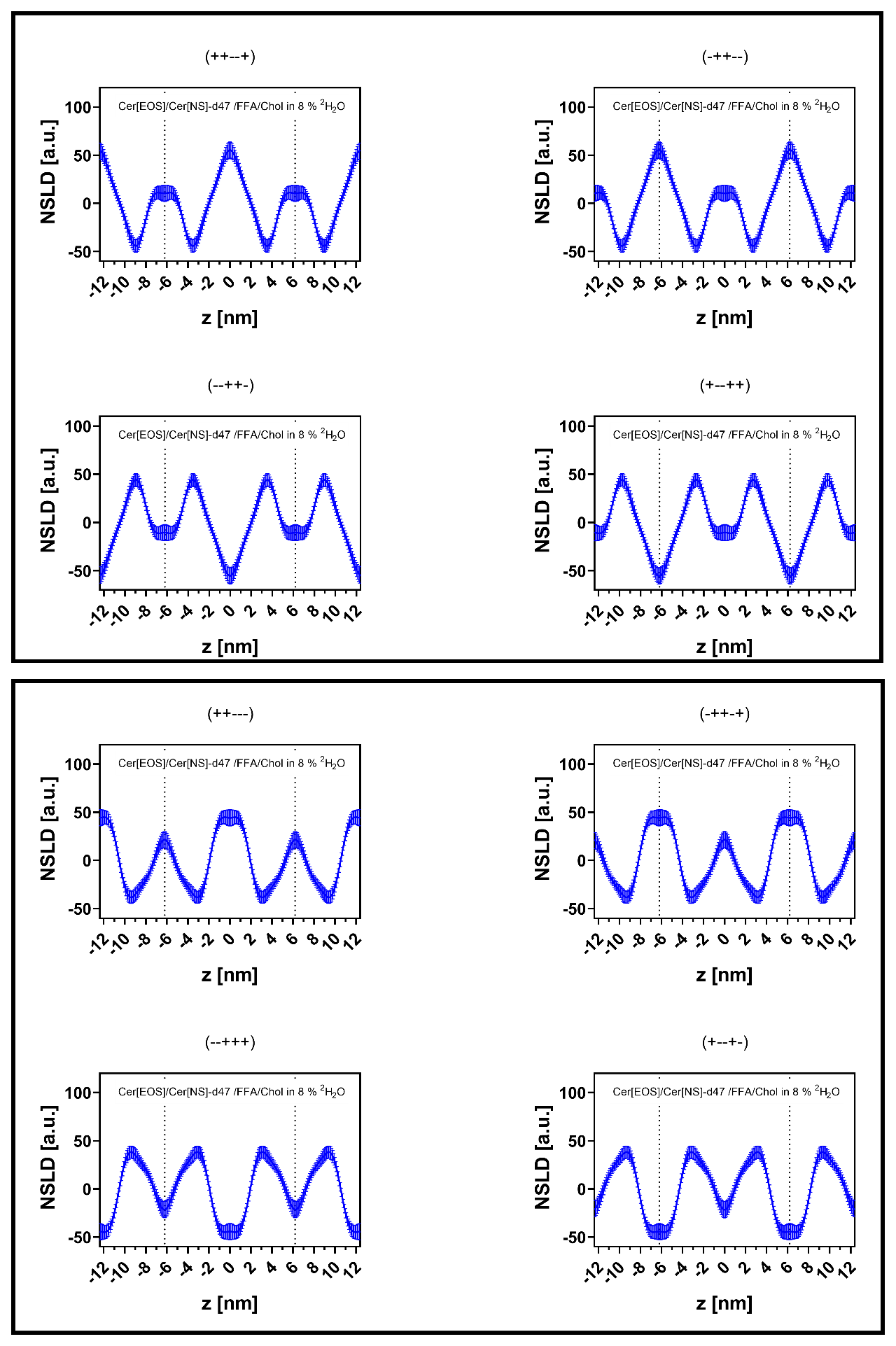


**Supplemental Figure S11c**. The relative NSLD profiles of the Cer[EOS]/Cer[NS]-*d*_47_/FFA/Chol sample reconstructed from the form factors *F(h)* with the indicated phase angles at 8 % of ^2^H_2_O in H_2_O (v/v). The profiles placed in the same panel are either shifted by *d*/2 or inverted. The dotted lines indicate the repeat distance *d* [nm].


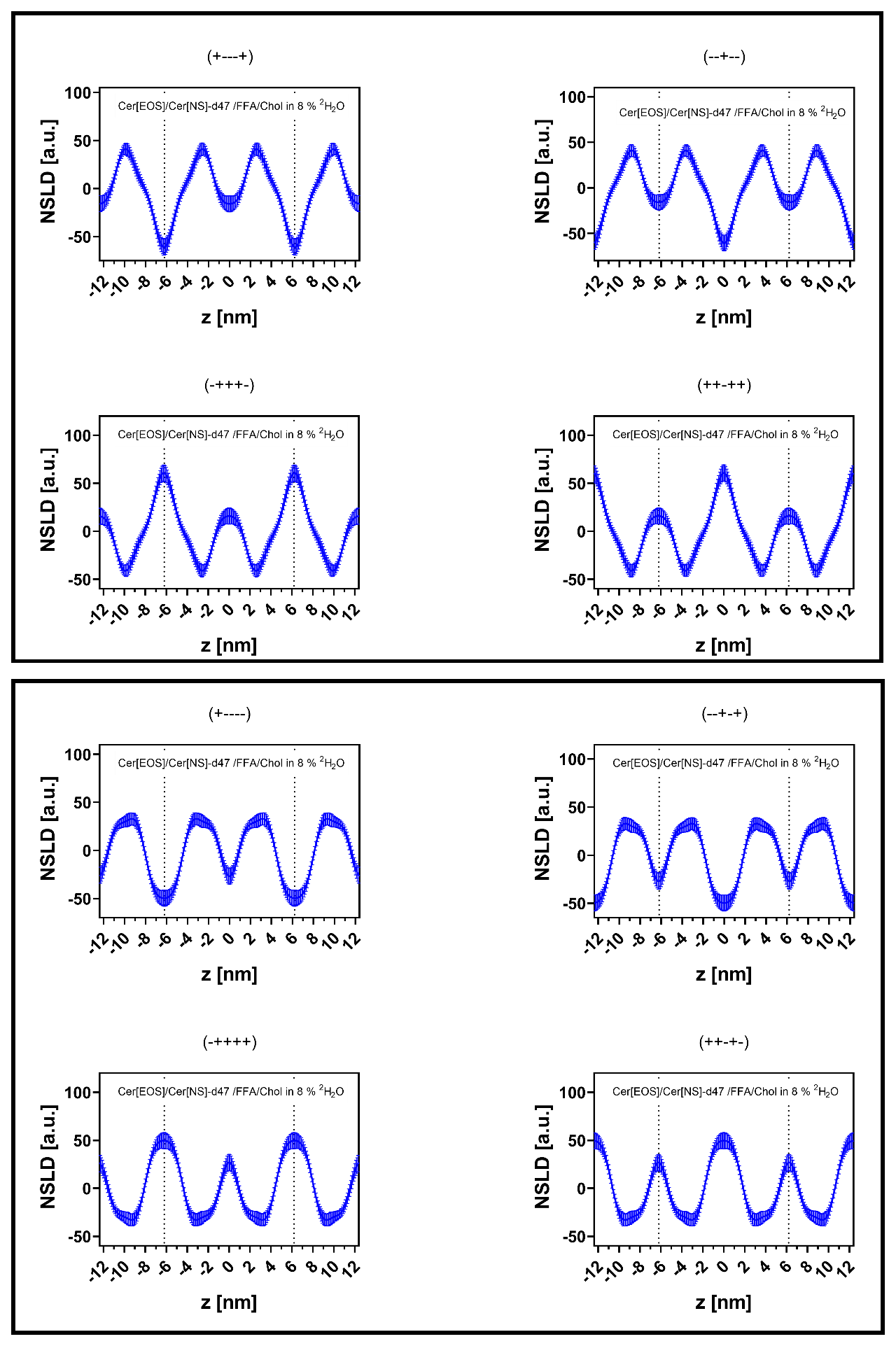


**Supplemental Figure S11d.** The relative NSLD profiles of the Cer[EOS]/Cer[NS]-*d*_47_/FFA/Chol sample reconstructed from the form factors *F(h)* with the indicated phase angles at 8 % of ^2^H_2_O in H_2_O (v/v). The profiles placed in the same panel are either shifted by *d*/2 or inverted. The dotted lines indicate the repeat distance *d* [nm].

**References**

1. Opálka L, Kováčik A, Sochorová M, Roh J, Kuneš J, Lenčo J, et al. Scalable Synthesis of Human Ultralong Chain Ceramides. Org Lett. 2015;17(21):5456-9.

2. Smith AA. INFOS: spectrum fitting software for NMR analysis. J Biomol NMR. 2017;67(2):77-94.

3. Kucerka N, Nieh MP, Pencer J, Sachs JN, Katsaras J. What determines the thickness of a biological membrane. Gen Physiol Biophys. 2009;28(2):117-25.

4. Groen D, Gooris GS, Barlow DJ, Lawrence MJ, van Mechelen JB, Deme B, et al. Disposition of ceramide in model lipid membranes determined by neutron diffraction. Biophys J. 2011;100(6):1481-9.

5. Nagle JF, Akabori K, Treece BW, Tristram-Nagle S. Determination of mosaicity in oriented stacks of lipid bilayers. Soft Matter. 2016;12(6):1884-91.

6. Franks NP, Lieb WR. The structure of lipid bilayers and the effects of general anaesthetics. An x-ray and neutron diffraction study. J Mol Biol. 1979;133(4):469-500.

7. Kirschner DA, Sidman RL. X-ray diffraction study of myelin structure in immature and mutant mice. Biochim Biophys Acta. 1976;448(1):73-87.

8. Mojumdar EH, Gooris GS, Barlow DJ, Lawrence MJ, Deme B, Bouwstra JA. Skin lipids: localization of ceramide and fatty acid in the unit cell of the long periodicity phase. Biophys J. 2015;108(11):2670-9.

9. Coleman TF, Li Y. An interior trust region approach for nonlinear minimization subject to bounds. SIAM J Optim*.* 1996;6:418–45.

10. Coleman TF, Li Y. A reflective Newton method for minimizing a quadratic function subject to bounds on some of the variables. SIAM J Optim. 1996;**6**:1040–58.
